# Cerebellum-Specific Deletion of the GABA_A_ Receptor δ Subunit Leads to Sex-Specific Disruption of Behavior

**DOI:** 10.1016/j.celrep.2020.108338

**Published:** 2020-11-03

**Authors:** Stephanie Rudolph, Chong Guo, Stan L. Pashkovski, Tomas Osorno, Winthrop F. Gillis, Jeremy M. Krauss, Hajnalka Nyitrai, Isabella Flaquer, Mahmoud El-Rifai, Sandeep Robert Datta, Wade G. Regehr

**Affiliations:** 1 Department of Neurobiology, Harvard Medical School, Boston, MA 02115, USA; 2 Present address: Albert Einstein College of Medicine, New York, NY 10461, USA; 3 Lead Contact

## Abstract

Granule cells (GCs) of the cerebellar input layer express high-affinity δ GABA_A_ subunit-containing GABA_A_ receptors (δGABA_A_Rs) that respond to ambient GABA levels and context-dependent neuromodulators like steroids. We find that GC-specific deletion of δGABA_A_ (cerebellar [cb] δ knockout [KO]) decreases tonic inhibition, makes GCs hyperexcitable, and in turn, leads to differential activation of cb output regions as well as many cortical and subcortical brain areas involved in cognition, anxiety-like behaviors, and the stress response. Cb δ KO mice display deficits in many behaviors, but motor function is normal. Strikingly, δGABA_A_ deletion alters maternal behavior as well as spontaneous, stress-related, and social behaviors specifically in females. Our findings establish that δGABA_A_Rs enable the cerebellum to control diverse behaviors not previously associated with the cerebellum in a sex-dependent manner. These insights may contribute to a better understanding of the mechanisms that underlie behavioral abnormalities in psychiatric and neurodevelopmental disorders that display a gender bias.

## In Brief

Rudolph et al. show that deletion of the neuromodulator and hormone-sensitive δGABA_A_ receptor subunit from cerebellar granule cells results in anxiety-like behaviors and female-specific deficits in social behavior and maternal care. δGABA_A_ deletion is associated with hyperexcitability of the cerebellar input layer and altered activation of many stress-related brain regions.

## Graphical Abstract

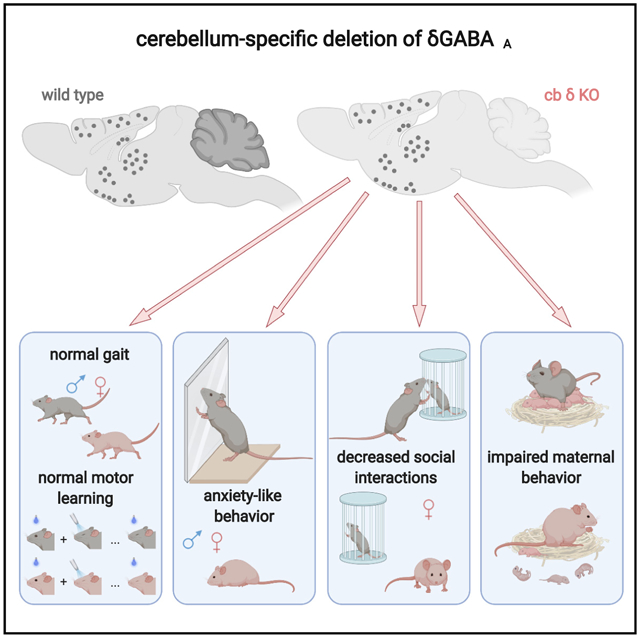

## INTRODUCTION

Normal brain function requires GABAergic inhibition (Ferguson and Gao, 2018; Wood et al., 2017). GABA_A_ receptor-mediated inhibition occurs on different timescales and can consist of rapid (phasic) and persistent (tonic) components (Brickley et al., 1996; Farrant and Nusser, 2005; Rossi et al., 2003). Tonic inhibition influences neuronal membrane properties, such as membrane potential and resistance, and, consequently, controls excitability and synaptic integration (Cavelier et al., 2005; Duguid et al., 2012; Mitchell and Silver, 2003; Semyanov et al., 2004). Typically, tonic inhibition is mediated by GABA_A_ receptors containing δGABA_A_ subunits (δGABA_A_Rs), which are high affinity, slowly desensitizing, extrasynaptically located, and sensitive to fluctuations in ambient GABA levels and neuromodulators (Farrant and Nusser, 2005; Glykys et al., 2008; Martenson et al., 2017; Mihalek et al., 1999; Spigelman et al., 2003; Stell and Mody, 2002; Stell et al., 2003; Vicini et al., 2002; Wei et al., 2003). δGABA_A_Rs are thus poised to respond to context-dependent signals that are essential for controlling behavior. δGABA_A_Rs are involved in diverse physiological and pathophysiological processes, such as learning and memory; anxiety; stress; sleep; pain; seizures; psychiatric and neurodevelopmental disorders (Hines et al., 2012; Whissell et al., 2015), including attention deficit hyperactivity disorder (ADHD) and autism spectrum disorder (ASD) (Bridi et al., 2017; Martin et al., 2014; Zhang et al., 2017a); pregnancy and maternal behaviors (Maguire and Mody, 2008; Maguire et al., 2009); and estrous cycle-dependent fluctuations in mood and seizure susceptibility (Carver et al., 2014; Maguire et al., 2005; Smith et al., 2006). δGABA_A_Rs have therefore garnered significant interest as potential pharmacological targets for numerous disorders, including postpartum depression (Melón et al., 2018; Meltzer-Brody et al., 2018), epilepsy (Petersen et al., 1983), trauma, panic and anxiety disorders (Rasmusson et al., 2017), insomnia (Orser, 2006; Thakkar et al., 2008), and some syndromic forms of ASD, such as fragile X syndrome (FXS) (Braat and Kooy, 2015; Cogram et al., 2019; Modgil et al., 2019; Olmos-Serrano et al., 2011).

Global δGABA_A_ knockout (global δ KO) mice show diverse behavioral deficits (Maguire and Mody, 2008; Mihalek et al., 2001; Spigelman et al., 2002; Wiltgen et al., 2005), but the brain regions involved in these deficits are unknown. δGABA_A_Rs are present in many brain regions, however, most attention has focused on δGABA_A_s in the hippocampus, hypothalamus, amygdala, and cortex, regions that have classically been associated with fear and anxiety-related behaviors, stress, and cognition. In contrast, even though the cerebellum shows the highest expression levels of δGABA_A_ (Jones et al., 1997; Pirker et al., 2000; Poulter et al., 1992; Wisden et al., 1992), it has been speculated that cerebellar δGABA_A_Rs contribute to motor function rather than non-motor behaviors (Whissell et al., 2015). However, many studies have demonstrated that the cerebellum controls cognitive, social, and emotional processes (Buckner, 2013; Schmahmann et al., 2019; Stoodley and Schmahmann, 2018; Strick et al., 2009). In addition, many studies have found that cerebellar disruption is associated with psychiatric and neurodevelopmental disorders (Becker and Stoodley, 2013; Fatemi et al., 2012; Kloth et al., 2015; Sathyanesan et al., 2019; Stoodley et al., 2017; Tsai et al., 2012, 2018). Thus, there is considerable overlap in behavioral deficits and disorders that involve the cerebellum and δGABA_A_Rs, suggesting that δGABA_A_Rs in cerebellar granule cells (GCs) could contribute to these behaviors.

In the cerebellar input layer, mossy fibers carry multimodal sensory information from diverse cortical, subcortical, and peripheral regions and excite GCs (Chabrol et al., 2015; Huang et al., 2013; Ishikawa et al., 2015; Witter and De Zeeuw, 2015). Golgi cells provide ambient GABA that tonically inhibits GCs by activating extrasynaptic GABA_A_Rs containing the δ and α6 subunits (Jechlinger et al., 1998). δGABA_A_Rs are therefore well situated to regulate the excitability of the input layer. However, in previous studies, removing the α6GABA_A_ subunit eliminated tonic inhibition but left excitability unaltered because of compensatory potassium channel expression (Brickley et al., 2001). The behavioral effects of GC hyperexcitability thus remained elusive. How input layer hyperexcitability controls behavior is of particular interest, given the recent observations of dense GC activation during certain behaviors (Badura and De Zeeuw, 2017; Cayco-Gajic and Silver, 2019; Giovannucci et al., 2017; Wagner et al., 2017) that contradict classic models of the cerebellar computation that relied on sparse coding (Marr, 1969; Albus, 1971). Thus, it remains to be determined whether elimination of δGABA_A_ alters GC excitability and whether excitability changes affect behavior.

We examined the effects of GC-specific deletion of δGABA_A_ on physiology and behavior. We found that tonic inhibition in GCs lacking δGABA_A_ is reduced, leading to hyperexcitability. Despite profound excitability changes in the cerebellar input layer, motor function was unaffected in cerebellar (cb) δ KO mice. Remarkably, cb δ KO mice displayed deficits in diverse behaviors not previously associated with the cerebellum, and many were selectively altered in cb δ KO females, including reduced sociability and abnormal maternal behavior. We also found that GC-selective deletion of δGABA_A_ resulted in differential activation of many cortical and subcortical brain areas involved in cognition, anxiety-like behaviors, and the stress response. These findings establish that the molecular make-up of the cerebellum, sensitive to sex and stress-related signals, enables modulation of behavior in a sex-specific manner. These insights may contribute to a better understanding of the mechanisms that underlie behavioral abnormalities in psychiatric and neurodevelopmental disorders that display a sex bias.

## RESULTS

**Figure 1.**
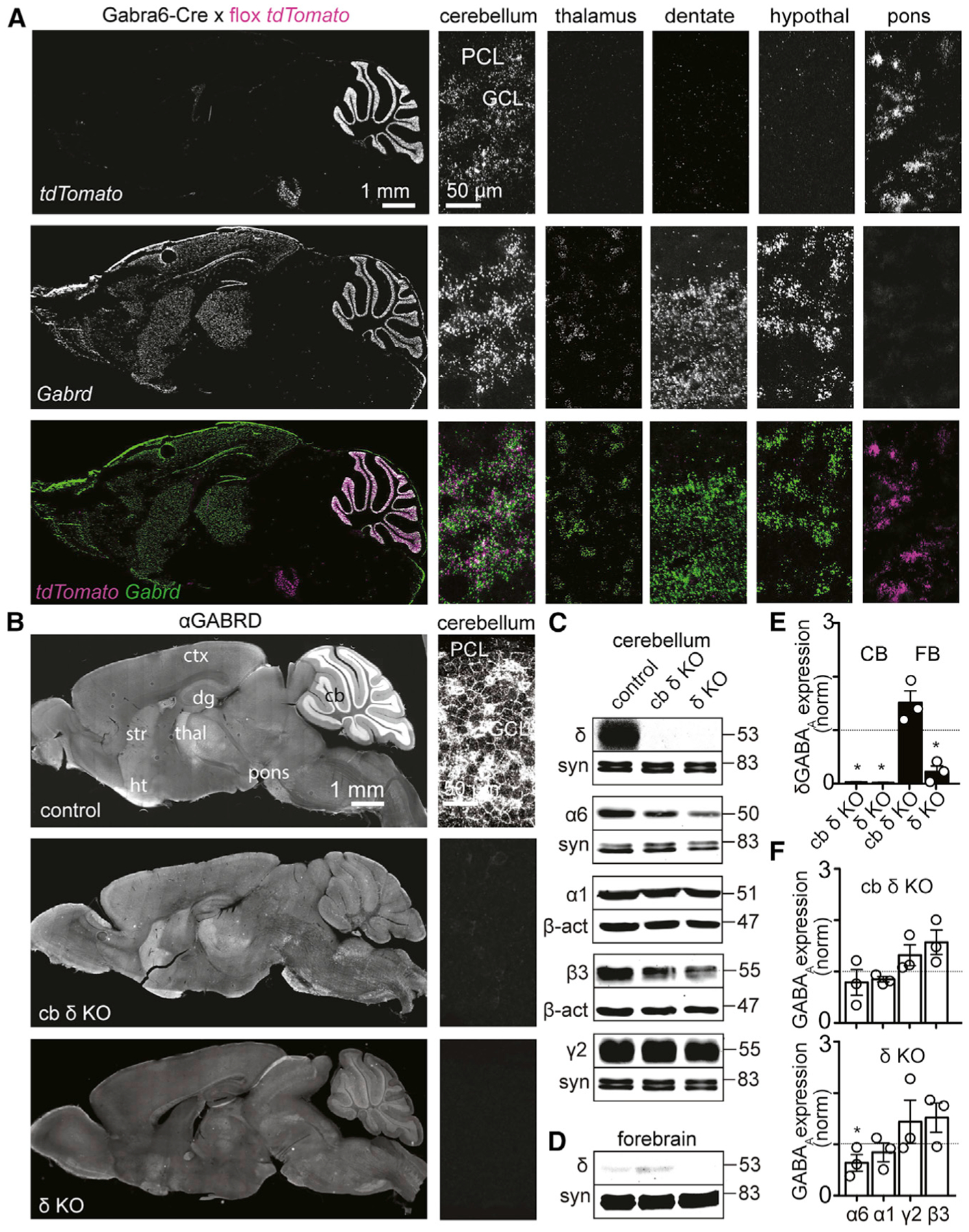
Specific Deletion of the δGABA_A_R in cb GCs (A) FISH labeling of *tdTomato* transcripts in the Gabra6-Cre mouse line crossed to the Ai14 reporter line (top), *Gabrd* transcripts (center, *Gabrd*), and merged images (bottom). Left: sagittal section of the whole brain. Right: confocal images of the cerebellum, thalamus, dentate gyrus, hypothalamus, and pons. *tdTomato* and *Gabrd* transcripts are co-expressed exclusively in cb GCs. (B) Immunostaining against δGABA_A_ in sagittal sections of the brain (left) and confocal images of the cerebellum (right) in control (top), cb δ KO (center), and global δ KO tissue (bottom). (C) Western blot of the cerebellum against the δGABA_A_, α6GABA_A_, α1GABA_A_, β3GABA_A_, and γ2GABA_A_ subunits; loading controls were synapsin (syn) or β-actin (bottom lanes). (D) Western blot of the forebrain against δGABA_A_R. (E) Quantification of δGABA_A_ expression levels in the cerebellum (white bars) and forebrain (black bars) in cb δ KO (left) and global δ KO mice. δGABA_A_ expression is abolished in the cerebellum of cb δ KO and δ KO mice and in the forebrain of δ KO mice but not in the forebrain of cb δ KO mice (one-sample t test of samples normalized to control, n = 3 animals, p < 0.05). Error bars represent SEM. (F) Quantification of α6GABA_A_, α1GABA_A_, β3GABA_A_, and γ2GABA_A_ expression levels in the cerebellum (white bars) of cb δ KO mice (center) and global δ KO mice (bottom). α6GABA_A_ expression was decreased significantly in the cerebellum of δ KO mice (p < 0.05). All other receptors did not show significant changes in expression levels (one-sample t test of samples normalized to control, n = 3 animals, p > 0.05). Error bars represent SEM.

We first determined the overlap of Cre expression in the Gabra6-Cre mouse line and δGABA_A_ using fluorescence *in situ* hybridization (FISH) in whole-brain sagittal sections of Gabra6-Cre × flox *tdTomato* mice (Ai14 reporter line; Figure 1A, left panels). FISH-labeled *tdTomato* RNA indicates Cre expression in the GC layer and in the pons (Figure 1A, top), consistent with earlier descriptions (Fünfschilling and Reichardt, 2002). In addition, *TdTomato* transcripts were absent in the thalamus, dentate gyrus, and hypothalamus (Figure 1A, top). *Gabrd* was expressed in the neocortex, striatum, dentate gyrus, thalamus, hypothalamus, and cerebellum but not in the pons (Figure 1A, center; Figure S1). *TdTomato* and *Gabrd* FISH signals did not overlap in other regions of the hippocampus, colliculus, and brain stem nuclei (Figure S1A). Previous reports show that GCs of cochlear nuclei lack *Gabrd* (Campos et al., 2001). Thus, Cre and δGABA_A_ expression overlap selectively in cb GCs (Figure 1A, merged image, bottom). To selectively eliminate δGABA_A_s from cb GCs (cb δ KO mice), we bred Gabra6-Cre to floxed *Gabrd* mice (Lee and Maguire, 2013) (Gabra6-Cre+, *Gabrd* flox/flox).

Immunohistochemistry of parasagittal whole-brain sections of control mice (*Gabrd* flox/flox) revealed that GCs strongly express δGABA_A_ subunits (Figure 1B, top left). Confocal images of the cerebellar GC layer are consistent with GC membranes (Figure 1B, top right). However, in cb δ KO mice, the δGABA_A_ signal was abolished almost entirely in the cerebellum (Figure 1B, center). An image of the background fluorescence in global δ KO mice is shown for comparison (Figure 1B, bottom). A comparison of the images in Figure 1B suggests that, in cb δ KO mice, the δGABA_A_ subunit is eliminated from GCs but remains intact in other brain regions. Immunohistochemistry also indicates that δGABA_A_ expression does not overlap with tdTomato in a sparsely labeled population of hippocampal interneurons and in brain stem nuclei (Figures S1B and S1C). Fluorescent western blot analysis revealed that δGABA_A_ is expressed at high levels in the cerebellum of control mice but eliminated from cb δ KO and global δ KO animals (Figures 1C, top panel, and 1E). δGABA_A_ is expressed at relatively low levels in the forebrain of control mice, which were reduced to even lower levels in δ KO animals, whereas levels were slightly elevated in cb δ KO mice (Figures 1D, top panel, and 1E). We conclude that, in Gabra6-Cre 3 *Gabrd* flox mice, δGABA_A_ subunits are eliminated selectively from cerebellar GCs.

Deletion of a GABA_A_R subunit can affect expression of other subunits and, therefore, the relative contributions of phasic and tonic inhibition (Korpi et al., 2002; Nusser et al., 1999; Ogris et al., 2006; Peng et al., 2014). In cerebellar GCs, tonic currents are mediated by extrasynaptic α6/δ-containing receptors, and phasic currents are mediated by α1/β3/γ2-containing subunits (Nusser et al., 1998). Besides a small reduction in α6 levels that is only significant in global δ KO mice, there were no significant differences in cb α1, β3, and γ2 subunit levels in control, cb δ KO, and global δ KO mice (Figures 1C and 1F), suggesting that phasic currents are likely not upregulated. We also found that spontaneous phasic inhibitory postsynaptic currents (spIPSCs) in GCs were similar in amplitude (control: 32 ± 5 pA, n = 7; cb δ KO: 26 ± 2 pA, n = 9; p > 0.2), rise time (control, 0.8 ± 0.1; cb δ KO, 1.1 ± 0.1 ms; p > 0.1), and decay time (control, 4.5 ± 0.8 ms; cb δ KO, 4.8 ± 0.4 ms; p > 0.4; Mann-Whitney test). This suggests that elimination of δGABA_A_ does not have a major influence on other GABA_A_R subunits in the cerebellum.

**Figure 2.**
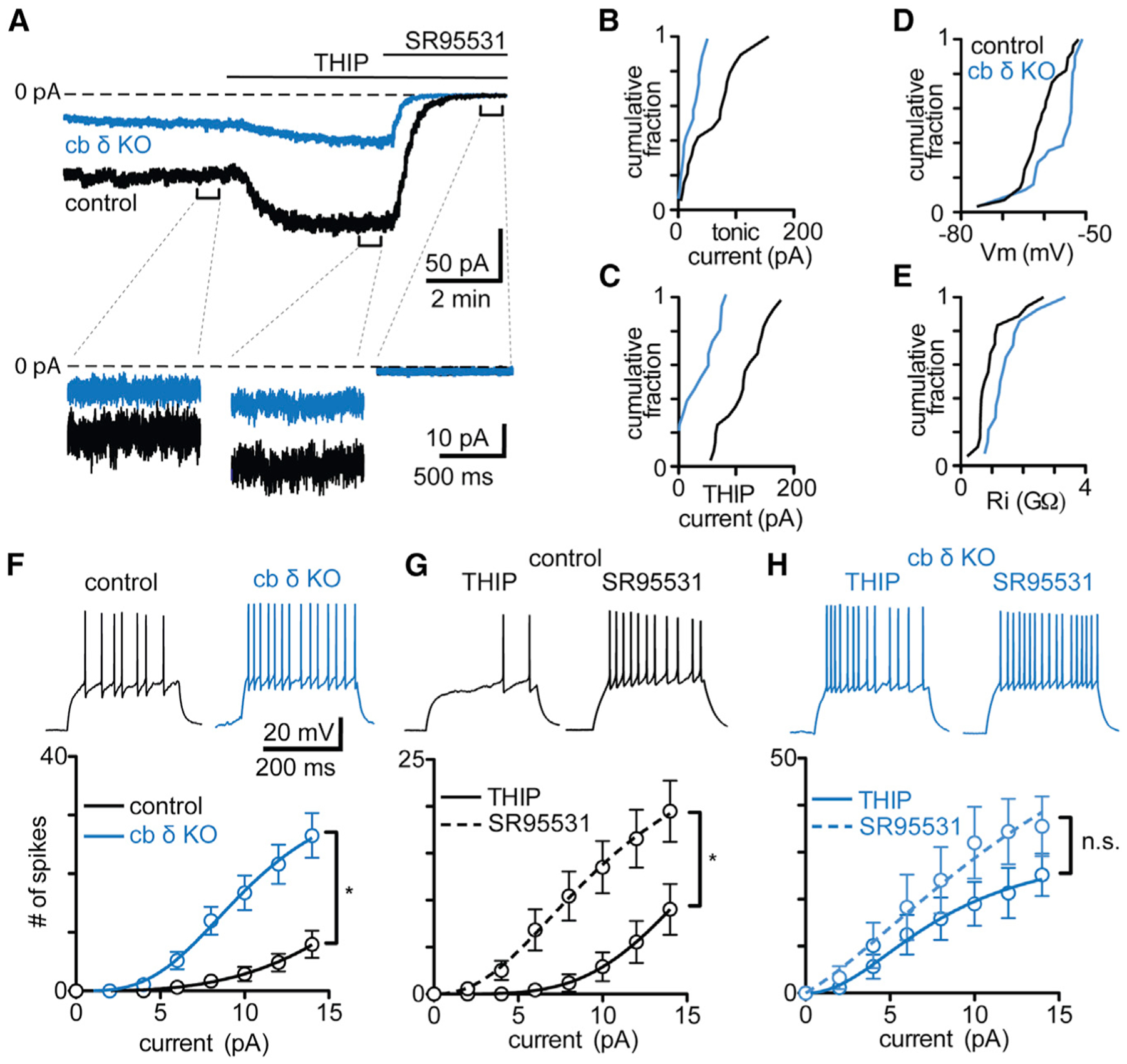
Hyperexcitability and Decreased Tonic Inhibition in GCs Lacking the GABA Receptor δ Subunit (A) Top: example traces of whole-cell voltage-clamp recordings of tonic currents measured during a baseline period, in the presence of the δGABA_A_ subunit containing-preferring GABA_A_R agonist THIP, and in the presence of the non-selective GABA_A_R antagonist SR95531 for a control (black) and a cb δ KO GC (blue). Bottom: current traces on an expanded timescale show varying noise levels depending on the recording conditions. SR95531 strongly reduces noise levels compared with the control and THIP. (B) Cumulative histograms of initial tonic currents in control (black line) and cb δ KO GCs (blue line). (C) Cumulative histograms in the presence of THIP. (D) Cumulative histograms of membrane potential (V_m_). (E) Cumulative histogram of input resistance (R_i_). (F) Top: example traces of whole-cell current clamp recordings in response to a 10-pA depolarizing current injection in cb δ KO GCs (blue) and in control GCs (black). Bottom: summary data showing the input-output relationships for a range of depolarizing current steps and the resulting number of action potentials. Error bars represent SEM. (G) As in (F) but for control animals in the presence of THIP and with GABA_A_Rs blocked. Error bars represent SEM. (H) As in (G) but for cb δ KO mice. Error bars represent SEM.

We assessed tonic currents and the passive properties in control and cb δ KO GCs using patch-clamp recordings in cerebellar brain slices (Figures 2A–2E). Initial tonic currents were larger in control compared with cb δ KO GCs (Figures 2A and 2B; control: 74 ± 10 pA, n = 23 cells; cb δ KO: 21 ± 4 pA, n = 14 cells; p < 0.0005, Kolmogorov-Smirnov [KS] test). Tonic currents were accompanied by a characteristic increase in GABA_A_R channel noise (Figure 2A, insets; Figure S2F). 4,5,6,7-Tetrahydroisoxazolo (5,4-*c*)pyridin-3(-ol) (THIP), a δGABA_A_-preferring GABA_A_R agonist (Chandra et al., 2006; Meera et al., 2011; Stórustovu and Ebert, 2006), increased current amplitude to a larger extent in control than in cb δ KO GCs (Figure 2C; control: 116 ± 8 pA, n = 23 cells; cb δ KO: 38 ± 8 pA n = 14 cells; p < 0.0001, KS test). The remaining effect of THIP in cb δ KOs is consistent with activation of non-δGABA_A_-containing receptors (Meera et al., 2011). Blocking GABA_A_Rs eliminated tonic current and greatly reduced noise (Figures 2A–2C, black). A small but significant depolarization was evident in cb δ KO GCs (Figure 2D; control: −61 ± 1 mV, n = 28; cb δ KO: −57 ± 1 mV, n = 31; p < 0.05, KS test; Figures S2A–S2C). The input resistance (R_i_) was higher in cb δ KO GCs compared with the control (Figure 2E; control: 1.0 ± 0.1 GΩ, n = 18 cells; cb δ KO: 1.5 ± 0.2 GΩ, n = 14 cells; p < 0.02, KS test), consistent with reduced tonic current. Additionally, in control animals, THIP decreased R_i_, whereas blocking GABA_A_Rs increased R_i_ (Figure S2D), but THIP and SR95531 did not alter R_i_ in cb δ KO GCs (Figure S2E). Thus, decreased tonic inhibition in cb δ KO GCs results in increased R_i_. Tonic current amplitudes were larger in GCs of control females than of males (Figure S2G), whereas THIP evoked currents were of similar amplitude (Figure S2H), suggesting that, although δGABA_A_R density is similar in males and females, baseline activation differs.

Tonic currents regulate GC excitability, but it is unclear whether deletion of δGABA_A_ leads to hyperexcitability or whether compensatory mechanisms counteract the loss of tonic inhibition (Brickley et al., 2001). We found that depolarizing current steps evoked more action potentials in cb δ KO GCs than in control GCs (Figure 2F, top). This was true for a range of amplitudes (Figure 2F, bottom; control, n = 14; cb δ KO, n = 14; p < 0.0001, 2-way ANOVA), indicating that cb δ KO GCs are more excitable than control GCs. The observation that THIP decreased excitability (Figure 2G, top left black trace), whereas inhibiting GABA_A_Rs increased excitability of control GCs (Figure 2G, top right black trace; bottom, summary input-output curve; control THIP, n = 19; control SR95531, n = 17; p < 0.0004, 2-way ANOVA; Figure 2G) confirmed the importance of tonic GABA_A_ currents in regulating GC excitability. Conversely, neither activating nor inhibiting GABA_A_Rs had a significant effect on excitability in cb δ KO GCs (Figure 2 H; THIP, n = 17; SR95531, n = 15; p > 0.9, 2-way ANOVA). Tonic current amplitudes were similar across lobules (Figure S2I); thus, hyperexcitability of cb δ KO GCs affects all areas of the cb cortex. These experiments indicate that GCs of cb δ KO mice are hyperexcitable because of reduced tonic inhibition.

GC hyperexcitability in cb δ KO mice was surprising because eliminating α6GABA_A_ did not result in hyperexcitability (Brickley et al., 2001). This might be related to the fact that α6GABA_A_ receptors are still present in GCs after GC-specific δGABA_A_ deletion (Martenson et al., 2017), whereas global α6GABA_A_ resulted in a strong reduction of δGABA_A_. Thus, cb δ KO mice provide a unique opportunity to assess the behavioral consequences of input layer hyperexcitability in the cb cortex.

**Figure 3.**
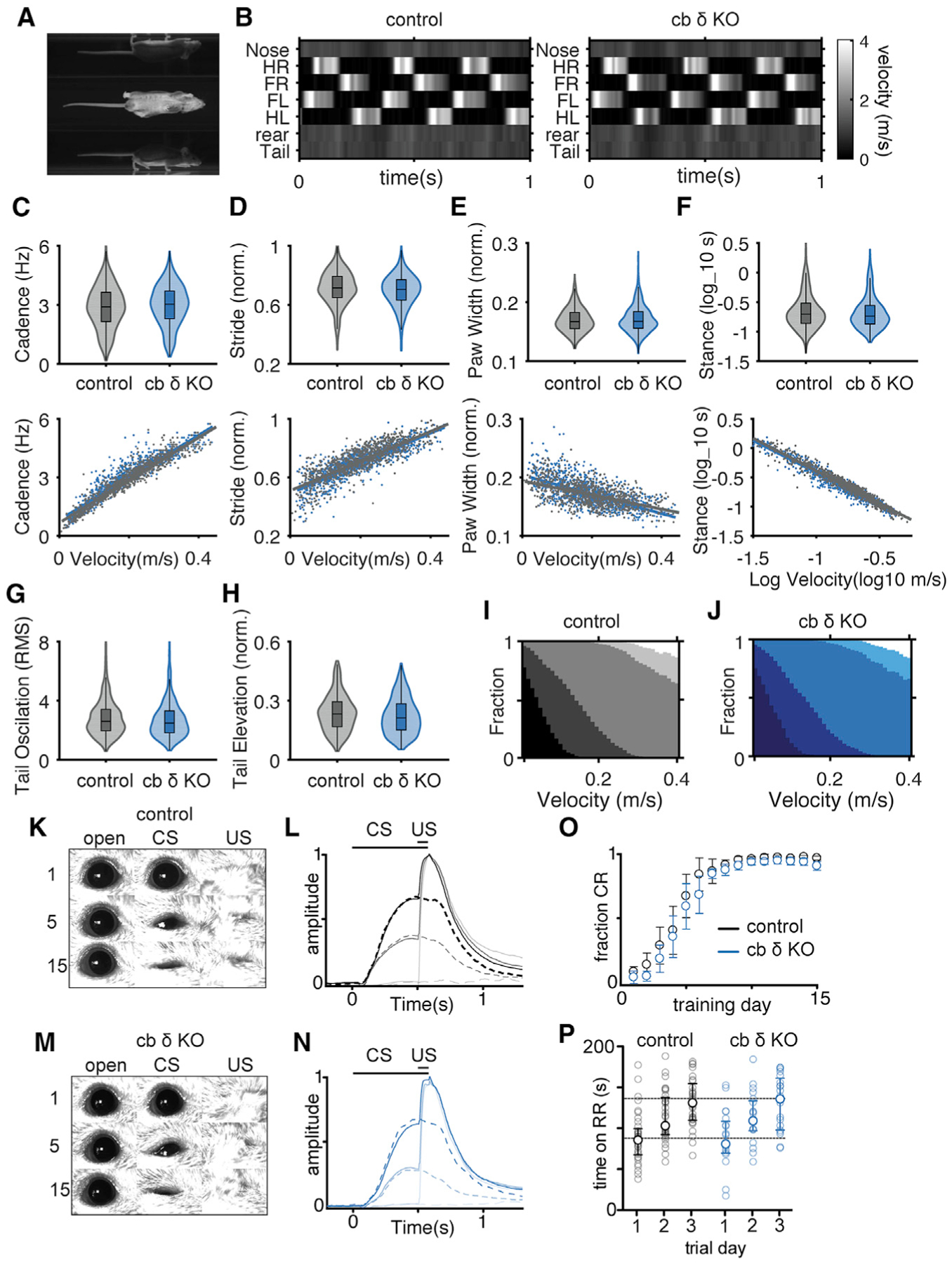
Normal Motor Function and cb Learning in Cerebellar δ KO Mice (A) Sample frame of a walking mouse viewed from the left, right, and bottom. (B) Velocity of different body parts over time in control and cb δ KO mice. (C–F) Top: box-and-whisker plots overlaid on the density plots for cadence (C), stride (D), paw width (E), stance (F), tail oscillation (G), and tail elevation (H), measured stride to stride and pooled across all subjects, with control shown in gray and cb δ KO in blue. Bottom: scatterplots and linear regression of cadence, stride, paw width, and stance against velocity. (I and J) Fraction of time during a single gait cycle for a given velocity that had four (darkest) to zero (white) supports on the floor. (K) Representative images of eyeblink responses in a control animal sampled at the beginning (day 1, top), in the middle (day 5, center), and at the end of training (day 15, bottom). The baseline, conditioned response (CR) and air puff response (UR) are presented in sequence column-wise. Initially (day 1), the eye remains fully open during presentation of the CS and closes fully only with presentation of the US. With continued learning (day 5), the mouse begins to close its eye in response to the CS until near-full closure at the end of the training period (day 15). The US always elicits complete closure of the eye. (L) Average normalized eye closure from early (day 1, light gray), middle (day 5, gray), and end of training (day 15, dark gray). t = 0 marks the presentation of the CS and 0.5 s presentation of the US. Solid lines represent paired presentation of CS and US at different learning stages, and dashed lines represent CS presentation only. (M and N) Analogous to (K) and (L) but in cb δ KO animals. (O) Fraction of trials with a CR on each training day. Cb δ KO animals (blue circles) learn at a similar rate as controls (white circles) in an eyeblink task (control, n = 8; cb δ KO, n = 8). Data are presented as mean and SEM. (P) Motor learning, as assayed in a 3-day accelerating rotarod paradigm, is similar in control (black) and cb δ KO animals (blue; control, n = 36 animals; cb δ KO, 20 animals; p > 0.9, Mann-Whitney test). Gray circles represent individual animals.

Cerebellar dysfunction is associated with ataxia and abnormal fine motor coordination (Manto et al., 2012). We therefore hypothesized that a considerable increase in excitability of the cerebellar input layer in cb δ KO mice could lead to motor dysfunction, including abnormal gait and deficits in motor learning. However, similar to observations with global δ KO mice (Wiltgen et al., 2005), we found that motor function was normal in cb δ KO mice. To examine whether δGABA_A_ deletion results in subtle effects on motor function, we analyzed the gait of cb δ KO mice. We tracked walking mice and extracted the positions of the four paws, the base and tip of the tail, and the nose over time (see representative frames of the recorded video in Figure 3A). A velocity plot of different mouse body parts illustrates that control and cb δ KO mice move similarly. Nose and tail advance at near-constant velocity, and the hind and front paws move together and touch the floor or are moving forward rapidly. (Figure 3B). There was no difference in any parameter describing gait (Figures 3C–3F, top). As expected, these parameters depend on velocity (Figures 3C–3F, bottom), and the velocity dependence of any of these parameters was the same in control and cb δ KO animals. We also found that tail oscillations that accompany walking and tail elevation were unaltered in cb δ KO mice (Figures 3G and 3H). Finally, we analyzed the fraction of the cycle in which there are a given number of paws on the floor. At low velocities, it is common that four paws are in contact with the floor. As velocity increases, progressively fewer paws contact the floor, to the point where no paws are in contact with the floor (Figures 3I and 3J). Unlike mice with gait abnormalities that tend to have more feet in contact with the floor at a given velocity (Machado et al., 2015), there was no difference between control and cb δ KO mice.

The cerebellum has a well-established role in motor learning, and eyeblink conditioning is a learning paradigm that requires the cerebellum (Thompson, 1986). Pairing a conditioning stimulus (CS, a weak illumination) that does not initially cause the eye to close with an unconditioned stimulus (US, air puff to the eye) that causes the eye to close by the fifth day of pairing resulted in the CS alone causing the eyelid to partially close in a fraction of trials and by the fifteenth day in eye closure in most trials. Delay conditioning experiments were performed in mice running on a rotating platform as the CS and US were presented (Albergaria et al., 2018). Cb δ KO mice learn similarly as control animals (control, Figures 3K and 3L; cb δ KO, Figures 3M and 3N). All control mice and cb δ KO mice learned to respond to the CS, and control and cb δ KO mice learned at approximately the same rate (Figure 3O; control, n = 8; cb δ KO, n = 8). Average responses to the CS alone and to paired CS + US stimulation were very similar for control and cb δ KO mice on days 5 and 15 (Figures 3L and 3N). Because cb deficits can affect motor learning (Kloth et al., 2015; Tsai, 2016), we also performed a 3-day accelerating rotarod paradigm. We found that there was no difference between control mice and cb δ KO mice in initial performance (control: 90 ± 5 s on rotarod, n = 36; cb δ KO: 89 ± 8 s on rotarod, n = 20; p > 0.9, Mann-Whitney test; Figure 3P) in females and males (Figure S3C), and motor learning was similar in control and cb δ KO animals by the end of training (Figure 3P; control: 136 ± 6 s on rotarod, n = 36; cb δ KO: 133 ± 7 s on rotarod, n = 20; p > 0.9, Mann-Whitney test) in males and females (Figures S3D and S3E). Thus, remarkably, despite hyperexcitability of the cb input layer in cb δ KO mice, we did not detect any abnormalities in motor function.

### Anxiety-like Behaviors in cb δ KO Mice

**Figure 4.**
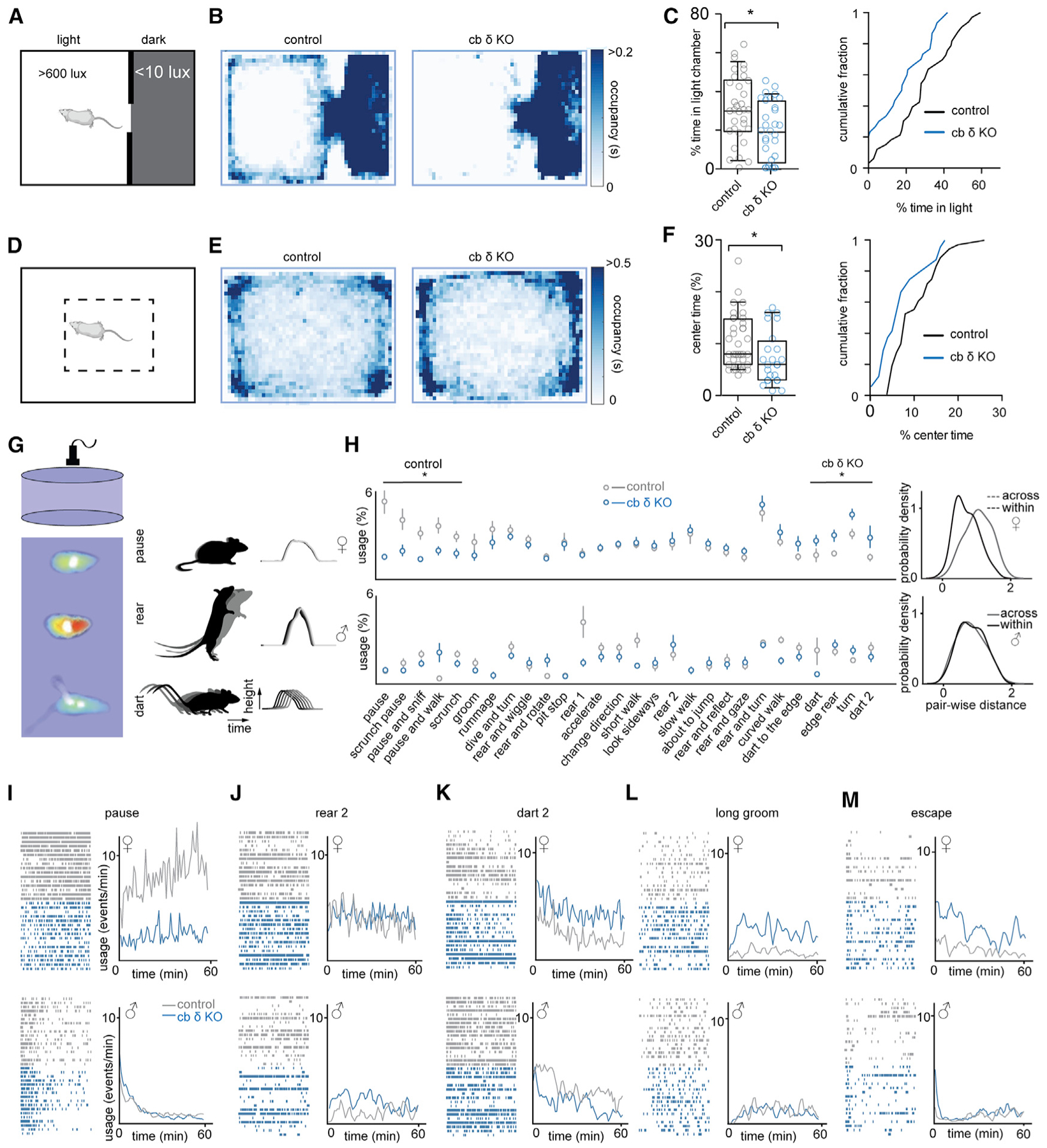
Anxiety-like Behavior and Sex-Specific Behavioral Abnormalities in cb δ KO Mice (A) Schematic of the light/dark chamber. Mice are placed in a behavioral arena divided into a dark compartment (<10 lux) and a lit compartment (>600 lux). A door connects the two chambers, allowing navigation between both compartments. (B) Median occupancy plots indicate that cb δ KO animals (right) spend less time in the lit compartment than control animals (left). (C) Summary data of the percentage of time spent in the light compartment. Left: boxes denote interquartile range and median; whiskers represent 10–90 percentile. Circles show individual control (n = 32, gray circles) and cb δ KO (n = 27, blue circles) animals (p < 0.009, Mann-Whitney test). Right: cumulative fraction of time spent in the light compartment (control, black line; cb δ KO, blue line). (D) Schematic of the open field behavior arena. A dashed rectangle denotes the center of the area. (E) Median occupancy plots of control (left) and cb δ KO animals (right). (F) Left: summary data of the percentage of time spent in the center of the behavioral arena. Circles show individual control (n = 36, gray circles) and cb δ KO (n = 24, blue circles) animals (p < 0.02, Mann-Whitney test). Right: cumulative probability graph of center time. Boxes denote interquartile range and median; whiskers represent 10–90 percentile. (G) 3D imaging of mice in the open field with a depth camera, followed by MoSeq-based segmentation of the behavior data, reveals behavioral syllables (STAR Methods); example behavioral syllables and the associated inferred positions of the spine in time and space are also shown (center and right). (H) Left: usage plot for the most frequently used syllables in females (top). A subset of behavioral syllables is differentially expressed in control and cb δ KO females. Male syllables were matched to corresponding female syllables (bottom). Control and cb δ KO males showed fewer differences in behavior. Right: pairwise distance comparison across control and cb δ KO syllables and within control and cb δ KO syllables reveals greater distance across control and cb δ KO syllables in females and similar distances across and within male syllables. (I) Example syllables performed more frequently by control females. Top left: raster plot of syllable occurrence during the observation period. Right, syllable usage over time. The observed behavior associated with each syllable is indicated. Bottom: raster plot and usage over time of matching male syllables. (J) Like (H) but depicting common example syllables that are equally prevalent in control and cb KO mice. (K) Like (H) but depicting common example syllables performed more frequently by cb KO females. (L and M) Like (H) but depicting rarely used syllables that are more frequently used by cb KO females.

δGABA_A_Rs are involved in anxiety-related behaviors and stress (Gunn et al., 2011). The behavioral responses to stress are diverse and can include hypervigilance and changes in locomotion (Cabib et al., 1988; Sequeira-Cordero et al., 2019). Open field testing revealed hyperlocomotion in cb δ KO mice (Figures S3A and S3B), which could indicate increased stress levels. To examine the effect of δGABA_A_ deletion in the cerebellum on anxiety-related behaviors, we first conducted a light/dark test that has been used previously to test the effect of anxiolytic drugs in mice (Crawley, 1985; Lezak et al., 2017). During the test, the mouse was allowed to navigate a dark and a brightly lit compartment (Figure 4A). A mouse displaying increased anxiety-like behavior is thought to elevate avoidance of the bright compartment. Cb δ KO mice spent less time in the bright compartment than control mice, as shown in median heatmaps (Figure 4B) and in the summaries of individual animals tested (Figure 4C; control: 34% ± 3%, n = 32; cb δ KO mice: 18% ± 3%, n = 27; p < 0.0008, Mann-Whitney test). Both males and females displayed increased anxiety-like behavior (Figure S4). In addition, a higher fraction of cb δ KO mice exclusively remained in the dark compartment during the observation period compared with control animals (6 of 27 and 1 of 32, respectively).

We also observed males and females in the open field. Decreased time spent in the center region of the enclosure is thought to indicate anxiety-like behavior (Figure 4D). Median occupation heatmaps (Figure 4E) and summaries of individual animals (Figure 4F) show that cb δ KO mice spent less time than cb δ KOs in the center of the arena (cb δ KO: 7.3% ± 1%, n = 30; control: 10.3% ± 1%, n = 41; p < 0.036, Mann-Whitney test). Interestingly, only cb δ KO females spent less time in the center (control: 10.7% ± 0.9%, n = 32; cb δ KO: 7.7% ± 1.1%, n = 21; p < 0.032; Figure S4E) but not males (control: 8.0% ± 1.1%, n = 9; cb δ KO: 7.6% ± 1.6%, n = 9; p > 0.9; Figure S4F).The light/dark and open field tests suggest that especially female cb δ KO mice display increased anxiety-like behavior compared with control mice.

### Sex-Specific Differences in Spontaneous Behavior in cb δ KO Mice Identified with MoSeq

We further used Motion Sequencing (MoSeq) (Wiltschko et al., 2015) to test whether cb δ KO mice show abnormalities in spontaneous behavior that are difficult to detect with alternative approaches. MoSeq posits that spontaneous behavior consists of discrete and stereotyped three-dimensional motifs of behavior called “syllables” and allows identification and quantification of these syllables using unsupervised machine learning. MoSeq can detect subtle behavioral differences caused by genetic mutations in mouse models of disease by presentation of sensory cues and in response to optogenetic manipulation (Markowitz et al., 2018; Pisanello et al., 2017; Wiltschko et al., 2015).

Single mice of either sex were observed in a circular behavioral arena with a depth camera (Figure 4G, left). MoSeq identified over 70 distinct syllables (Figures S5 and S6). Three examples are shown in Figure 4G, right. A comparison of the most common syllables (>1% usage) identified a subset of syllables whose usage differed between control and cb δ KO females but not males (Figure 4H). Although many syllables were used equally in control and cb δ KO females (Figures 4H and 4J; control, n = 8; cb δ KO, n = 8 females), pausing syllables were used less in cb δ KO females (Figures 4H and 4I; control, n = 8; cb δ KO, n = 8 males). Conversely, usage of syllables corresponding to rapid movements, such as darting or turning, were elevated in cb δ KO females (Figures 4H and 4K). MoSeq also identified less common behaviors that were more frequent in cb δ KO females, including long bouts of grooming (Figure 4L) and high wall jumps, presumably to attempt escape (Figure 4M). These syllables were not differentially expressed in control and cb δ KO males. Thus, although the syllabic repertoire of control and cb δ KO animals is similar, syllable usage patterns differ between the genotypes, especially in females (Figure 4H, right). The behaviors observed in cb δ KO females are consistent with maladaptation to stress, such as removal from the home cage and placement in a behavioral arena, and are reminiscent of complex behaviors displayed after chronic stress (Cabib et al., 1988; Füzesi et al., 2016). This observation is particularly interesting because the δGABA_A_ subunit has been implicated in these behaviors (Gunn et al., 2011; Liu et al., 2017; Maguire et al., 2005; Shen et al., 2007; Zhang et al., 2017b), but the possibility that δGABA_A_ in the cerebellum could contribute to anxiety-like and stress-related behaviors has not been demonstrated. The apparent milder phenotype observed in cb δ KO males could be related to well-established sex differences in the stress response and circulating sex steroids, both of which modulate δGABA_A_Rs (Bangasser and Valentino, 2012; Goldstein et al., 2010; MacKenzie and Maguire, 2013; Tolin and Foa, 2006).

### Decreased Sociability and Abnormal Maternal Behavior in cb δ KO Females

**Figure 5.**
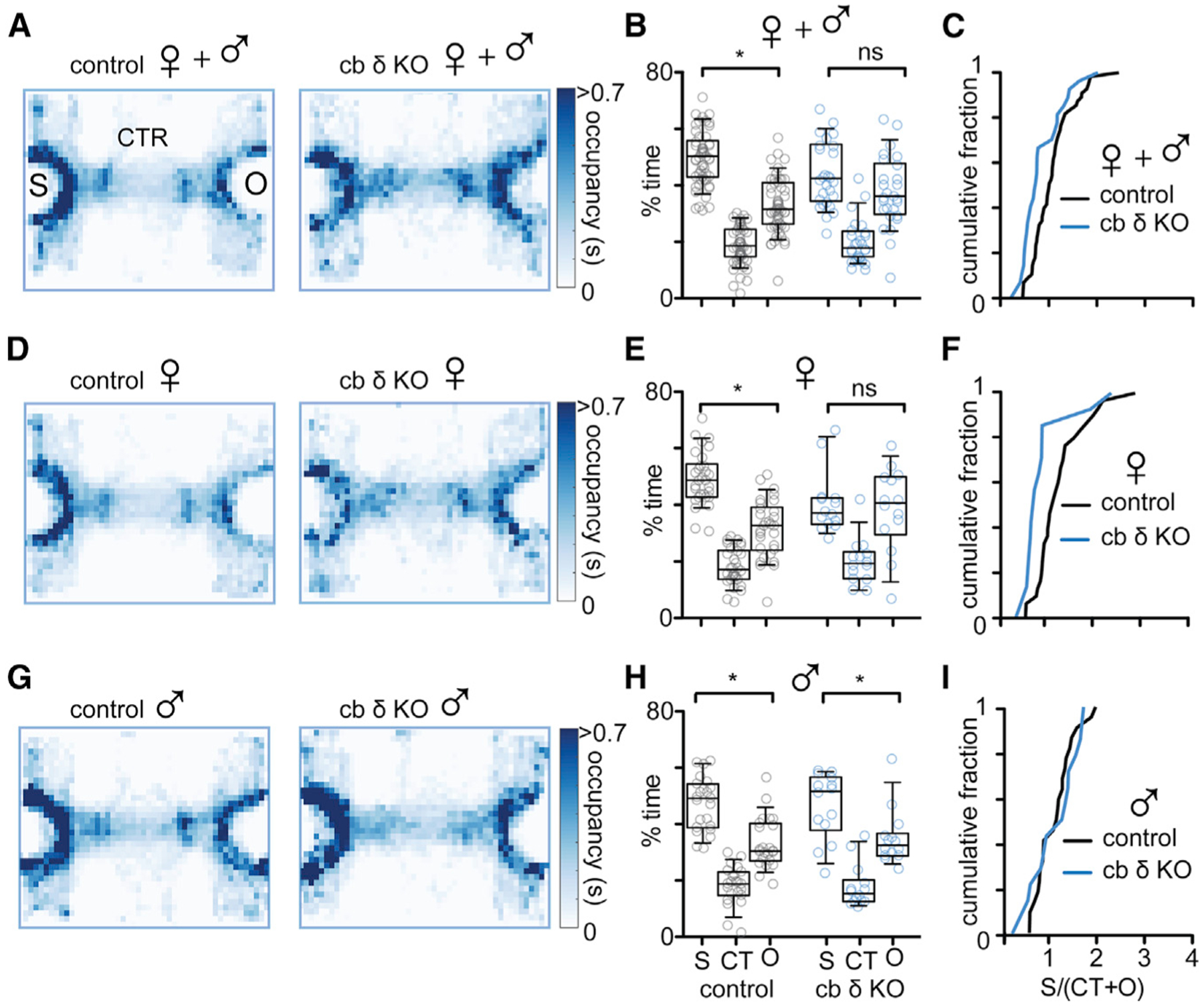
Sexually Dimorphic Effect of Cerebellum-Specific δGABA_A_ Deletion on Social Behavior (A) Median occupancy plots indicate a clear preference of control (left) animals for the S over the O, whereas cb δ KO (right) animals show no preference for either stimulus. (B) Summary data for the percentage of time spent in the O, center, and S compartment of the behavior chamber during the observation period. On average, control animals display a strong preference for the S (gray circles, p < 0.0001), whereas cb δ KO animals show no preference (blue circles). Boxes denote interquartile range and median; whiskers represent 10–90 percentile. (C) Cumulative probability plot of the ratio of time spent with the S and the sum of the time spent in the center (CTR) and O chambers. Sociability ratio S/(O+C); control, black trace; cb δ KO, blue trace. (D–F) Same as (A)–(D) but females only. (D) Median occupancy plots of control (left) and cb δ KO females (right). Control females show a strong preference for S (left), whereas cb δ KO (right) females do not show a preference for either stimulus. (E) Summary data of the percentage of time spent in the S, CTR, and O compartments. Boxes denote interquartile range and median; whiskers represent 10–90 percentile. (F) Cumulative probability plot of sociability ratios in females. Boxes denote interquartile range and median; whiskers represent 10–90 percentile. (G)–(I) Same as (A)–(D) but males only. (G) Median occupancy plots of control (left) and cb δ KO males (right). Control and cb δ KO males prefer S over the O. (H) Summary data of the percentage of time spent in the S, CTR, and O compartments. (I) Cumulative probability plot of the sociability ratios in males.

Our findings suggest that loss of δGABA_A_ in cerebellar GCs contributes to stress-related behaviors. Global loss of δGABA_A_ function is associated with increased fear-related behavior and stress sensitivity (Maguire and Mody, 2007; Mody and Maguire, 2012; Wiltgen et al., 2005). Stress has been linked to diminished interest in social interactions (Beery and Kaufer, 2015; File and Hyde, 1978), and anxiolytic agents acting on δGABA_A_ have prosocial effects (File, 1980). In addition, cerebellar dysfunction can lead to social deficits in humans and in animal models (Badura et al., 2018; Carta et al., 2019; Schmahmann and Sherman, 1998; Tsai, 2016), and decreased tonic inhibition with the cb input layer is evident in several mouse models of psychiatric disorders (Bruinsma et al., 2015; Kim et al., 2017). We therefore examined whether loss of δGABA_A_ in the input layer affects social behavior. We used a 3-chamber paradigm where mice were allowed to explore a chamber containing a sex-matched juvenile conspecific (social stimulus [S]) and a chamber containing a novel object (O). Median occupation heatmaps indicate that control mice preferred investigating the S to the O, whereas cb δ KO mice show no preference for either stimulus (Figure 5A; control, left; cb δ KO, right). Summary data of individual animals are shown in Figures 5B and 5C (control: 32% ± 1% in O, 50% ± 1% in S, n = 54, p < 0.0001; cb δ KO: 37% ± 2% in O, 44% ± 2% in S, n = 29, p > 0.1; Mann-Whitney test). Control and cb δ KO animals did not prefer either chamber in the absence of stimuli (Figures S7A–S7C). Control animals also investigated the conspecific for a longer duration than cb δ KO mice (Figure S7D; control: 133 ± 9 s, n = 54; cb δ KO: 108 ± 9 s, n = 29; p < 0.04, Mann-Whitney test). The number of entries to the O or social chambers were similar (Figure S7E), suggesting that control and cb δ KO mice did not avoid either chamber and showed normal exploratory behavior. However, social deficits were pronounced in females and absent in males. Although control females strongly preferred the social over the O chamber, cb δ KO mice had no preference (Figure 5D; summary in Figures 5E and 5F; control: 32% ± 2% in O, 50% ± 2% in S, n = 29, p < 0.0001; cb δ KO: 39% ± 4% in O, 41% ± 3% in S, n = 15, p > 0.8). In contrast, there was no difference in social interest between control and cb δ KO (Figures 5G–5I; control: 33% ± 2% in O, 48% ± 2% in S, n = 25, p < 0.002; cb δ KO: 34% ± 3% in O, 48% ± 3% in S, n = 14, p < 0.04; Mann-Whitney test). Thus, deletion of cb δGABA_A_ subunits in GCs leads to social deficits in females but not in males.

We tested whether the lack of social interest in cb δ KO mice was due to an olfactory deficit by performing an odor discrimination task. We used non-social (water, coconut, raspberry, and banana) and social (male and female urine) odors. Control and cb δ KO mice explored most odors for similar durations (Figure S7F). No major differences were observed between the sexes (Figure S7G). These experiments indicate that social deficits in cb δ KO mice are not a consequence of an inability to detect social odors.

**Figure 6.**
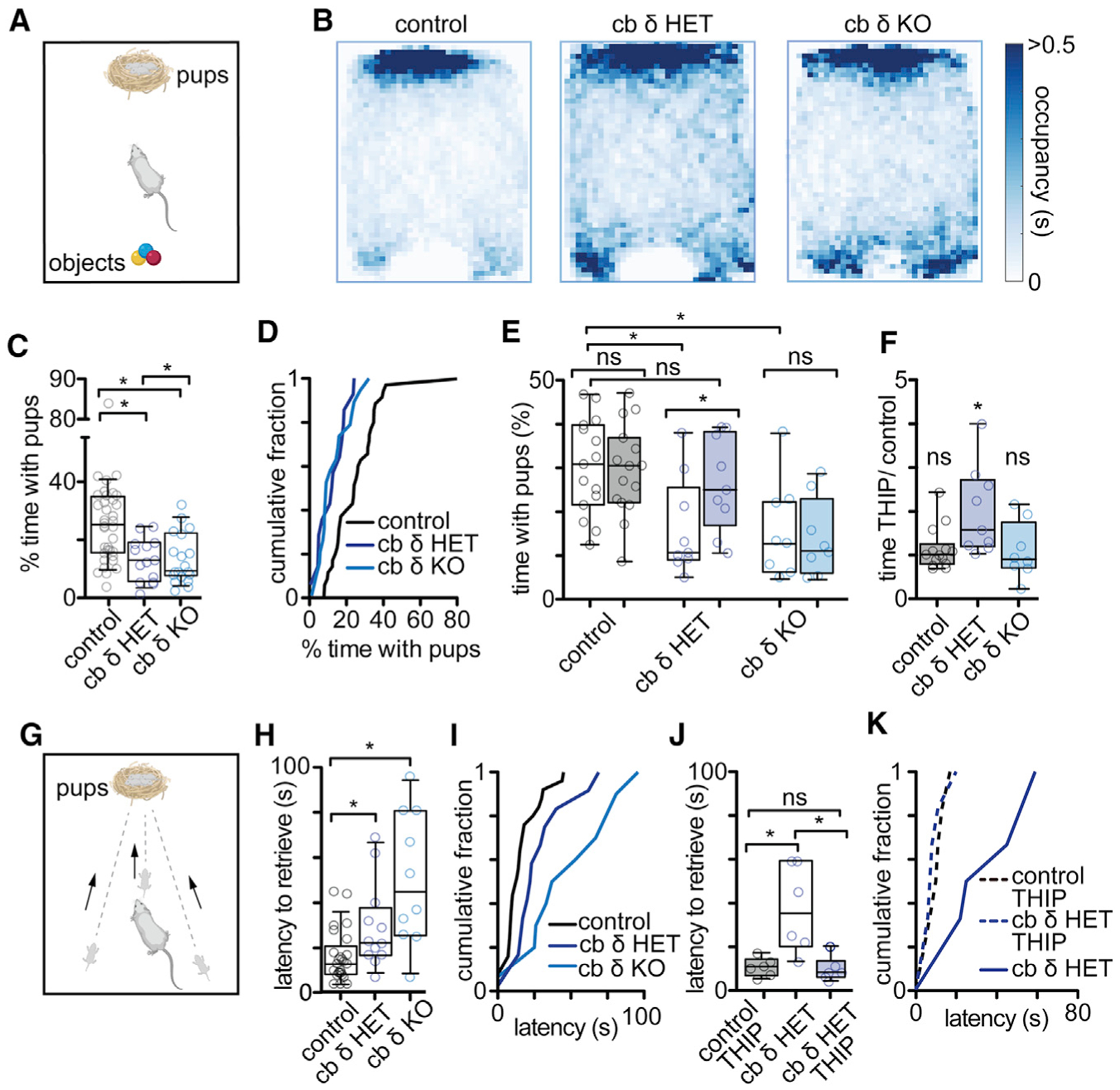
Altered Maternal Behavior in cb δ KO Females (A) Schematic of the behavioral paradigm. A virgin female is placed in a behavioral arena that contains a nest with three pups (top) and three pup-sized novel Os (bottom). (B) Median occupancy plots of control, cb δ HET, and cb δ KO females. Control females preferentially remain in close proximity of the nest, whereas cb δ HET and cb δ KO mice disperse around the nest and explore Os. (C) Summary plot of time spent with pups during the observation period. Circles show individual mice tested (control: gray, n = 37; cb δ KO het: dark blue, n = 12; cb δ KO: blue, n = 19; Kruskal-Wallis test with Dunn’s post-test, p < 0.003). Boxes denote interquartile range and median; whiskers represent 10– 90 percentile. (D) Cumulative probability plot of time spent with pups (p < 0.02 control compared with KS cb δ HET, p < 0.03 control compared with KS cb δ KO test, p > 0.9 cb δ HET compared with cb δ KO). (E) Pharmacological rescue of behavior in cb δ HET virgin females. The time animals spent with pups was measured under control conditions and after administration of THIP. THIP increased the time that cb δ HET females spent with pups but not had no effect on the time in cb δ KO and control females spent with pups, control and control THIP (gray circles, shaded gray, n= 15: p> 0.8)cbδHETandcbδHET THIP (dark blue circles, shaded dark blue, n = 9: p < 0.004), and cb δ KO and cb δ KO THIP (light blue circles, shaded light blue, n = 8: p > 0.6); Wilcoxon matched-pairs signed rank test. Boxes denote interquartile range and median; whiskers represent 10–90 percentile. (F) Ratio of time spent with pups before and during THIP administration, control (shaded gray, n = 15, p > 0.9), cb δ HET (shaded dark blue, n = 9, p < 0.004), and cb δ KO (shaded blue, n = 8, p > 0.9); Wilcoxon signed-rank test. (G) Schematic of the retrieval paradigm. In the home cage, 3 pups are removed from the nest and placed in the opposite 2 corners and in the CTR of the cage. (H) Summary plot of latency to retrieve the first pup. Circles show individual mice tested (control: gray, n = 25; cb δ KO het: dark blue, n = 12; cb δ KO: blue, n = 10; Kruskal-Wallis test with Dunn’s post-test, p < 0.008). Boxes denote interquartile range and median; whiskers represent 10–90 percentile. (I) Cumulative probability plot of retrieval times (KS test, p < 0.008). (J) Pharmacological rescue of retrieval latency in cb δ HET postpartum females. Boxes denote interquartile range and median; whiskers represent 10–90 percentile. (K) Cumulative probability plot of latency to retrieve in the presence and absence of THIP. Control (n = 6) and cb HET (n = 6), p < 0.02; cb HET and cb HET THIP (n = 6), p < 0.04; control and cb HET THIP, p > 0.9; KS test.

The neurosteroid sensitivity of δ subunit-containing GABA_A_Rs could contribute to the sex dependence of social deficits in cb δ KO mice. This raises the possibility that δGABA_A_-containing receptors in the cerebellum might also contribute to other steroid-sensitive behaviors in females, such as postpartum-related changes in maternal care (Maguire and Mody, 2008). Given the high δGABA_A_ expression levels in the cerebellum, social deficits specifically in cb δ KO females, and the established relationship between stress and poor maternal care (Hillerer et al., 2012), we next assayed parental behavior in cb δ KO females. We included heterozygous cb δ females (cb δ HET) because parental care is compromised in HET global δ KO mice (Maguire and Mody, 2008). Newborn pups emit olfactory signals and ultrasonic vocalizations that can stimulate spontaneous parental behavior in virgin females, including licking and crouching over pups (Calamandrei and Keverne, 1994; Gandelman, 1973; Noirot, 1969; Thomas and Palmiter, 1997). We thus first tested whether δGABA_A_ deletion in the cerebellum affects spontaneous parenting in virgin females. We developed a test where female virgins were allowed to interact with a nest of newborn (post-natal day 1 [P1]–P3) pups and inanimate pup-sized Os to control for novelty (Figure 6A). Median occupation heatmaps (Figure 6B) and summaries of individual experiments (Figures 6C and 6D; Figures S8A and S8B) show that, although control females show a preference for pups, cb δ HET and KO females were less interested and also interacted with Os. Upon habituation with pups, female virgins are known to retrieve displaced pups to the nest (Noirot, 1969). We found that control virgins retrieved pups at higher rates than cb δ KO and cb δ HET virgins (Figure S8C; control, 43%; cb δ HET, 29%; cb δ KO, 5%; p < 0.009, chi-square test). These results suggest that virgin cb δ KOs and cb δ HET females are less likely to express spontaneous parent-like behavior. To determine whether cb δGABA_A_Rs are involved in spontaneous parenting, we took advantage of the behavioral effects of δGABA_A_ deletion in cb δ HET animals. We administered the δ GABA_A_R-preferring agonist THIP via drinking water (STAR Methods) to control, cb δ HET, and cb δ KO females and assayed parental behavior. We found that THIP rescued behavior in cb δ HET females (Figures 6E and 6F; cb δ HET, 17% ± 4% time; cb δ HET THIP, 28% ± 3% time; n = 9, p < 0.004, Wilcoxon matched-pairs signed rank test; for time with Os, see Figures S8D and S8E), whereas it had no effect on controls (control, 30% ± 3% time; control THIP, 30% ± 3% time; n = 15, p > 0.8) and cb δ KO females (cb δ KO, 16% ± 4% time; cb δ KO THIP, 14% ± 3% time; n = 8, p > 0.6). THIP also decreased retrieval time in cb δ HETs (Figure S8F) but had no effect on baseline locomotion in cb δ HET and cb δ KO mice, indicating that THIP was provided at non-sedating concentrations (Figures S8G and S8H). These experiments establish that activation of remaining δ GABA_A_Rs in cb δ HET females can rapidly rescue parental behavior. The observation that δGABA_A_ levels in cb δ KO animals are not diminished in brain regions outside of the cerebellum (Figure 1D), together with the lack of effect of THIP on parental behavior in cb δ KO females, suggests that the behavioral effect of THIP is due to activation of cerebellar δGABA_A_Rs.

To test whether cerebellar δGABA_A_ deletion alters postpartum behavior, we first assayed a robust behavior expressed by postpartum mice: retrieval of pups to the nest (Figure 6H). We found that cb δ KO dams delayed initiation of retrieval compared with controls (control: 16 ± 2 s, n = 25; cb δ HET: 29 ± 5, n = 12; cb δ KO: 51 ± 9 s, n = 10; p < 0.008, Kruskal-Wallis test; Figures 6H and 6I). Cb δ KO dams also took longer to complete retrieval of three displaced pups (Figures S9A and S9B). Reproduction was normal in cb δ KOs (Figures S9C and S9D). However, similar to previous reports of global δ KO females (Maguire and Mody, 2008), we observed increased cannibalization of newborn pups in cb δ KO females (see pups of representative litters in Figures S9E and S9F). Contrary to global δ KO dams (Maguire and Mody, 2008), we found no differences in the Porsolt forced swim test (Figure S9G) or nest building (data not shown). Analogous to rescue of virgin behavior, we tested whether THIP could rescue parental behavior in cb δ HET dams. We found that THIP administration during the peripartum period (STAR Methods) reduced the retrieval time in cb δ HET dams (10 ± 2 s, n = 6) to similar durations as for control females (11 ± 2 s, n = 6), whereas simultaneously tested cb δ HET females that received regular drinking water (87 ± 12 s, n = 6; Figures 6J and 6K; Figures S9H and S9I) took longer to retrieve pups. Cannibalization of pups did not occur in THIP-treated cb δ HET dams (Figure S9J). These results suggest that the maternal behaviors disrupted in global δ KOs were recapitulated in cb δ KO and cb δ HET KO mice.

### Differential Activation of Many Brain Areas in cb δ KO Animals

**Figure 7.**
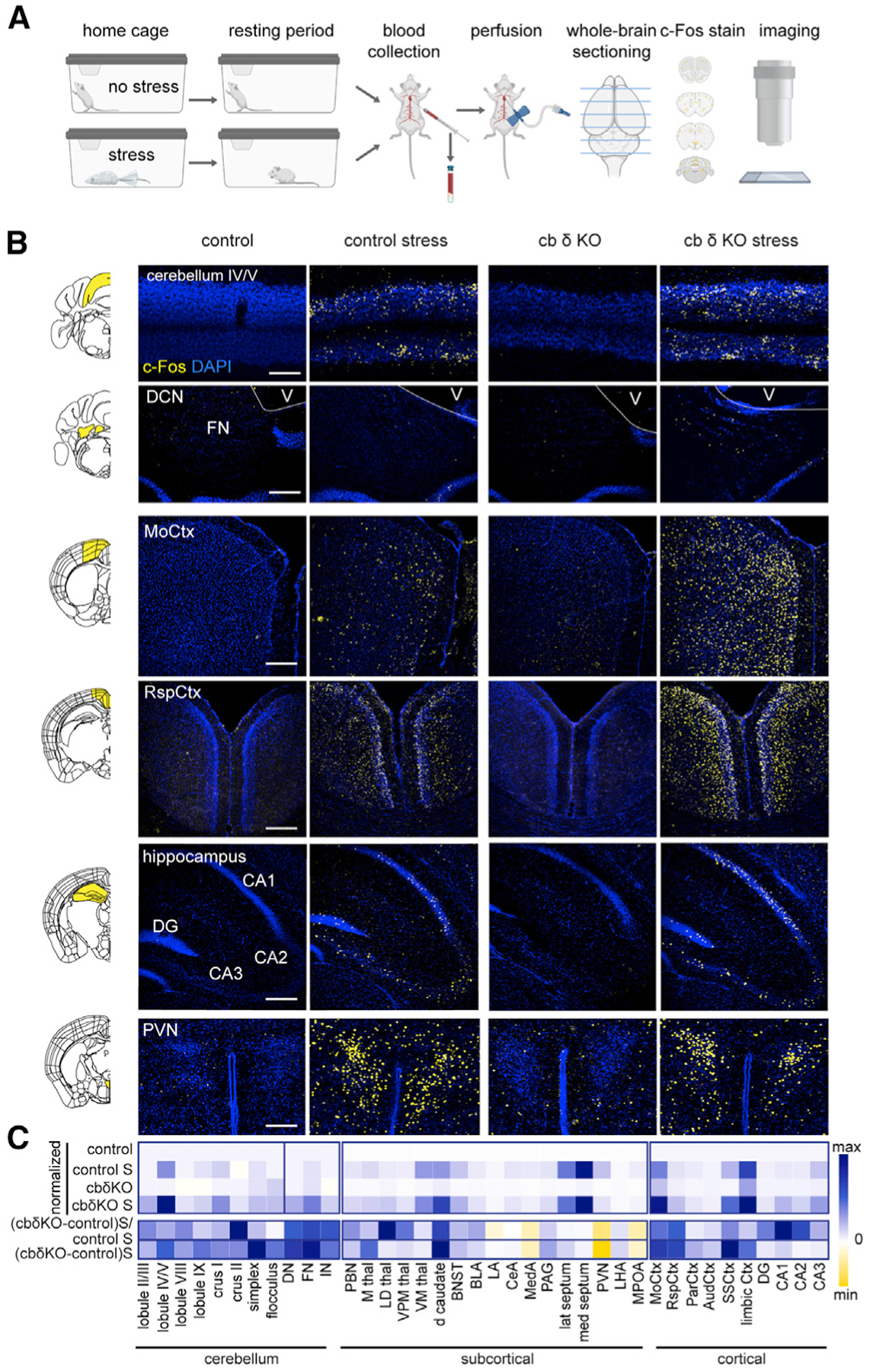
Differential Activation of Diverse Brain Regions in cb δ KO Animals in Response to Stress (A) Schematic of the experimental paradigm. Animals were subjected to 30 min of restraint stress or remained undisturbed in their home cage. After discontinuation of restraint, animals were allowed to recover for 60 min. Animals were then anesthetized, and trunk blood was taken from the left atrium of the heart before transcardial perfusion with a fixative. The whole brain was then sectioned, immunostained for c-Fos, and imaged for further analysis. (B) Left: reference atlas images of coronal brain sections (https://mouse.brain-map.org/static/atlas). The brain regions shown in the corresponding confocal images (right) are highlighted in yellow. Right: representative confocal images of unstressed and stressed control and cb δ KO animals (blue, DAPI; yellow, c-Fos). Example brain regions include the cb cortex (lobule IV/V), deep cb nuclei (DCN); fastigial nucleus [FN], fourth ventricle [V]), motor cortex (MoCtx), retrosplenial cortex (RspCtx), hippocampus (CA1, CA2, and CA3 regions), and the paraventricular nucleus (PVN) of the hypothalamus. (C) Top 4 panels: heatmap of normalized c-Fos expression. Acute restraint stress increased c-Fos expression in many cb, subcortical, and cortical brain areas. Bottom panel: difference and normalized difference heatmap of c-Fos expression in stressed cb δ KO animals and stressed controls.

To examine the consequences of GC hyperexcitability *in vivo*, we assessed neuronal activity at rest and after exposure to stress using whole-brain labeling of c-Fos, an immediate-early gene product that serves as an activity marker (Bullitt, 1990). Control and cb KO animals were left undisturbed or exposed to restraint stress (Figure 7A), a paradigm that has been shown to robustly increase c-Fos expression in many brain regions (de Medeiros et al., 2005; Maras et al., 2014). c-Fos levels in GCs were low in unstressed control and cb δ KO animals (Figures 7B, top panels, and 7C; Figure S10). Restraint stress, however, increased c-Fos levels in GCs in control and cb δ KO animals, but regional differences were apparent (Figures 7B and 7C; Figure S10). The largest increases were observed in lobules II/II, IV/V/VI, and VIII/IX of the vermis but also in crus 1 and 2, regions implicated in social behavior (Badura et al., 2018; Stoodley et al., 2017). It is unclear whether GC hyperexcitability would increase or diminish net PC output because GCs directly excite and disynaptically inhibit PCs. We did, however, find that restraint stress increased c-Fos levels in the fastigial nucleus (FN) to a larger extent in cb δ KO animals than in control animals (Figures 7B and 7C) and that FN neurons project to brain regions involved in motor and non-motor control (Fujita et al., 2020). These observations suggest that cerebellum-specific deletion of δGABA_A_Rs can result in hyperactivation of GCs and cerebellar nuclei.

δGABA_A_ deletion also caused differential c-Fos activation in other target brain regions of the cerebellum; e.g., the thalamus (Fujita et al., 2020; Strick et al., 2009; Figure 7C; Figure S11; mediodorsal [MD] thalamus, ventral posteromedial [VPM] thalamus, and ventromedial [VM] thalamus); parabrachial nucleus (PBN; Hashimoto et al., 2018; Sadakane et al., 2000; Supple and Kapp, 1994); hippocampal CA1, CA2, and CA3 regions; dorsal caudate; basolateral amygdala (BLA); MD septum; periaqueductal gray (PAG); motor cortex (MoCtx; Figure 7B, third row); retrosplenial cortex (RspCtx; Figures 7B, fourth row, and 7C), limbic cortices (Figure 7C); and bed nucleus of the *stria terminalis* (BST; Figure S11). Conversely, decreased c-Fos levels were evident in the paraventricular nucleus of the hypothalamus (PVN; Figures 7B, bottom row, and 7C) of cb δ KO animals. In many regions, stress increased c-Fos expression to a similar extent in control and cb δ KO animals, including the lateral septum, lateral amygdala, parietal and auditory cortices, and lateral hypothalamus. Our results suggest that cerebellum-specific manipulation has widespread consequences for the activity of many downstream brain areas, contributing to diverse behavioral changes in cb δ KO animals.

## DISCUSSION

Our findings provide numerous insights into the cerebellum and behavior. We show that specific deletion of δGABA_A_ from cerebella GCs attenuates tonic inhibition and increases excitability of the input layer, resulting in differential activation of many downstream cortical and subcortical brain regions and behavioral deficits. Most importantly, we find that the cerebellum regulates behaviors that are relevant to psychiatric and neurodevelopmental disorders in a sex-specific manner. This has important implications for the many disorders that display sex differences with regard to prevalence, severity, range of symptoms, and age of onset (Goldstein et al., 2013; Merikangas and Almasy, 2020; Werling and Geschwind, 2013). Even though risk genes for sex-biased disorders like ASD and schizophrenia are highly expressed in the cerebellum (Menashe et al., 2013; Willsey et al., 2013), the cerebellum generally was not thought to contribute to sex differences in behavior. We also find that the cerebellum unexpectedly regulates maternal behaviors, which is relevant to disorders like postpartum depression. We find that, despite profound increases in input layer excitability, motor function was normal. This points toward a surprising resilience and redundancy of motor circuits.

### Hyperexcitability and Lack of Dynamic Regulation of Tonic Inhibition in GCs of cb δ KO Mice

δGABA_A_ deletion (Figure 2) from GCs, unlike ablation of α6GABA_A_ (Brickley et al., 2001), fails to sufficiently upregulate compensatory 2-pore K^+^ channels to prevent hyperexcitability. Our c-Fos experiments further support the notion that GCs in cb δ KO animals are more active during stress, suggesting that GCs are hyperexcitable *in vivo* and more responsive to excitatory input. Another important consequence of δGABA_A_ deletion is diminished responsiveness to contextual signals. During stress, neurosteroids normally decrease excitability of δGABA_A_R-expressing neurons and attenuate the stress response, but this mechanism is diminished in cb δ KO animals. Neuromodulators can also fine-tune ambient GABA levels and tonic inhibition of GCs by regulating the activity of input layer interneurons and PCs (Duguid et al., 2012; Farrant and Nusser, 2005; Guo et al., 2016; Mitchell and Silver, 2003). In addition, neurosteroids modulate δGABA_A_-containing receptors directly (Stell et al., 2003; Vicini et al., 2002) and thus regulate GC excitability during diverse physiological states, such as during the estrous cycle (Maguire et al., 2005; Wu et al., 2013), the postpartum period, and stress (Camille Melón and Maguire, 2016; Maguire and Mody, 2008; Maguire et al., 2009). For these reasons, the ability of GCs to dynamically adjust their excitability in an activity- and context-dependent manner is compromised in cb δ KOs.

### Motor Function Is Normal in cb δ KO Mice

Given the cerebellum’s role in locomotion, we were surprised to find normal motor behavior in cb δ KO mice (Figure 3). However, there is precedence for the relative insensitivity of motor performance to manipulations of the input layer (Bruinsma et al., 2015; Wiltgen et al., 2005). Our c-Fos experiments suggest that stress increases the excitability of GCs throughout the cerebellar cortex, including motor (e.g., anterior vermal lobules) and non-motor regions (crus 1/2 and posterior vermal lobules). Together, these observations suggest that basic motor function is remarkably insensitive to manipulations of the input layer.

Several explanations could account for the lack of motor deficits in cb δ KO mice. Circuit-level compensation possibly overcomes GC hyperactivation in cb δ KO mice. This could involve weakening GC-to-PC synapses. It is also possible that molecular layer interneurons (MLIs) can compensate for GC hyperexcitability by increasing inhibition of PCs. Paradoxically, our c-Fos experiments show that stress results in more pronounced c-Fos expression in the MoCtx and other motor areas in cb δ KO mice, suggesting that motor circuits might adapt better to GC hyperexcitability than non-motor systems.

### Sex-Specific Behavioral Differences Revealed in cb δ KO Mice

Unbiased behavioral testing is well-suited to characterize changes in motor and non-motor behavior arising from cerebellar manipulation. We found that cb δ KO females were more hyperactive (Figure 4), reminiscent of stimulant-treated mice (Wiltschko et al., 2020), ASD and ADHD mouse models (Angelakos et al., 2017; Dalla Vecchia et al., 2019; Schmeisser et al., 2012), and chronically stressed animals (Cabib et al., 1988; Füzesi et al., 2016; Strekalova et al., 2005). The observed sex differences in our mouse model could be due to differences in the neuroendocrine response to stress or circulating levels of sex steroids and their synthesis enzymes, all of which are related to sex differences in the stress response across species (Bangasser and Valentino, 2012; Bangasser and Wiersielis, 2018; Beery and Kaufer, 2015; Goldstein et al., 2010). These factors could lead to differential modulation of tonic currents and a greater sensitivity to δGABA_A_ deletion in females (Schüle et al., 2014; Ströhle et al., 2002; Yoshizawa et al., 2017). Another possible explanation is the well-established influence of gonadal hormones on social behavior (Bell, 2018). Finally, protein deletion can affect gene expression in a sex-dependent manner, as in Angelman syndrome (Koyavski et al., 2019). Sex differences in functional compensation have been observed previously in the cerebellum (Mercer et al., 2016). Social deficits are consistent with the cerebellum’s role in social behavior (e.g., in the context of ASD), especially the hyperactivation of crus 1 and 2 and posterior vermal lobules identified in our c-Fos study (Schmahmann, 2019; Tsai, 2016; Wang et al., 2014). What sets our findings apart from previous cerebellum-specific manipulations is that the social deficits were confined to females. With its dense expression of neurosteroid-sensitive δGABA_A_Rs, the cerebellum is poised to control behavior in a sex-specific manner. The sex specificity is intriguing because many psychiatric disorders show sex bias in humans. We speculate that δGABA_A_R-targeting treatments, such as the recently introduced postpartum depression drug brexanolone (Zulresso, Sage Therapeutics), might also be effective for treating other disorders. Clinical trials of drugs targeting δGABA_A_Rs in Angelman syndrome, a monogenic form of ASD associated with sex-dependent phenotypes (Koyavski et al., 2019), are already underway (L. Bird et al., 2019, AAN, abstract).

It is unlikely that social deficits in cb δ KO mice reflect differences in primary sensory processing. We found no difference in odor recognition, and although our mouse line expresses Cre in the cochlear nuclei, there is no δGABA_A_ expression in GCs of the ventral or dorsal cochlear nuclei (Campos et al., 2001), and hearing is normal in global δ KO mice (Maison et al., 2006). In addition, c-Fos experiments showed that activation of the auditory cortex was similar in control and cb δ KO mice (Figure 7).

### Increased Anxiety-like Behavior in cb δ KO Mice

Many clinical reports implicate the cerebellum in anxiety, phobia, generalized anxiety disorder, stress, and post-traumatic stress disorder (PTSD) (Caulfield et al., 2016; Moreno-Rius, 2018), but animal models linking the cerebellum and anxiety are rare (but see Hilber et al., 2004; Lorivel et al., 2014). Many previous studies investigating δGABA_A_Rs and anxiety focused on brain regions classically associated with fear, such as the amygdala, hypothalamus, and hippocampus (Lee et al., 2014; Liu et al., 2017; Maguire et al., 2005). Our c-Fos experiments suggest, however, that the many brain regions involved in anxiety and stress-related behaviors, such as the BLA, PAG, PVN, BST, RspCtx, and limbic cortex, are differentially activated in cb δ KO animals.

δGABA_A_ has an important role in anxiety and fear-related behaviors (MacKenzie and Maguire, 2013; Whissell et al., 2015), and endogenous compounds and drugs acting on δGABA_A_Rs affect anxiety (Eser et al., 2006). Similarly, neurosteroids and δGABA_A_ expression levels are thought to contribute to anxiety-like behaviors during the ovarian cycle (Maguire et al., 2005; Smith et al., 2006) and puberty (Smith, 2013). Ethanol, targeting extrasynaptic δGABA_A_Rs, is thought to produce its effects by increasing tonic inhibition (Richardson and Rossi, 2017). Our study establishes the importance of δGABA_A_Rs in the cb input layer in regulating anxiety and stress-related behaviors.

### Abnormal Maternal Behavior in cb δ KO Females

There is considerable evidence that δGABA_A_Rs and neurosteroid signaling are involved postpartum depression (Maguire and Mody, 2009). Although dGABA_A_s in dentate GCs (Maguire and Mody, 2008; Maguire et al., 2009) and in hippocampal parvalbumin-positive (PV+) interneurons (Ferando and Mody, 2013) have been implicated in postpartum depression, the brain regions regulating postpartum depression have not been identified directly in conditional δ KO mice. Unexpectedly, we observed abnormal maternal care in cb δ KO mice (Figure 6). Given the strong connection between anxiety, stress, and postpartum depression (Camille Melón and Maguire, 2016; Payne and Maguire, 2019), these are likely contributing factors to maternal care deficits in cb δ KO females. c-Fos experiments show differential activation in hippocampal and hypothalamic regions, but further studies are needed to determine whether δGABA_A_Rs in other brain regions act synergistically to mediate maternal behaviors.

### Differential Activation of Many Brain Regions in cb δ KO Animals in Response to Acute Stress

Emerging evidence from animal and human studies suggests that the cerebellum plays a role in the stress response (Moreno-Rius, 2019), enabled by anatomical connectivity to stress-related brain structures and its molecular machinery. Assaying restraint stress-evoked c-Fos expression provides important insights into how elimination of δGABA_A_ in GCs leads to differential activation of downstream brain regions that could account for deficits in social, anxiety-like, and parental behaviors. Stress-evoked increases in c-Fos expression in the cerebellum indicate activation of the anterior sensorimotor as well as posterior areas associated with cognition and emotion (Badura et al., 2018; Schmahmann, 2019; Stoodley et al., 2017; Strick et al., 2009). Although changes in PC activity cannot be monitored faithfully with c-Fos because of their spontaneous activity, we found increased c-Fos signal in the deep cerebellar nuclei (DCN), especially the FN, as well as in the PBN, suggesting altered PC output in cb δ KO animals. The target regions have been associated previously with cognition, emotion, and autonomic control (Berntson and Torello, 1982; Fujita et al., 2020; Sadakane et al., 2000). Stress results in hyperactivation of sensorimotor cortices and many stress-related brain structures that are thought to be modulated by the cerebellum; e.g., various regions of the cortex (Badura et al., 2018; Choe et al., 2018; Strick et al., 2009), PAG (Gonzalo-Ruiz et al., 1990; Koutsikou et al., 2014; Vaaga et al., 2020), hypothalamus (Dietrichs, 1984; Zhu et al., 2006), hippocampus (Iglói et al., 2015; Watson et al., 2019), striatum (Fujita et al., 2020), and other regions, like the BST and amygdala. In addition, hypoactivation of the PVN in cb δ KO mice can indicate hypothalamic–pituitary-adrenal (HPA) axis dysregulation and chronic stress in cb δ KO animals (Cohen et al., 2006; Kinlein et al., 2015; Whitaker and Gilpin, 2015). These observations are in line with recent tracing studies (Fujita et al., 2020; Hashimoto et al., 2018; Pisano et al., 2020) and suggest that altering sensory integration in the input layer can affect numerous circuits throughout the brain.

### Conclusions

Here we show that excitability of the cerebellar input layer can affect many behaviors and that the repertoire of cerebellum-dependent behaviors is far greater than appreciated previously. Our results substantiate the cerebellum’s role in stress and anxiety-related and social behaviors and provide insights into how the molecular make-up of the cerebellum can allow sex-specific modulation of behavior. These findings are critical for a better understanding of psychiatric and neurodevelopmental disorders that show a sex bias. Thus, we speculate that manipulating excitability of the cerebellar input layer could relieve some symptoms associated with these disorders.

## STAR★METHODS

### RESOURCE AVAILABILITY

#### Lead Contact

Further information and requests for resources and reagents should be directed to and will be fulfilled by the Lead Contact, Wade Regehr (Wade_Regehr@hms.harvard.edu).

#### Materials Availability

The study did not generate new unique reagents

#### Data and Code Availability

The datasets and code supporting the current study have not been deposited in a public repository but are available from the corresponding author on request. MoSeq code can be obtained from S.R.D. (srdatta@hms.harvard.edu)

### EXPERIMENTAL MODEL AND SUBJECT DETAILS

#### Mice

Animal procedures have been carried out in accordance with the NIH and Animal Care and Use committee (IACUC) guidelines, and protocols approved by the Harvard Medical Area Standing Committee on Animals. *Flox*ed Gabrd mice (*Gabrd*^*tm1.1Jmag*^/J, Jackson Labs stock# 023836) and Gabrd knock-out mice (*Gabrd*^*tm1Geh*^/J Jackson labs stock# 003725) were obtained from Dr. Jamie Maguire (Tufts University). Gabra6-Cre (B6.D2-Tg(Gabra6-Cre)B1Lfr/Mmucd) mice were obtained from the MMRC. A reporter line expressing tdTomato in Cre-positive cells (*floxed* tdTomato line A14) was obtained from Jackson labs (stock# 007908). Animals were kept on a mixed background (129Sv/SvJ and B6/C57). Mice were housed under standard conditions in groups of 2–5 animals on a 12 h light-dark cycle with food and water available *ad libitum*. For parental behavior assays dams were singly housed. Adult animals of either sex 2–5 months of age were used for all experiments, including FISH, immunohistochemistry, electrophysiology, and behavioral testing. For behavioral experiments and physiology, littermate controls (Cre-negative, Gabrd *f/f* or Cre-positive x +/+), cb δ KO (Cre+ x Gabrd *f/f*) and in select cases cb δ HET (Cre+ X f/f) animals were used. No difference was observed between cre-negative, Gabrd *f/f* or Cre-positive x +/+ animals and data were thus pooled).

### METHOD DETAILS

#### Fluorescence *In situ* hybridization (FISH)

Animals were anesthetized with isoflurane and the brain was rapidly removed and frozen on dry ice before embedding in optimal cutting temperature (OCT) compound (Tissue-Tek). Tissue was cut on a cryostat (Microm HM500-CM) at a thickness of 20 μm and mounted on glass slides (Superfrost Plus, VWR, 48311–703). Fluorescent *in situ* hybridization was performed according to the ACD-Bio RNAscope Multiplex Assay manual, (document Number 320513) with minor modifications. The samples were then fixed in 4% paraformaldehyde in phosphate-buffered saline (PBS) for 15 min at 4°C, then dehydrated with 50% (× 1), 70% (× 1), and 100% (× 2) ethanol washes for 5 min each. The slides were then air-dried and a barrier was drawn around the tissue section with an Immedge hydrophobic barrier pen (Vector Laboratories). The tissue was incubated in RNAscope protease III reagent (ACD-Bio 322337) at room temperature for 30 min, then rinsed twice in PBS for 5 min. Fluorophore-conjugated probes, Gabrd-Mm-C3 probe (Cat# 459481-C3), tdTomato-C2 probe (Cat# 317041) were incubated with the slide-mounted tissue sections at 40°C in a HybEz II oven (ACD-Bio) for 2 hours and washed twice in RNAscope wash buffer reagent (ACD-Bio 310091). Fluorescence amplification steps were then applied as follows: incubate in AMP 1-FL for 30 minutes at 40°C (HybEZ oven), followed with 2× wash with 1x wash buffer for 3 min at room temperature, the tissue was then incubated in AMP 2-FL for 15 minutes at 40°C, followed by 2× wash, then incubated in AMP 3-FL for 30 min at 40°C, followed with 2x wash, and lastly was incubated in AMP 4-FL-A for 15 minutes at 40 degrees, then 2× wash. Sections were then stained with DAPI and mounted with ProLong antifade reagent (Thermo Fisher Scientific P36930). In addition, control probes were used to ensure the quality of the *in situ* experiment. The positive control is a cocktail of housekeeping genes (C1-Mm-Polr2a, C2-Mm PPIB and C3-Mm-UBC). The negative control probe targets bacterial RNA (C1, C2, C3-dapB). The slides were imaged using whole slide scanning microscope (Olympus VS120) with a 20X air objective.

#### Immunohistochemistry

Mice were anesthetized with isoflurane and perfused with ice cold phosphate buffered saline (PBS, pH = 7.4, Sigma Cat# P-3813), followed by a solution containing 4% paraformaldehyde in PBS. The brain was removed and postfixed in the same solution at 4°C overnight. For slicing, the brain was embedded in 4% agar (Sea Plaque, Lonza, Cat# 50101) and then sliced in PBS using a vibratome (VT1000S, Leica) at a thickness of 50 μm. Antigen retrieval was performed prior to immunostaining. Slices were permeabilized in 0.2% TritonX (Sigma Cat# T9284) in PBS for 30 min and then incubated in a solution containing 0.001% trypsin (Sigma Cat# T5266) and 0.001% Ca_2_Cl in PBS for 1 minute. Slices were then rinsed 3 times for 5 minutes in PBS and then blocked in a solution containing 4% normal goat serum (NGS), 0.1% TritonX in PBS for 1 hour. After blocking, slices were incubated in the same solution with the addition of primary antibody and 0.001% trypsin inhibitor (Sigma Cat# T6522) overnight at 4°C. Slices were then washed 3 times for 10 minutes an incubated in 4% NGS, 0.1% TritonX in PBS with addition of secondary antibody for 2 hours at room temperature. Slices were then washed 3 times for 5 minutes in PBS, mounted on glass slides (Superfrost Plus, VWR, Cat# 48311–703) and covered with mounting medium (ProLongDiamond, Thermo Fisher Scientific, Cat# P36961) and a glass coverslip. The mounting medium was allowed to cure for at least 24 hours before imaging.

#### Imaging and image analysis

Whole-brain images were taken on an Olympus VS120 slide scanner, and confocal stacks were acquired on an Olympus FV1000 confocal microscope. Images were processed using standard routines in Fiji (ImageJ). Some figures (Graphical Abstract and Figures 4A, 4D, 6A, 6G, and 7A) were prepared with BioRender (Biorender.com).

#### Quantitative western blotting

Western blotting was performed according to standard protocols. For quantitative assessment of protein levels in mice brain tissues, fluorescently tagged secondary antibodies were used. Prior to harvesting tissue mice where anesthetized with isoflurane and perfused transcardially with ice cold PBS. The brain was then rapidly removed from the skull and tissue was dissected in PBS and frozen at −80°C until further processing. Tissue was lysed in a buffer containing 150 mM NaCl, 25 mM HEPES, 4 mM EGTA and protease inhibitor (Sigma-Aldrich, Cat# P8340). After addition of SDS harvested tissue underwent ten freeze-thaw cycles (−80 to +55°C). After SDS-PAGE, gels were transferred onto nitrocellulose membranes and blocked with filtered 5% nonfat milk/5% goat serum in Tris-buffered saline for one hour at room temperature and incubated with primary antibodies with 5% BSA in Tris-buffered saline containing 0.1% Tween-20 (TBST) overnight at 4°C. Each membrane was incubated with primary antibodies against GABA_A_ receptor subunits as follows: rabbit anti-α1GABA_A_R(1:10 000; Abcam, Cat# ab33299, RRID: AB_732498), rabbit anti- α6GABA_A_R (1:1000; GeneTex, Cat# GTX130947), mouse anti- β3GABA_A_R (Neuromab, Cat# 75–149, RRID: AB_2109585), rabbit anti- γ2 GABA_A_R (1:1000; Synaptic Systems, Cat# 224 003, RRID: AB_2263066), rabbit anti- δGABA_A_R (1:2000; custom made, gift from Dr. W. Sieghart and Dr. P. Scholze, Medical University of Vienna, Vienna, Austria). The following fluorescent secondary antibodies were used prior to washing with TBST: donkey anti-mouse IRDye 800CW IgG (1:10,000; LI-COR, Cat. No.: 926–32212, RRID: AB_621847), donkey anti-rabbit IRDye 800CW IgG (1:10,000; LI-COR, Cat. No.:926–32213, RRID: AB_621848), donkey anti-mouse IRDye 680RD IgG (1:10,000; LI-COR, Cat. No.:926–32222, RRID: AB_621844), and donkey anti-rabbit IRDye 680RD IgG (1:10,000; LI-COR, Cat. No.:926–32223, RRID: AB_621845). Visualization was carried out with the LI-COR Odyssey® fluorescent scanner and software (LI-COR Biosciences). Blots were imaged using an Odyssey Infrared Imaging System Scan, at a resolution of 42 μm. Images were analyzed in NIH ImageJ Software.

#### Slice preparation for electrophysiology

Mice to be used for electrophysiological recordings were retrieved from the animal facility and allowed to acclimate for at least 8 hours before the experiment to reduce stress. Animals of either sex aged 2–4 months were anesthetized in their home cage by introduction of an isoflurane-soaked cloth and then perfused transcardially under continued isoflurane anesthesia with ice cold cutting solution containing (in mM) 110 CholineCl, 7 MgCl_2_, 2.5 KCl, 1.25 NaH_2_PO_4_, 0.5 CaCl_2_, 25 Glucose, 11.5 Na-ascorbate, 3 Na-pyruvate, 25 NaHCO_3_, 0.003 (R)-CPP, equilibrated with 95% O_2_ and 5% CO_2_. The brain was rapidly dissected and the cerebellum was cut into 250–270 μm thick parasagittal slices in the same solution on a vibratome (VT1200S, Leica). Slices were then transferred to 34°C warm artificial cerebrospinal fluid (ACSF) containing (in mM) 125 NaCl, 26 NaHCO_3_, 1.25 NaH_2_PO_4_, 2.5 KCl, 1 MgCl_2_, 1.5 CaCl_2_, and 25 glucose, equilibrated with 95% O_2_ and 5% CO_2_ and incubated for 30 min. Slices were then stored at room temperature until recording for up to 6 hours.

#### Electrophysiology

Whole-cell recordings were obtained from visually identified granule cells using a 40× water-immersion objective on an upright microscope (Olympus BX51WI). Pipettes were pulled from BF150-86-10 borosilicate glass (Sutter Instrument Co., Novato, CA) at resistances of 4–5 MΩ on a Sutter P-97 horizontal puller. Electrophysiological recordings were performed at ~32°C. For voltage clamp recordings, the internal solution contained (in mM): 30 K-gluconate, 110 KCl, 10 HEPES, 0.5 EGTA, 3 MgATP, 0.5 Na_3_GTP, 5 phosphocreatine-tris_2_, 5 phosphocreatine-Na_2_, 5 QX314 chloride. The chloride reversal potential was ~0 mV. For current clamp recordings, the internal solution contained (in mM) 130 K-gluconate, 10 KCl, 10 HEPES, 0.5 EGTA, 3 MgATP, 0.5 Na_3_GTP, 5 phosphocreatine-tris_2_, 5 phosphocreatine-Na_2_. The chloride reversal potential was ~−65 mV, similar to what was reported for GCs (Brickley et al., 1996). Seal resistance for all GC recordings was > 5 GΩ. Electrophysiology data were acquired using a Multiclamp 700B amplifier (Axon Instruments), digitized at 50 kHz, filtered at 4 kHz, and controlled by software custom written in IGOR Pro (Lake Oswego, OR). The recording ACSF included (in μM): 2.5 (R)-CPP, 5 NBQX hydrochloride, 2 CGP, 1 strychnine to block glutamatergic receptors, GABA_B_ receptors and glycine receptors, respectively. SR 95531 (100 μM) was used to block GABA_A_ receptor-mediated currents. The δGABA_A_R preferring agonist 4,5,6,7-tetrahydroisoxazolo[5,4-c]pyridin-3-ol (THIP, 1 μM) was used to activate tonic GABAergic currents. All drugs were purchased from Abcam (Cambridge, MA) or Tocris (Bristol, UK). Analysis of electrophysiological data was performed with custom routines written in IgorPro (Wavementrics, Lake Oswego, OR) or in AxoGraphX.

#### Behavioral testing

All behavior testing was performed in adult mice (> 8 weeks old) of either sex with the experimenter blind to the genotype. Before behavioral testing, mice were transferred to the behavior room and allowed to acclimate for at least 30 min. Animals were housed on a 12 h light-dark cycle, and the experiments were carried out at the beginning of the dark cycle. All equipment used for behavioral testing was cleaned with 70% ethanol in between experiments. Most behavioral testing was videotaped at ~30 fps using a 720p USB Camera with IR LEDs (ELP, Ailipu Technology Co.) and the iSpy open source video surveillance software suite (https://www.ispyconnect.com). Offline analysis was automated using custom scripts written in MATLAB (Mathworks). Select behaviors were scored live (pup retrieval, olfactory testing), or analyses was carried out manually (grooming, forced swim).

#### Gait analysis

To assess baseline locomotion, gait analysis was run over the course of 8 consecutive days with 5 trials done for each animal per day. A custom video recording setup was used for evaluating gait patterns in mice. The setup consisted of an infrared illuminated transparent linear corridor (64.5(L) × 4 (W) × 6 (H) cm) atop a borosilicate glass floor. The corridor was flanked by two mirrors on the side angled at 48 degrees to project the images of the side views toward a video camera (Bonito CL-400B/C 2320 × 700 pixels at 200 frames per second, Allied Vision, Exton, PA.) situated beneath the glass floor. Three views were captured simultaneously at 200 fps as the animal walk down the linear corridor in a self-initiated manner. To annotate the locations of the body parts (nose, base of the tail, tip of the tail, left/right forepaws, and left/right hind paws), a convolutional neural network based on the stacked hour-glass-network (Newell et. al., 2016) was trained using PyTorch on 500 manually annotated sample frames. We used the neural network to annotate frame-by-frame which results in a time series for the location of all aforementioned body parts. A Hidden Markov Model (HMM) was applied on the time series to segment each video into individual gait cycle. For a single gait cycle, we measured the following parameters: cadence, the number of cycles per second in Hz; stride length, the maximum distance traveled by the forepaw within a single cycle; paw width, the lateral distance between the two diagonal supports at any given time; stance duration, the average amount of time that the paws are on the ground during; velocity, the average speed of the center of mass; and tail elevation, the absolute height of the tip of the tail from the floor. We used a linear mixed effect model of the form Y ~Intercept + genotype + sex + genotype:sex + (1|name) and ANOVA for statistical test in the non-covariate case. For the covariate analysis involving the velocity we fitted a model of the form Y ~Intercept + Velocity + genotype + sex + genotype:sex + velocity:genotype + velocity:sex + (1|name) and ANOVA for testing statistical significance.

#### Rotarod

To evaluate motor learning, we performed a rotarod assay over the course of three consecutive days, with 5 trials performed each day. Mice were placed on a rotating rod device (Rotamex-5 Rota-Rod, Columbus Instruments, controlled by Rotamex-5 software) running at 4 rpm baseline speed. After brief habituation, acceleration of the rod was initiated. The rod accelerated from an initial speed of 4 rpm to a maximum speed of 40 rpm in 10 s intervals. An animal fall was detected by infrared photo cells crossing the space above the rod and the time-to-fall was recorded. A fall was recorded either if the mouse fell off the rod, or loops around the rod without running. Animals were allowed to rest for 1 min between trials.

#### Eyeblink conditioning

Adult mice of either sex performed motor behavior experiments during the dark cycle when they are most active and alert. The setup was housed within a ventilated, anechoic, and sound-insulated behavior chamber. Prior to the experiment, animals were implanted with a head bracket and allowed to recover for four days of post-surgery. To accustom the animals to head-fixation and the treadmill, five days of habituation were performed prior to the first day of eyeblink training. During habituation, the animal was head-fixed atop of a motorized treadmill six inches in diameter rotating at 20 mm/s. The treadmill was kept at this speed throughout the habituation period. During training, a white LED flash was used as the conditioned stimulus (CS) and a 50 ms, 15 psi air puff directed at the opposite eye was delivered as the unconditioned stimulus (US). The air puff was delivered via a 21-gauge blunt tip needle mounted on a manipulator to allow for individual adjustments. The pneumatic and electronics necessary for the control of the air puff was based on the design of Openspritzer (Forman et al., 2017). To record eyelid movement, we illuminated the behavior chamber using an IR lamp and recorded the eye with a high-speed camera (Mako U-029B, Allied Vision, Exton, PA.) and a macro lens (1/2” 4–12mm F/1.2, Tamron, Commack, NY.) at 300 fps. The vertical span of the eyelid opening was measured as a function over time using a custom MATLAB script. An inter-stimulus-interval of 500 ms was used. 100 trials of CS-US pairing and 10 trials of only the CS were presented to the animal per day for 15 days. The inter-trial interval was randomized between 4 to12 s.

#### Open field

Animals were placed in an uncovered rectangular behavior arena (30.3 cm × 45.7 cm, 30.5 cm high) containing fresh bedding and observed for 10 min without intervention. Analysis was performed offline using MATLAB (position, velocity, path traveled).

#### 3-chamber assay

Sociability was assayed with the 3-chamber task. The behavioral arena consisted of a clear rectangular Plexiglas box (40.5 cm wide, 60 cm long, 22 cm high) without a top cover and divided into 3 equally sized compartments by clear walls. Each divider contained a 10.2 cm × 5.4 cm rectangular opening to allow navigation between the compartments. The left and the right chamber contained inverted wire cups (10 cm in diameter). Before behavioral testing, mice were allowed to navigate the middle chamber for 5 minutes with openings to the adjacent chambers closed. During a 10-minute habituation session, the doors were then opened and mice were allowed to freely navigate all 3 chambers for 10 minutes in the absence of any stimuli. After habituation, the doors were closed again while the animal remained in the central chamber. A social stimulus (juvenile mouse aged 15–30 days of the same sex and strain) and a novel object (mouse-sized plastic toy, Schleich GmbH, Germany) were placed in opposite wire cups. The sides of social and non-social stimuli were randomly selected to control for preference to either side of the arena. After stimulus placement, the doors were opened and the animal was observed for another 10 minutes. Automated analysis was performed offline using MATLAB.

#### Light/dark chamber

A light-dark chamber assay was used to measure anxiety-like in adult mice. The experiment consisted of a Plexiglas arena with the same outer dimensions as used for the 3-chamber assay. The light-dark chamber arena consisted of two chambers, with one removable divider, including a door which remained open throughout. The light chamber was 40.5 cm × 40 cm chamber and brightly lit (> 600 lux) and uncovered. The dark chamber was 40.5 cm × 20 cm and covered with darkly tinted Plexiglass that allowed videotaping of the mouse with an IR camera. Light intensity inside the dark chamber was < 10 lux. At the beginning of the experiment, the animal was placed in the dark chamber and was allowed to freely navigate both chambers for 10 min.

#### Motion Sequencing

Motion Sequencing (MoSeq)-based behavioral analysis was performed as in Wiltschko et al., 2015 and Markowitz et al., 2018. In brief, MoSeq uses unsupervised machine learning techniques to identify the number and content of behavioral syllables out of which mice compose their behavior; identifying these syllables allows each video frame of a mouse behavioral experiment to be assigned a label identifying which syllable is being expressed at any moment in time. Behavioral phenotypes that distinguish wild-type and mutant mice can be identified by comparing differences in how often individual syllables are used in a given experiment. Here, individual mice (n = 8 males and 8 females for each genotype) were imaged for 1–3 60-minute-long sessions using a Kinect2 depth sensor while behaving in a circular open field under red light illumination. These 3d imaging data were submitted to the MoSeq pipeline, which includes mouse extraction, denoising, and alignment steps before computational modeling. As has been done previously (Wiltschko et al., 2015), the kappa parameter (which sets the timescale at which syllables are identified) was specified by matching the distribution of syllable durations to a model-free changepoint distribution.

Separate models were trained for males and females. All mice of a given gender were jointly modeled with a shared transition. MoSeq identified 61 syllables in females and 64 syllables in males that make up 90% of frames that comprise each dataset. For convenience, in Figure 5, we depict genotype-driven differences in the top 28 used syllables with each accounting for at least 1% of the data in each genotype and gender. Together, these comprise 47% and 56% of the frames in the female and male datasets respectively. Significant differences in syllable expression between genotypes were determined using the Mann-Whitney U test, with the Benjamini and Hochberg correction for multiple comparisons at an alpha level of 0.05.

Because males and females were modeled independently, identified syllables were matched across genotypes but not across genders. In order to register syllable identity across genders, for each female syllable depicted in Figure 5, the best-matching male syllable was identified by computing pairwise Pearson’s correlation distances between that syllable and every available male syllable. Distances were computed over sets of scalar values derived from raw depth footage associated with a particular syllable such as 2-d, 3-d and angular velocity, mouse length, width, and height.

Identified syllables represent gross behavioral differences like running versus rearing, but also distinguish closely related behaviors like slow and fast running or low and high rears. In order to depict the extent to which behavior differs between wild-type and knock-out mice, pairwise syllable distances were computed between the 10 most upregulated syllables within each cohort as well as between the 10 most differentially upregulated syllables across cohorts and depicted as probability density distributions in Figure 5. If knock-out and wild-type mice exhibit gross and consistent behavioral differences, pairwise syllable distances, on average, will be greater across cohorts relative to within-cohort comparisons. Alternatively, if both knock-out and wild-type mice express similar behaviors, within and across-cohort pairwise distance distributions should be more similar.

#### Pup interaction assay and spontaneous retrieval

Naive virgin females were placed in a behavioral arena (30 cm × 46 cm, 31 cm high) without a top containing fresh standard bedding and were allowed to explore the arena freely during a 10-minute habituation period. After habituation, 3 pups of the same mouse strain aged P1-P3 were placed on one side, and 3 pup-sized novel plastic objects were placed on the opposite side of the arena. Interactions of the female with the pups and novel objects were recorded for 10 minutes (as defined as a 2.5 cm radius around the nest or the center of the toys). After 10 minutes one pup was removed from the nest and placed in the center of the behavioral arena. If retrieval of the pup had not occurred within the observation period the trial was counted as a failure to retrieve and was terminated. Pups were promptly returned to their mothers after the experiment. Analysis was performed offline using MATLAB.

#### Postpartum pup retrieval and cannibalization

The dam was allowed to remain in her home cage and 3 pups were removed from the nest and placed in the center and 2 corners of the cage opposing the nest (see Figure 5A for schematic). The time to retrieve each pup to the nest was scored once a day over a time period of 3 consecutive days, starting on P0. Care was taken not to disturb the dam and her pups unnecessarily during this period. Pups were counted on P0-P3 and cannibalization was noted. Cannibalization mostly occurred on P0 and P1 and rarely thereafter.

#### Pharmacological rescue of parental behavior

For the rescue of pup interaction virgin females were either tested under control (regular drinking water) or test conditions (after administration of THIP for 24–48 h, THIP was dissolved in the drinking water at a dose of 5 mg/kg similar to a previously described paradigm (Maguire and Mody, 2008). The dose had only minor effects on locomotion, suggesting it was non-sedating. Administration with the drinking water precluded excessive handling and resulting stress in the animals. The pup interaction assay was otherwise conducted and analyzed as described above. Each female was tested under control and test conditions, and the order was randomly assigned to account for learning effects (ref). If the animal was tested first under test conditions, the animal was allowed a resting period of at least 72 h to ensure clearance of THIP from the body. Drinking water consumption was recorded before each behavior experiment (control water consumption 8.0 mL ± 0.9 ml/per mouse/24 h THIP water consumption 8.9 mL ± 1.9 ml/mouse/24 h, p > 0.6, Student’s t test)

For the rescue of postpartum maternal behavior timed-pregnant dams were randomly assigned to either the control or THIP group. THIP was administered with the drinking water 72 – 48 h before birth until 48 h after birth. Only first-time pregnancies were scored. Postpartum retrieval and pup cannibalization were recorded from P0 – P3 as described above.

#### Forced swim

Mice were placed in a glass cylinder (height, diameter) filled with water at room temperature and observed for a 6-min period. Animals were then removed from the beaker, towel dried, and returned to their home cage. Videos were scored offline. Time spent mobile (swimming, actively struggling) and immobile (floating, with front paws and at least one hind paw immobile) were scored.

#### Olfactory testing

To assay odor discrimination we performed a simple olfaction task. Animals were introduced to a clean, covered cage without bedding, food or water. Three neutral, non-social odors (coconut, raspberry, banana), 2 social scents (female and male urine), as well as a water sample were pipetted onto a clean cotton swab and introduced consecutively to the cage at random order. Mice were allowed to explore each scent for 5 min, and time spent investigating (sniffing, biting or chewing) was recorded live by the investigator.

#### Whole brain c-Fos expression analysis

Control and cb δ KO littermates were single-housed a d either exposed to an acute stressor for 30 min (restraint in a DecapiCone, Braintree Scientific, #MDC-200), or remained in their home cage until sacrifice. After restraint, the animals were allowed to recover in their home cages for 60 min, after which they were anesthetized with isoflurane in their home cage. The thoracic cavity was opened and blood was withdrawn from the left ventricle of the heart and transferred to a heparin-coated vial (BD microtainer #365965) on ice. Blood plasma was reserved for further analysis. After blood collection, the animals were perfused transcardially with PBS and 4% PFA. Brains were removed and stored in 4% PFA at 4 degrees overnight. Brains were then sliced on a vibratome (Leica VT 1000S) at 40 μm thickness. Immunostaining for c-Fos was performed as described above (dilution of c-Fos primary antibody 1:1000 for 48 h at 4°C, #2250, Cell Signaling, Alexa 647 or Alexa 488 secondary antibody, 1:1000 dilution for 24 h at 4°C). Slices were then washed, mounted, and coverslipped. Images were taken on an Olympus VS120 slide scanner, and confocal stacks were acquired on an Olympus FV1000 confocal microscope. Raw images were processed for quantification of c-Fos expression in ImageJ. The Allen Brain Institute reference atlas (allenbrain.org) was used to identify brain regions across sections. Regions of interest were thresholded to create a mask outlining c-Fos positive nuclei, and particles consistent with nuclear shape (diameter, circularity) were counted. Heatmaps represents values of c-Fos particles per region normalized to control conditions (no stress), and the ratio (cb KO stress/control stress)/control stress. C-Fos expression was quantified in tissue of two animals per condition (total of 8 animals).

### QUANTIFICATION AND STATISTICAL ANALYSIS

Electrophysiology data analysis was performed in Igor Pro (Wavemetrics), AxographX (Axograph) and Prism (Graphpad). The numbers of cells recorded are indicated in the figure legends and in the text. To determine significance in a dataset the Whitney-Mann test, Wilcoxon signed rank test or Kruskal-Wallis test (with Dunn’s post-test) were used, as indicated. For select datasets, a one-sample t test (Western blot analysis), or one-way ANOVA (gait analysis, with Dunnet’s multiple comparison post-test), were performed. Behavioral analysis was performed in MATLAB (Mathworks) using custom written scripts, and Prism. Unpaired Student’s t test was used to determine statistical significance. The number of animals used for each dataset is indicated in the text or figure legend.

## Supplementary Material

1

## Figures and Tables

**Table:** KEY RESOURCES TABLE

REAGENT or RESOURCE	SOURCE	IDENTIFIER
Chemicals, Peptides, and Recombinant Proteins		
NBQX disodium salt	Abcam Cambridge, MA	Cat# ab120046
(R)-CPP	Abcam Cambridge, MA	Cat# ab120159
SR95531 (gabazine)	Abcam Cambridge, MA	Cat# ab120042
Strychnine HCl	Sigma Aldrich	Cat# S-8753
QX314 chloride	Abcam Cambridge, MA	Cat# ab120118
CGP 55845 hydrochloride	Abcam Cambridge, MA	Cat# ab120337
THIP hydrochloride (gaboxadol	Abcam Cambridge, MA	Cat# ab120426
RNAscope fluorescent kit	ACD, Cambridge, MA	Cat# 320850
RNAscope Gabrd-Mm-C3 probe	ACD, Cambridge, MA	Cat# 459481-C3
RNAscope tdTomato- probe	ACD, Cambridge, MA	Cat# 317041
Anti-GABA_A_ δ Antibody, rabbit	Alomone labs, Jerusalem, Israel	Cat# AGA-014; RRID: AB_2340938
Anti-rabbit Alexa 647	Abcam, Cambridge, MA	Cat# 150083; RRID: AB_2714032
Anti-rabbit Alexa 488	Abcam Cambridge, MA	Cat# 150081; RRID: AB_2734747
rabbit anti-GABA_A_ receptor-α1	Abcam	Cat# ab33299; RRID: AB_732498
rabbit anti-GABA_A_ receptor-α6	GeneTex	Cat# GTX130947
mouse anti-GABA_A_ receptor-β3	Neuromab	Cat# 75–149; RRID: AB_2109585
rabbit anti-GABA receptor-γ2	Synaptic Systems	Cat# 224 003; RRID: AB_2263066
rabbit anti-GABA_A_ receptor-δ 1:2000	gift from Dr. W. Sieghart and Dr. Petra Scholze, Medical University of Vienna, Austria	N/A
rabbit anti c-fos (9F6)	Cell Signaling Technology	Cat# 2250S; RRID: AB_2247211
Experimental models: Organisms/ Strains		
*floxed* Gabrd mice *(Gabrd*^*tm1.1Jmag*^*/J)*	Dr. Jamie Maguire, Tufts University Jackson Labs	Stock# 023836 (Lee et al., 2014)
Gabrd knock out mice *(Gabrd*^*tm1Geh*^*/J)*	Dr. Gregg Homanics, University of Pittsburgh Jackson labs	Stock# 003725 (Mihalek et al., 1999)
Gabra6-cre mice B6.D2-Tg(Gabra6-cre) B1Lfr/Mmucd	MMRC	RRID: MMRRC_015966-UCD (Fünfschilling and Reichardt, 2002)
Floxed tdTomato reporter line (Ai14, Gt(ROSA)26Sor^tm14(CAG-tdTomato)Hze^/J)	Jackson Labs	Stock# 007908 (Madisen et al., 2010)
Software and Algorithms		
Igor Pro 6	Wavemetrics	https://www.wavemetrics.com/
MafPC	Courtesy of M.A. Xu-Friedman	https://www.xufriedman.org/mafpc
MATLAB (R2017a)	MathWorks	https://www.mathworks.com/
Fiji (ImageJ)	NIH	https://fiji.sc/
AxographX 1.7.0	Axograph	https://axograph.com/
Prism 6	Graphpad	https://www.graphpad.com/
Adobe Illustrator	Adobe	https://www.adobe.com/products/illustrator.html
Moseq2	Sandeep Datta Laboratory	https://github.com/orgs/dattalab/teams/moseq2-users
Python	Python	https://www.python.org/
Biorender	Biorender	https://biorender.com/

## Highlights

- δGABA_A_ deletion in cerebellar granule cells causes input layer hyperexcitability
- Hyperexcitability leads to increased anxiety- and stress-related behaviors
- Females lacking cerebellar δGABA_A_ show deficits in social and maternal behavior
- Hyperactivation of granule cells during stress alters activity in many brain regions

## References

[R1] Albergaria C, Silva NT, Pritchett DL, and Carey MR (2018). Locomotor activity modulates associative learning in mouse cerebellum. Nat. Neurosci 21, 725–735. 29662214 10.1038/s41593-018-0129-x PMC5923878

[R2] Albus JS (1971). A theory of cerebellar function. Math. Biosci 10, 25–61.

[R3] Angelakos CC, Watson AJ, O’Brien WT, Krainock KS, Nickl-Jockschat T, and Abel T (2017). Hyperactivity and male-specific sleep deficits in the 16p11.2 deletion mouse model of autism. Autism Res 10, 572–584. 27739237 10.1002/aur.1707 PMC6201314

[R4] Badura A, and De Zeeuw CI (2017). Cerebellar Granule Cells: Dense, Rich and Evolving Representations. Curr. Biol 27, R415–R418. 28586665 10.1016/j.cub.2017.04.009

[R5] Badura A, Verpeut JL, Metzger JW, Pereira TD, Pisano TJ, Deverett B, Bakshinskaya DE, and Wang SS-H (2018). Normal cognitive and social development require posterior cerebellar activity. eLife 7, e36401. 30226467 10.7554/eLife.36401 PMC6195348

[R6] Bangasser DA, and Valentino RJ (2012). Sex differences in molecular and cellular substrates of stress. Cell. Mol. Neurobiol 32, 709–723. 22488525 10.1007/s10571-012-9824-4 PMC3378920

[R7] Bangasser DA, and Wiersielis KR (2018). Sex differences in stress responses: a critical role for corticotropin-releasing factor. Hormones (Athens) 17, 5–13. 29858858 10.1007/s42000-018-0002-z

[R8] Becker EBE, and Stoodley CJ (2013). Autism spectrum disorder and the cerebellum. Int. Rev. Neurobiol 113, 1–34. 24290381 10.1016/B978-0-12-418700-9.00001-0

[R9] Beery AK, and Kaufer D (2015). Stress, social behavior, and resilience: insights from rodents. Neurobiol. Stress 1, 116–127. 25562050 10.1016/j.ynstr.2014.10.004 PMC4281833

[R10] Bell MR (2018). Comparing Postnatal Development of Gonadal Hormones and Associated Social Behaviors in Rats, Mice, and Humans. Endocrinology 159, 2596–2613. 29767714 10.1210/en.2018-00220 PMC6692888

[R11] Berntson GG, and Torello MW (1982). The paleocerebellum and the integration of behavioral function. Physiol. Psychol 10, 2–12.

[R12] Braat S, and Kooy RF (2015). Insights into GABAAergic system deficits in fragile X syndrome lead to clinical trials. Neuropharmacology 88, 48–54. 25016041 10.1016/j.neuropharm.2014.06.028

[R13] Brickley SG, Cull-Candy SG, and Farrant M (1996). Development of a tonic form of synaptic inhibition in rat cerebellar granule cells resulting from persistent activation of GABAA receptors. J. Physiol 497 (Pt 3), 753–759. 9003560 10.1113/jphysiol.1996.sp021806 PMC1160971

[R14] Brickley SG, Revilla V, Cull-Candy SG, Wisden W, and Farrant M (2001). Adaptive regulation of neuronal excitability by a voltage-independent potassium conductance. Nature 409, 88–92. 11343119 10.1038/35051086

[R15] Bridi MS, Park SM, and Huang S (2017). Developmental Disruption of GABAAR-Meditated Inhibition in Cntnap2 KO Mice. eNeuro 4, ENEURO.0162–17.2017. 10.1523/ENEURO.0162-17.2017 PMC5617210 28966979

[R16] Bruinsma CF, Schonewille M, Gao Z, Aronica EMA, Judson MC, Philpot BD, Hoebeek FE, van Woerden GM, De Zeeuw CI, and Elgersma Y (2015). Dissociation of locomotor and cerebellar deficits in a murine Angelman syndrome model. J. Clin. Invest 125, 4305–4315. 26485287 10.1172/JCI83541 PMC4639977

[R17] Buckner RL (2013). The cerebellum and cognitive function: 25 years of insight from anatomy and neuroimaging. Neuron 80, 807–815. 24183029 10.1016/j.neuron.2013.10.044

[R18] Bullitt E (1990). Expression of c-fos-like protein as a marker for neuronal activity following noxious stimulation in the rat. J. Comp. Neurol 296, 517–530. 2113539 10.1002/cne.902960402

[R19] Cabib S, Kempf E, Schleef C, Mele A, and Puglisi-Allegra S (1988). Different effects of acute and chronic stress on two dopamine-mediated behaviors in the mouse. Physiol. Behav 43, 223–227. 3212060 10.1016/0031-9384(88)90242-9

[R20] Calamandrei G, and Keverne EB (1994). Differential expression of Fos protein in the brain of female mice dependent on pup sensory cues and maternal experience. Behav. Neurosci 108, 113–120. 8192837 10.1037//0735-7044.108.1.113

[R21] Camille Melón L, and Maguire J (2016). GABAergic regulation of the HPA and HPG axes and the impact of stress on reproductive function. J. Steroid Biochem. Mol. Biol 160, 196–203. 26690789 10.1016/j.jsbmb.2015.11.019 PMC4861672

[R22] Campos ML, de Cabo C, Wisden W, Juiz JM, and Merlo D (2001). Expression of GABA(A) receptor subunits in rat brainstem auditory pathways: cochlear nuclei, superior olivary complex and nucleus of the lateral lemniscus. Neuroscience 102, 625–638. 11226699 10.1016/s0306-4522(00)00525-x

[R23] Carta I, Chen CH, Schott AL, Dorizan S, and Khodakhah K (2019). Cerebellar modulation of the reward circuitry and social behavior. Science 363, eaav0581. 30655412 10.1126/science.aav0581 PMC6711161

[R24] Carver CM, Wu X, Gangisetty O, and Reddy DS (2014). Perimenstrual-like hormonal regulation of extrasynaptic δ-containing GABAA receptors mediating tonic inhibition and neurosteroid sensitivity. J. Neurosci 34, 14181–14197. 25339733 10.1523/JNEUROSCI.0596-14.2014 PMC4205546

[R25] Caulfield MD, Zhu DC, McAuley JD, and Servatius RJ (2016). Individual differences in resting-state functional connectivity with the executive network: support for a cerebellar role in anxiety vulnerability. Brain Struct. Funct 221, 3081–3093. 26231515 10.1007/s00429-015-1088-6

[R26] Cavelier P, Hamann M, Rossi D, Mobbs P, and Attwell D (2005). Tonic excitation and inhibition of neurons: ambient transmitter sources and computational consequences. Prog. Biophys. Mol. Biol 87, 3–16. 15471587 10.1016/j.pbiomolbio.2004.06.001 PMC8906495

[R27] Cayco-Gajic NA, and Silver RA (2019). Re-evaluating Circuit Mechanisms Underlying Pattern Separation. Neuron 101, 584–602. 30790539 10.1016/j.neuron.2019.01.044 PMC7028396

[R28] Chabrol FP, Arenz A, Wiechert MT, Margrie TW, and DiGregorio DA (2015). Synaptic diversity enables temporal coding of coincident multisensory inputs in single neurons. Nat. Neurosci 18, 718–727. 25821914 10.1038/nn.3974 PMC4413433

[R29] Chandra D, Jia F, Liang J, Peng Z, Suryanarayanan A, Werner DF, Spigelman I, Houser CR, Olsen RW, Harrison NL, and Homanics GE (2006). GABAA receptor alpha 4 subunits mediate extrasynaptic inhibition in thalamus and dentate gyrus and the action of gaboxadol. Proc. Natl. Acad. Sci. USA 103, 15230–15235. 17005728 10.1073/pnas.0604304103 PMC1578762

[R30] Choe KY, Sanchez CF, Harris NG, Otis TS, and Mathews PJ (2018). Optogenetic fMRI and electrophysiological identification of region-specific connectivity between the cerebellar cortex and forebrain. Neuroimage 173, 370–383. 29496611 10.1016/j.neuroimage.2018.02.047 PMC5911204

[R31] Cogram P, Deacon RMJ, Warner-Schmidt JL, von Schimmelmann MJ, Abrahams BS, and During MJ (2019). Gaboxadol Normalizes Behavioral Abnormalities in a Mouse Model of Fragile X Syndrome. Front. Behav. Neurosci 13, 141. 31293404 10.3389/fnbeh.2019.00141 PMC6603241

[R32] Cohen H, Zohar J, Gidron Y, Matar MA, Belkind D, Loewenthal U, Kozlovsky N, and Kaplan Z (2006). Blunted HPA axis response to stress influences susceptibility to posttraumatic stress response in rats. Biol. Psychiatry 59, 1208–1218. 16458266 10.1016/j.biopsych.2005.12.003

[R33] Crawley JN (1985). Exploratory behavior models of anxiety in mice. Neurosci. Biobehav. Rev 9, 37–44. 2858080 10.1016/0149-7634(85)90030-2

[R34] Dalla Vecchia E, Mortimer N, Palladino VS, Kittel-Schneider S, Lesch K-P, Reif A, Schenck A, and Norton WHJ (2019). Cross-species models of attention-deficit/hyperactivity disorder and autism spectrum disorder: lessons from CNTNAP2, ADGRL3, and PARK2. Psychiatr. Genet 29, 1–17. 30376466 10.1097/YPG.0000000000000211 PMC7654943

[R35] de Medeiros MA, Carlos Reis L, and Eugênio Mello L (2005). Stress-induced c-Fos expression is differentially modulated by dexamethasone, diazepam and imipramine. Neuropsychopharmacology 30, 1246–1256. 15714225 10.1038/sj.npp.1300694

[R36] Dietrichs E (1984). Cerebellar autonomic function: direct hypothalamocere-bellar pathway. Science 223, 591–593. 6198719 10.1126/science.6198719

[R37] Duguid I, Branco T, London M, Chadderton P, and Häusser M (2012). Tonic inhibition enhances fidelity of sensory information transmission in the cerebellar cortex. J. Neurosci 32, 11132–11143. 22875944 10.1523/JNEUROSCI.0460-12.2012 PMC6363100

[R38] Eser D, Romeo E, Baghai TC, Schüle C, Zwanzger P, and Rupprecht R (2006). Neuroactive steroids as modulators of depression and anxiety. Expert Rev. Endocrinol. Metab 1, 517–526. 30290461 10.1586/17446651.1.4.517

[R39] Farrant M, and Nusser Z (2005). Variations on an inhibitory theme: phasic and tonic activation of GABA(A) receptors. Nat. Rev. Neurosci 6, 215–229. 15738957 10.1038/nrn1625

[R40] Fatemi SH, Aldinger KA, Ashwood P, Bauman ML, Blaha CD, Blatt GJ, Chauhan A, Chauhan V, Dager SR, Dickson PE, (2012). Consensus paper: pathological role of the cerebellum in autism. Cerebellum 11, 777–807. 22370873 10.1007/s12311-012-0355-9 PMC3677555

[R41] Ferando I, and Mody I (2013). Altered gamma oscillations during pregnancy through loss of δ subunit-containing GABA(A) receptors on parvalbumin interneurons. Front. Neural Circuits 7, 144. 24062647 10.3389/fncir.2013.00144 PMC3775147

[R42] Ferguson BR, and Gao W-J (2018). PV Interneurons: Critical Regulators of E/I Balance for Prefrontal Cortex-Dependent Behavior and Psychiatric Disorders. Front. Neural Circuits 12, 37. 29867371 10.3389/fncir.2018.00037 PMC5964203

[R43] File SE (1980). The use of social interaction as a method for detecting anxiolytic activity of chlordiazepoxide-like drugs. J. Neurosci. Methods 2, 219–238. 6120260 10.1016/0165-0270(80)90012-6

[R44] File SE, and Hyde JR (1978). Can social interaction be used to measure anxiety? Br. J. Pharmacol 62, 19–24. 563752 10.1111/j.1476-5381.1978.tb07001.x PMC1667770

[R45] Forman CJ, Tomes H, Mbobo B, Burman RJ, Jacobs M, Baden T, and Raimondo JV (2017). Openspritzer: an open hardware pressure ejection system for reliably delivering picolitre volumes. Sci. Rep 7, 2188. 28526883 10.1038/s41598-017-02301-2 PMC5438373

[R46] Fujita H, Kodama T, and Lac SD (2020). Modular output circuits of the fastigial nucleus mediate diverse motor and nonmotor functions of the cerebellar vermis. eLife 9, e58613. 32639229 10.7554/eLife.58613 PMC7438114

[R47] Fünfschilling U, and Reichardt LF (2002). Cre-mediated recombination in rhombic lip derivatives. Genesis 33, 160–169. 12203913 10.1002/gene.10104 PMC2710124

[R48] Füzesi T, Daviu N, Wamsteeker Cusulin JI, Bonin RP, and Bains JS (2016). Hypothalamic CRH neurons orchestrate complex behaviours after stress. Nat. Commun 7, 11937. 27306314 10.1038/ncomms11937 PMC4912635

[R49] Gandelman R (1973). Maternal behavior in the mouse: effect of estrogen and progesterone. Physiol. Behav 10, 153–155. 4735326 10.1016/0031-9384(73)90101-7

[R50] Giovannucci A, Badura A, Deverett B, Najafi F, Pereira TD, Gao Z, Ozden I, Kloth AD, Pnevmatikakis E, Paninski L, (2017). Cerebellar granule cells acquire a widespread predictive feedback signal during motor learning. Nat. Neurosci 20, 727–734. 28319608 10.1038/nn.4531 PMC5704905

[R51] Glykys J, Mann EO, and Mody I (2008). Which GABA(A) receptor subunits are necessary for tonic inhibition in the hippocampus? J. Neurosci 28, 1421–1426. 18256262 10.1523/JNEUROSCI.4751-07.2008 PMC6671570

[R52] Goldstein JM, Jerram M, Abbs B, Whitfield-Gabrieli S, and Makris N (2010). Sex differences in stress response circuitry activation dependent on female hormonal cycle. J. Neurosci 30, 431–438. 20071507 10.1523/JNEUROSCI.3021-09.2010 PMC2827936

[R53] Goldstein JM, Cherkerzian S, Tsuang MT, and Petryshen TL (2013). Sex differences in the genetic risk for schizophrenia: history of the evidence for sex-specific and sex-dependent effects. Am. J. Med. Genet. B. Neuropsychiatr. Genet 162B, 698–710. 24132902 10.1002/ajmg.b.32159

[R54] Gonzalo-Ruiz A, Leichnetz GR, and Hardy SG (1990). Projections of the medial cerebellar nucleus to oculomotor-related midbrain areas in the rat: an anterograde and retrograde HRP study. J. Comp. Neurol 296, 427–436. 1694191 10.1002/cne.902960308

[R55] Gunn BG, Brown AR, Lambert JJ, and Belelli D (2011). Neurosteroids and GABA(A) Receptor Interactions: A Focus on Stress. Front. Neurosci 5, 131. 22164129 10.3389/fnins.2011.00131 PMC3230140

[R56] Guo C, Witter L, Rudolph S, Elliott HL, Ennis KA, and Regehr WG (2016). Purkinje Cells Directly Inhibit Granule Cells in Specialized Regions of the Cerebellar Cortex. Neuron 91, 1330–1341. 27593180 10.1016/j.neuron.2016.08.011 PMC5853127

[R57] Hashimoto M, Yamanaka A, Kato S, Tanifuji M, Kobayashi K, and Yaginuma H (2018). Anatomical Evidence for a Direct Projection from Purkinje Cells in the Mouse Cerebellar Vermis to Medial Parabrachial Nucleus. Front. Neural Circuits 12, 6. 29467628 10.3389/fncir.2018.00006 PMC5808303

[R58] Hilber P, Lorivel T, Delarue C, and Caston J (2004). Stress and anxious-related behaviors in Lurcher mutant mice. Brain Res 1003, 108–112. 15019569 10.1016/j.brainres.2004.01.008

[R59] Hillerer KM, Neumann ID, and Slattery DA (2012). From stress to postpartum mood and anxiety disorders: how chronic peripartum stress can impair maternal adaptations. Neuroendocrinology 95, 22–38. 22042058 10.1159/000330445

[R60] Hines RM, Davies PA, Moss SJ, and Maguire J (2012). Functional regulation of GABAA receptors in nervous system pathologies. Curr. Opin. Neurobiol 22, 552–558. 22036769 10.1016/j.conb.2011.10.007 PMC3846183

[R61] Huang C-C, Sugino K, Shima Y, Guo C, Bai S, Mensh BD, Nelson SB, and Hantman AW (2013). Convergence of pontine and proprioceptive streams onto multimodal cerebellar granule cells. eLife 2, e00400. 23467508 10.7554/eLife.00400 PMC3582988

[R62] Iglói K, Doeller CF, Paradis A-L, Benchenane K, Berthoz A, Burgess N, and Rondi-Reig L (2015). Interaction Between Hippocampus and Cerebellum Crus I in Sequence-Based but not Place-Based Navigation. Cereb. Cortex 25, 4146–4154. 24947462 10.1093/cercor/bhu132 PMC4886832

[R63] Ishikawa T, Shimuta M, and Häusser M (2015). Multimodal sensory integration in single cerebellar granule cells in vivo. eLife 4, e12916. 26714108 10.7554/eLife.12916 PMC4798944

[R64] Jechlinger M, Pelz R, Tretter V, Klausberger T, and Sieghart W (1998). Subunit composition and quantitative importance of hetero-oligomeric receptors: GABAA receptors containing alpha6 subunits. J. Neurosci 18, 2449–2457. 9502805 10.1523/JNEUROSCI.18-07-02449.1998 PMC6793083

[R65] Jones A, Korpi ER, McKernan RM, Pelz R, Nusser Z, Mäkelä R, Mellor JR, Pollard S, Bahn S, Stephenson FA, (1997). Ligand-gated ion channel subunit partnerships: GABAA receptor alpha6 subunit gene inactivation inhibits delta subunit expression. J. Neurosci 17, 1350–1362. 9006978 10.1523/JNEUROSCI.17-04-01350.1997 PMC6793744

[R66] Kim YS, Woo J, Lee CJ, and Yoon B-E (2017). Decreased Glial GABA and Tonic Inhibition in Cerebellum of Mouse Model for Attention-Deficit/Hyperactivity Disorder (ADHD). Exp. Neurobiol 26, 206–212. 28912643 10.5607/en.2017.26.4.206 PMC5597551

[R67] Kinlein SA, Wilson CD, and Karatsoreos IN (2015). Dysregulated hypothalamic-pituitary-adrenal axis function contributes to altered endocrine and neurobehavioral responses to acute stress. Front. Psychiatry 6, 31. 25821436 10.3389/fpsyt.2015.00031 PMC4358064

[R68] Kloth AD, Badura A, Li A, Cherskov A, Connolly SG, Giovannucci A, Bangash MA, Grasselli G, Peñagarikano O, Piochon C, (2015). Cerebellar associative sensory learning defects in five mouse autism models. eLife 4, e06085. 26158416 10.7554/eLife.06085 PMC4512177

[R69] Korpi ER, Mihalek RM, Sinkkonen ST, Hauer B, Hevers W, Homanics GE, Sieghart W, and Lüddens H (2002). Altered receptor subtypes in the forebrain of GABA(A) receptor delta subunit-deficient mice: recruitment of gamma 2 subunits. Neuroscience 109, 733–743. 11927155 10.1016/s0306-4522(01)00527-9

[R70] Koutsikou S, Crook JJ, Earl EV, Leith JL, Watson TC, Lumb BM, and Apps R (2014). Neural substrates underlying fear-evoked freezing: the periaqueductal grey-cerebellar link. J. Physiol 592, 2197–2213. 24639484 10.1113/jphysiol.2013.268714 PMC4027863

[R71] Koyavski L, Panov J, Simchi L, Rayi PR, Sharvit L, Feuermann Y, and Kaphzan H (2019). Sex-Dependent Sensory Phenotypes and Related Transcriptomic Expression Profiles Are Differentially Affected by Angelman Syndrome. Mol. Neurobiol 56, 5998–6016. 30706369 10.1007/s12035-019-1503-8

[R72] Lee V, and Maguire J (2013). Impact of inhibitory constraint of interneurons on neuronal excitability. J. Neurophysiol 110, 2520–2535. 24027099 10.1152/jn.00047.2013 PMC3882765

[R73] Lee V, Sarkar J, and Maguire J (2014). Loss of Gabrd in CRH neurons blunts the corticosterone response to stress and diminishes stress-related behaviors. Psychoneuroendocrinology 41, 75–88. 24495609 10.1016/j.psyneuen.2013.12.011 PMC3947777

[R74] Lezak KR, Missig G, and Carlezon WA Jr. (2017). Behavioral methods to study anxiety in rodents. Dialogues Clin. Neurosci 19, 181–191. 28867942 10.31887/DCNS.2017.19.2/wcarlezon PMC5573562

[R75] Liu Z-P, He Q-H, Pan H-Q, Xu X-B, Chen W-B, He Y, Zhou J, Zhang W-H, Zhang J-Y, Ying X-P, (2017). Delta Subunit-Containing Gamma-Aminobutyric Acid A Receptor Disinhibits Lateral Amygdala and Facilitates Fear Expression in Mice. Biol. Psychiatry 81, 990–1002. 27591789 10.1016/j.biopsych.2016.06.022

[R76] Lorivel T, Roy V, and Hilber P (2014). Fear-related behaviors in Lurcher mutant mice exposed to a predator. Genes Brain Behav 13, 794–801. 25155577 10.1111/gbb.12173

[R77] Machado AS, Darmohray DM, Fayad J, Marques HG, and Carey MR (2015). A quantitative framework for whole-body coordination reveals specific deficits in freely walking ataxic mice. eLife 4, e07892. 26433022 10.7554/eLife.07892 PMC4630674

[R78] MacKenzie G, and Maguire J (2013). Neurosteroids and GABAergic signaling in health and disease. Biomol. Concepts 4, 29–42. 25436563 10.1515/bmc-2012-0033 PMC5469411

[R79] Madisen L, Zwingman TA, Sunkin SM, Oh SW, Zariwala HA, Gu H, Ng LL, Palmiter RD, Hawrylycz MJ, Jones AR, (2010). A robust and high-throughput Cre reporting and characterization system for the whole mouse brain. Nat. Neurosci 13, 133–140. 20023653 10.1038/nn.2467 PMC2840225

[R80] Maguire J, and Mody I (2007). Neurosteroid synthesis-mediated regulation of GABA(A) receptors: relevance to the ovarian cycle and stress. J. Neurosci 27, 2155–2162. 17329412 10.1523/JNEUROSCI.4945-06.2007 PMC6673487

[R81] Maguire J, and Mody I (2008). GABA(A)R plasticity during pregnancy: relevance to postpartum depression. Neuron 59, 207–213. 18667149 10.1016/j.neuron.2008.06.019 PMC2875248

[R82] Maguire J, and Mody I (2009). Steroid hormone fluctuations and GABA(A)R plasticity. Psychoneuroendocrinology 34 (Suppl 1), S84–S90. 19632051 10.1016/j.psyneuen.2009.06.019 PMC3399241

[R83] Maguire JL, Stell BM, Rafizadeh M, and Mody I (2005). Ovarian cycle-linked changes in GABA(A) receptors mediating tonic inhibition alter seizure susceptibility and anxiety. Nat. Neurosci 8, 797–804. 15895085 10.1038/nn1469

[R84] Maguire J, Ferando I, Simonsen C, and Mody I (2009). Excitability changes related to GABAA receptor plasticity during pregnancy. J. Neurosci 29, 9592–9601. 19641122 10.1523/JNEUROSCI.2162-09.2009 PMC2875247

[R85] Maison SF, Rosahl TW, Homanics GE, and Liberman MC (2006). Functional role of GABAergic innervation of the cochlea: phenotypic analysis of mice lacking GABA(A) receptor subunits alpha 1, alpha 2, alpha 5, alpha 6, beta 2, beta 3, or delta. J. Neurosci 26, 10315–10326. 17021187 10.1523/JNEUROSCI.2395-06.2006 PMC1806703

[R86] Manto M, Bower JM, Conforto AB, Delgado-García JM, da Guarda SNF, Gerwig M, Habas C, Hagura N, Ivry RB, Mariën P, (2012). Consensus paper: roles of the cerebellum in motor control–the diversity of ideas on cerebellar involvement in movement. Cerebellum 11, 457–487. 22161499 10.1007/s12311-011-0331-9 PMC4347949

[R87] Maras PM, Molet J, Chen Y, Rice C, Ji SG, Solodkin A, and Baram TZ (2014). Preferential loss of dorsal-hippocampus synapses underlies memory impairments provoked by short, multimodal stress. Mol. Psychiatry 19, 811–822. 24589888 10.1038/mp.2014.12 PMC4074447

[R88] Markowitz JE, Gillis WF, Beron CC, Neufeld SQ, Robertson K, Bhagat ND, Peterson RE, Peterson E, Hyun M, Linderman SW, (2018). The Striatum Organizes 3D Behavior via Moment-to-Moment Action Selection. Cell 174, 44–58.e17. 29779950 10.1016/j.cell.2018.04.019 PMC6026065

[R89] Marr D (1969). A theory of cerebellar cortex. J. Physiol 202, 437–470. 5784296 10.1113/jphysiol.1969.sp008820 PMC1351491

[R90] Martenson JS, Yamasaki T, Chaudhury NH, Albrecht D, and Tomita S (2017). Assembly rules for GABA_A_ receptor complexes in the brain. eLife 6, e27443. 28816653 10.7554/eLife.27443 PMC5577914

[R91] Martin BS, Corbin JG, and Huntsman MM (2014). Deficient tonic GABAergic conductance and synaptic balance in the fragile X syndrome amygdala. J. Neurophysiol 112, 890–902. 24848467 10.1152/jn.00597.2013 PMC4122738

[R92] Meera P, Wallner M, and Otis TS (2011). Molecular basis for the high THIP/gaboxadol sensitivity of extrasynaptic GABA(A) receptors. J. Neurophysiol 106, 2057–2064. 21795619 10.1152/jn.00450.2011 PMC3191842

[R93] Melón L, Hammond R, Lewis M, and Maguire J (2018). A Novel, Synthetic, Neuroactive Steroid Is Effective at Decreasing Depression-Like Behaviors and Improving Maternal Care in Preclinical Models of Postpartum Depression. Front. Endocrinol. (Lausanne) 9, 703. 30532739 10.3389/fendo.2018.00703 PMC6265503

[R94] Meltzer-Brody S, Colquhoun H, Riesenberg R, Epperson CN, Deligiannidis KM, Rubinow DR, Li H, Sankoh AJ, Clemson C, Schacterle A, (2018). Brexanolone injection in post-partum depression: two multicentre, double-blind, randomised, placebo-controlled, phase 3 trials. Lancet 392, 1058–1070. 30177236 10.1016/S0140-6736(18)31551-4

[R95] Menashe I, Grange P, Larsen EC, Banerjee-Basu S, and Mitra PP (2013). Co-expression profiling of autism genes in the mouse brain. PLoS Comput. Biol 9, e1003128. 23935468 10.1371/journal.pcbi.1003128 PMC3723491

[R96] Mercer AA, Palarz KJ, Tabatadze N, Woolley CS, and Raman IM (2016). Sex differences in cerebellar synaptic transmission and sex-specific responses to autism-linked Gabrb3 mutations in mice. eLife 5, e07596. 27077953 10.7554/eLife.07596 PMC4878876

[R97] Merikangas AK, and Almasy L (2020). Using the tools of genetic epidemiology to understand sex differences in neuropsychiatric disorders. Genes Brain Behav 19, e12660. 32348611 10.1111/gbb.12660 PMC7507200

[R98] Mihalek RM, Banerjee PK, Korpi ER, Quinlan JJ, Firestone LL, Mi ZP, Lagenaur C, Tretter V, Sieghart W, Anagnostaras SG, (1999). Attenuated sensitivity to neuroactive steroids in gamma-aminobutyrate type A receptor delta subunit knockout mice. Proc. Natl. Acad. Sci. USA 96, 12905–12910. 10536021 10.1073/pnas.96.22.12905 PMC23157

[R99] Mihalek RM, Bowers BJ, Wehner JM, Kralic JE, VanDoren MJ, Morrow AL, and Homanics GE (2001). GABA(A)-receptor delta subunit knockout mice have multiple defects in behavioral responses to ethanol. Alcohol. Clin. Exp. Res 25, 1708–1718. 11781502

[R100] Mitchell S, and Silver R (2003). Shunting inhibition modulates neuronal gain during synaptic excitation. Neuron 38, 433–445. 12741990 10.1016/s0896-6273(03)00200-9

[R101] Modgil A, Vien TN, Ackley MA, Doherty JJ, Moss SJ, and Davies PA (2019). Neuroactive Steroids Reverse Tonic Inhibitory Deficits in Fragile X Syndrome Mouse Model. Front. Mol. Neurosci 12, 15. 30804752 10.3389/fnmol.2019.00015 PMC6371020

[R102] Mody I, and Maguire J (2012). The reciprocal regulation of stress hormones and GABA(A) receptors. Front. Cell. Neurosci 6, 4. 22319473 10.3389/fncel.2012.00004 PMC3268361

[R103] Moreno-Rius J (2018). The cerebellum in fear and anxiety-related disorders. Prog. Neuropsychopharmacol. Biol. Psychiatry 85, 23–32. 29627508 10.1016/j.pnpbp.2018.04.002

[R104] Moreno-Rius J (2019). The cerebellum under stress. Front. Neuroendocrinol 54, 100774. 31348932 10.1016/j.yfrne.2019.100774

[R105] Newell A, Yang K, and Deng J (2016). Stacked Hourglass Networks for Human Pose Estimation. arXiv, 1603.06937, https://arxiv.org/abs/1603.06937.

[R106] Noirot E (1969). Serial order of maternal responses in mice. Anim. Behav 17, 547–550. 5370965 10.1016/0003-3472(69)90162-6

[R107] Nusser Z, Sieghart W, and Somogyi P (1998). Segregation of different GABAA receptors to synaptic and extrasynaptic membranes of cerebellar granule cells. J. Neurosci 18, 1693–1703. 9464994 10.1523/JNEUROSCI.18-05-01693.1998 PMC6792611

[R108] Nusser Z, Ahmad Z, Tretter V, Fuchs K, Wisden W, Sieghart W, and Somogyi P (1999). Alterations in the expression of GABAA receptor subunits in cerebellar granule cells after the disruption of the alpha6 subunit gene. Eur. J. Neurosci 11, 1685–1697. 10215922 10.1046/j.1460-9568.1999.00581.x

[R109] Ogris W, Lehner R, Fuchs K, Furtmüller B, Höger H, Homanics GE, and Sieghart W (2006). Investigation of the abundance and subunit composition of GABAA receptor subtypes in the cerebellum of alpha1-subunit-deficient mice. J. Neurochem 96, 136–147. 16277610 10.1111/j.1471-4159.2005.03509.x

[R110] Olmos-Serrano JL, Corbin JG, and Burns MP (2011). The GABA(A) receptor agonist THIP ameliorates specific behavioral deficits in the mouse model of fragile X syndrome. Dev. Neurosci 33, 395–403. 22067669 10.1159/000332884 PMC3254038

[R111] Orser BA (2006). Extrasynaptic GABAA receptors are critical targets for sedative-hypnotic drugs. J. Clin. Sleep Med 2, S12–S18. 17557502

[R112] Payne JL, and Maguire J (2019). Pathophysiological mechanisms implicated in postpartum depression. Front. Neuroendocrinol 52, 165–180. 30552910 10.1016/j.yfrne.2018.12.001 PMC6370514

[R113] Peng Z, Zhang N, Chandra D, Homanics GE, Olsen RW, and Houser CR (2014). Altered localization of the δ subunit of the GABAA receptor in the thalamus of α4 subunit knockout mice. Neurochem. Res 39, 1104–1117. 24352815 10.1007/s11064-013-1202-1 PMC4024081

[R114] Petersen HR, Jensen I, and Dam M (1983). THIP: a single-blind controlled trial in patients with epilepsy. Acta Neurol. Scand 67, 114–117. 6845976 10.1111/j.1600-0404.1983.tb04552.x

[R115] Pirker S, Schwarzer C, Wieselthaler A, Sieghart W, and Sperk G (2000). GABA(A) receptors: immunocytochemical distribution of 13 subunits in the adult rat brain. Neuroscience 101, 815–850. 11113332 10.1016/s0306-4522(00)00442-5

[R116] Pisanello F, Mandelbaum G, Pisanello M, Oldenburg IA, Sileo L, Markowitz JE, Peterson RE, Della Patria A, Haynes TM, Emara MS, (2017). Dynamic illumination of spatially restricted or large brain volumes via a single tapered optical fiber. Nat. Neurosci 20, 1180–1188. 28628101 10.1038/nn.4591 PMC5533215

[R117] Pisano TJ, Dhanerawala ZM, Kislin M, Bakshinskaya D, Engel EA, Lee J, de Oude NL, Venkataraju KU, Verpeut JL, Boele H-J, (2020). Parallel organization of cerebellar pathways to sensorimotor, associative, and modulatory forebrain. bioRxiv 10.1101/2020.03.06.979153.

[R118] Poulter MO, Barker JL, O’Carroll A-M, Lolait SJ, and Mahan LC (1992). Differential and transient expression of GABAA receptor alpha-subunit mRNAs in the developing rat CNS. J. Neurosci 12, 2888–2900. 1322978 10.1523/JNEUROSCI.12-08-02888.1992 PMC6575671

[R119] Rasmusson AM, Marx CE, Jain S, Farfel GM, Tsai J, Sun X, Geracioti TD, Hamner MB, Lohr J, Rosse R, (2017). A randomized controlled trial of ganaxolone in posttraumatic stress disorder. Psychopharmacology (Berl.) 234, 2245–2257. 28667510 10.1007/s00213-017-4649-y

[R120] Richardson BD, and Rossi DJ (2017). Recreational concentrations of alcohol enhance synaptic inhibition of cerebellar unipolar brush cells via pre- and postsynaptic mechanisms. J. Neurophysiol 118, 267–279. 28381493 10.1152/jn.00963.2016 PMC5498730

[R121] Rossi DJ, Hamann M, and Attwell D (2003). Multiple modes of GABAergic inhibition of rat cerebellar granule cells. J. Physiol 548, 97–110. 12588900 10.1113/jphysiol.2002.036459 PMC2342786

[R122] Sadakane K, Kondo M, and Nisimaru N (2000). Direct projection from the cardiovascular control region of the cerebellar cortex, the lateral nodulusuvula, to the brainstem in rabbits. Neurosci. Res 36, 15–26. 10678528 10.1016/s0168-0102(99)00103-0

[R123] Sathyanesan A, Zhou J, Scafidi J, Heck DH, Sillitoe RV, and Gallo V (2019). Emerging connections between cerebellar development, behaviour and complex brain disorders. Nat. Rev. Neurosci 20, 298–313. 30923348 10.1038/s41583-019-0152-2 PMC7236620

[R124] Schmahmann JD (2019). The cerebellum and cognition. Neurosci. Lett 688, 62–75. 29997061 10.1016/j.neulet.2018.07.005

[R125] Schmahmann JD, and Sherman JC (1998). The cerebellar cognitive affective syndrome. Brain 121, 561–579. 9577385 10.1093/brain/121.4.561

[R126] Schmahmann JD, Guell X, Stoodley CJ, and Halko MA (2019). The Theory and Neuroscience of Cerebellar Cognition. Annu. Rev. Neurosci 42, 337–364. 30939101 10.1146/annurev-neuro-070918-050258

[R127] Schmeisser MJ, Ey E, Wegener S, Bockmann J, Stempel AV, Kuebler A, Janssen A-L, Udvardi PT, Shiban E, Spilker C, (2012). Autistic-like behaviours and hyperactivity in mice lacking ProSAP1/Shank2. Nature 486, 256–260. 22699619 10.1038/nature11015

[R128] Schüle C, Nothdurfter C, and Rupprecht R (2014). The role of allopregnanolone in depression and anxiety. Prog. Neurobiol 113, 79–87. 24215796 10.1016/j.pneurobio.2013.09.003

[R129] Semyanov A, Walker MC, Kullmann DM, and Silver RA (2004). Tonically active GABA A receptors: modulating gain and maintaining the tone. Trends Neurosci 27, 262–269. 15111008 10.1016/j.tins.2004.03.005

[R130] Sequeira-Cordero A, Salas-Bastos A, Fornaguera J, and Brenes JC (2019). Behavioural characterisation of chronic unpredictable stress based on ethologically relevant paradigms in rats. Sci. Rep 9, 17403. 31758000 10.1038/s41598-019-53624-1 PMC6874551

[R131] Shen H, Gong QH, Aoki C, Yuan M, Ruderman Y, Dattilo M, Williams K, and Smith SS (2007). Reversal of neurosteroid effects at alpha4beta2-delta GABAA receptors triggers anxiety at puberty. Nat. Neurosci 10, 469–477. 17351635 10.1038/nn1868 PMC1858651

[R132] Smith SS (2013). α4βδ GABAA receptors and tonic inhibitory current during adolescence: effects on mood and synaptic plasticity. Front. Neural Circuits 7, 135. 24027497 10.3389/fncir.2013.00135 PMC3759753

[R133] Smith SS, Ruderman Y, Frye C, Homanics G, and Yuan M (2006). Steroid withdrawal in the mouse results in anxiogenic effects of 3alpha,5beta-THP: a possible model of premenstrual dysphoric disorder. Psychopharmacology (Berl.) 186, 323–333. 16193334 10.1007/s00213-005-0168-3 PMC2887339

[R134] Spigelman I, Li Z, Banerjee PK, Mihalek RM, Homanics GE, and Olsen RW (2002). Behavior and physiology of mice lacking the GABAA-receptor delta subunit. Epilepsia 43 (Suppl 5), 3–8. 10.1046/j.1528-1157.43.s.5.8.x 12121286

[R135] Spigelman I, Li Z, Liang J, Cagetti E, Samzadeh S, Mihalek RM, Homanics GE, and Olsen RW (2003). Reduced inhibition and sensitivity to neurosteroids in hippocampus of mice lacking the GABA(A) receptor delta subunit. J. Neurophysiol 90, 903–910. 12702713 10.1152/jn.01022.2002

[R136] Stell BM, and Mody I (2002). Receptors with different affinities mediate phasic and tonic GABA(A) conductances in hippocampal neurons. J. Neurosci 22, RC223. 12006605 10.1523/JNEUROSCI.22-10-j0003.2002 PMC6757628

[R137] Stell BM, Brickley SG, Tang CY, Farrant M, and Mody I (2003). Neuroactive steroids reduce neuronal excitability by selectively enhancing tonic inhibition mediated by delta subunit-containing GABAA receptors. Proc. Natl. Acad. Sci. USA 100, 14439–14444. 14623958 10.1073/pnas.2435457100 PMC283610

[R138] Stoodley CJ, and Schmahmann JD (2018). Functional topography of the human cerebellum. Handb. Clin. Neurol 154, 59–70. 29903452 10.1016/B978-0-444-63956-1.00004-7

[R139] Stoodley CJ, D’Mello AM, Ellegood J, Jakkamsetti V, Liu P, Nebel MB, Gibson JM, Kelly E, Meng F, Cano CA, (2017). Altered cerebellar connectivity in autism and cerebellar-mediated rescue of autism-related behaviors in mice. Nat. Neurosci 20, 1744–1751. 29184200 10.1038/s41593-017-0004-1 PMC5867894

[R140] Stórustovu SI, and Ebert B (2006). Pharmacological characterization of agonists at delta-containing GABAA receptors: Functional selectivity for extrasynaptic receptors is dependent on the absence of gamma2. J. Pharmacol. Exp. Ther 316, 1351–1359. 16272218 10.1124/jpet.105.092403

[R141] Strekalova T, Spanagel R, Dolgov O, and Bartsch D (2005). Stress-induced hyperlocomotion as a confounding factor in anxiety and depression models in mice. Behav. Pharmacol 16, 171–180. 15864072 10.1097/00008877-200505000-00006

[R142] Strick PL, Dum RP, and Fiez JA (2009). Cerebellum and nonmotor function. Annu. Rev. Neurosci 32, 413–434. 19555291 10.1146/annurev.neuro.31.060407.125606

[R143] Ströhle A, Romeo E, di Michele F, Pasini A, Yassouridis A, Holsboer F, and Rupprecht R (2002). GABA(A) receptor-modulating neuroactive steroid composition in patients with panic disorder before and during paroxetine treatment. Am. J. Psychiatry 159, 145–147. 11772707 10.1176/appi.ajp.159.1.145

[R144] Supple WF Jr., and Kapp BS (1994). Anatomical and physiological relationships between the anterior cerebellar vermis and the pontine parabrachial nucleus in the rabbit. Brain Res. Bull 33, 561–574. 7514486 10.1016/0361-9230(94)90082-5

[R145] Thakkar MM, Winston S, and McCarley RW (2008). Effect of microdialysis perfusion of 4,5,6,7-tetrahydroisoxazolo-[5,4-c]pyridine-3-ol in the perifornical hypothalamus on sleep-wakefulness: role of delta-subunit containing extrasynaptic GABAA receptors. Neuroscience 153, 551–555. 18406065 10.1016/j.neuroscience.2008.02.053 PMC2601694

[R146] Thomas SA, and Palmiter RD (1997). Impaired maternal behavior in mice lacking norepinephrine and epinephrine. Cell 91, 583–592. 9393852 10.1016/s0092-8674(00)80446-8

[R147] Thompson RF (1986). The neurobiology of learning and memory. Science 233, 941–947. 3738519 10.1126/science.3738519

[R148] Tolin DF, and Foa EB (2006). Sex differences in trauma and posttraumatic stress disorder: a quantitative review of 25 years of research. Psychol. Bull 132, 959–992. 17073529 10.1037/0033-2909.132.6.959

[R149] Tsai PT (2016). Autism and cerebellar dysfunction: Evidence from animal models. Semin. Fetal Neonatal Med 21, 349–355. 27179922 10.1016/j.siny.2016.04.009

[R150] Tsai PT, Hull C, Chu Y, Greene-Colozzi E, Sadowski AR, Leech JM, Steinberg J, Crawley JN, Regehr WG, and Sahin M (2012). Autistic-like behaviour and cerebellar dysfunction in Purkinje cell Tsc1 mutant mice. Nature 488, 647–651. 22763451 10.1038/nature11310 PMC3615424

[R151] Tsai PT, Rudolph S, Guo C, Ellegood J, Gibson JM, Schaeffer SM, Mogavero J, Lerch JP, Regehr W, and Sahin M (2018). Sensitive Periods for Cerebellar-Mediated Autistic-like Behaviors. Cell Rep 25, 357–367.e4. 30304677 10.1016/j.celrep.2018.09.039 PMC6226056

[R152] Vaaga CE, Brown ST, and Raman IM (2020). Cerebellar modulation of synaptic input to freezing-related neurons in the periaqueductal gray. eLife 9, 411. 10.7554/eLife.54302 PMC7124251 32207681

[R153] Vicini S, Losi G, and Homanics GE (2002). GABA(A) receptor delta subunit deletion prevents neurosteroid modulation of inhibitory synaptic currents in cerebellar neurons. Neuropharmacology 43, 646–650. 12367609 10.1016/s0028-3908(02)00126-0

[R154] Wagner MJ, Kim TH, Savall J, Schnitzer MJ, and Luo L (2017). Cerebellar granule cells encode the expectation of reward. Nature 544, 96–100. 28321129 10.1038/nature21726 PMC5532014

[R155] Wang SS-H, Kloth AD, and Badura A (2014). The cerebellum, sensitive periods, and autism. Neuron 83, 518–532. 25102558 10.1016/j.neuron.2014.07.016 PMC4135479

[R156] Watson TC, Obiang P, Torres-Herraez A, Watilliaux A, Coulon P, Rochefort C, and Rondi-Reig L (2019). Anatomical and physiological foundations of cerebello-hippocampal interaction. eLife 8, 598. 10.7554/eLife.41896 PMC6579515 31205000

[R157] Wei W, Zhang N, Peng Z, Houser CR, and Mody I (2003). Perisynaptic localization of delta subunit-containing GABA(A) receptors and their activation by GABA spillover in the mouse dentate gyrus. J. Neurosci 23, 10650–10661. 14627650 10.1523/JNEUROSCI.23-33-10650.2003 PMC6740905

[R158] Werling DM, and Geschwind DH (2013). Sex differences in autism spectrum disorders. Curr. Opin. Neurol 26, 146–153. 23406909 10.1097/WCO.0b013e32835ee548 PMC4164392

[R159] Whissell PD, Lecker I, Wang D-S, Yu J, and Orser BA (2015). Altered expression of δGABAA receptors in health and disease. Neuropharmacology 88, 24–35. 25128850 10.1016/j.neuropharm.2014.08.003

[R160] Whitaker AM, and Gilpin NW (2015). Blunted hypothalamo-pituitary adrenal axis response to predator odor predicts high stress reactivity. Physiol. Behav 147, 16–22. 25824191 10.1016/j.physbeh.2015.03.033 PMC4461370

[R161] Willsey AJ, Sanders SJ, Li M, Dong S, Tebbenkamp AT, Muhle RA, Reilly SK, Lin L, Fertuzinhos S, Miller JA, (2013). Coexpression networks implicate human midfetal deep cortical projection neurons in the pathogenesis of autism. Cell 155, 997–1007. 24267886 10.1016/j.cell.2013.10.020 PMC3995413

[R162] Wiltgen BJ, Sanders MJ, Ferguson C, Homanics GE, and Fanselow MS (2005). Trace fear conditioning is enhanced in mice lacking the delta subunit of the GABAA receptor. Learn. Mem 12, 327–333. 15897254 10.1101/lm.89705 PMC1142462

[R163] Wiltschko AB, Johnson MJ, Iurilli G, Peterson RE, Katon JM, Pashkovski SL, Abraira VE, Adams RP, and Datta SR (2015). Mapping Sub-Second Structure in Mouse Behavior. Neuron 88, 1121–1135. 26687221 10.1016/j.neuron.2015.11.031 PMC4708087

[R164] Wiltschko AB, Tsukahara T, Zeine A, Anyoha R, Gillis WF, Markowitz JE, Peterson RE, Katon J, Johnson MJ, and Datta SR (2020). Revealing the structure of pharmacobehavioral space through motion sequencing. Nat. Neurosci Published online September 21, 2020. 10.1038/s41593-020-00706-3. PMC7606807 32958923

[R165] Wisden W, Laurie DJ, Monyer H, and Seeburg PH (1992). The distribution of 13 GABAA receptor subunit mRNAs in the rat brain. I. Telencephalon, diencephalon, mesencephalon. J. Neurosci 12, 1040–1062. 1312131 10.1523/JNEUROSCI.12-03-01040.1992 PMC6576059

[R166] Witter L, and De Zeeuw CI (2015). In vivo differences in inputs and spiking between neurons in lobules VI/VII of neocerebellum and lobule X of archaeo-cerebellum. Cerebellum 14, 506–515. 25735968 10.1007/s12311-015-0654-z PMC4612334

[R167] Wood KC, Blackwell JM, and Geffen MN (2017). Cortical inhibitory interneurons control sensory processing. Curr. Opin. Neurobiol 46, 200–207. 28938181 10.1016/j.conb.2017.08.018 PMC5693245

[R168] Wu X, Gangisetty O, Carver CM, and Reddy DS (2013). Estrous cycle regulation of extrasynaptic δ-containing GABA(A) receptor-mediated tonic inhibition and limbic epileptogenesis. J. Pharmacol. Exp. Ther 346, 146–160. 23667248 10.1124/jpet.113.203653 PMC3684839

[R169] Yoshizawa K, Okumura A, Nakashima K, Sato T, and Higashi T (2017). Role of allopregnanolone biosynthesis in acute stress-induced anxiety-like behaviors in mice. Synapse 71, e21978. 10.1002/syn.21978 28407365

[R170] Zhang N, Peng Z, Tong X, Lindemeyer AK, Cetina Y, Huang CS, Olsen RW, Otis TS, and Houser CR (2017a). Decreased surface expression of the δ subunit of the GABA_A_ receptor contributes to reduced tonic inhibition in dentate granule cells in a mouse model of fragile X syndrome. Exp. Neurol 297, 168–178. 28822839 10.1016/j.expneurol.2017.08.008 PMC5612918

[R171] Zhang W-H, Zhou J, Pan H-Q, Wang X-Y, Liu W-Z, Zhang J-Y, Yin X-P, and Pan B-X (2017b). δ Subunit-containing GABA_A_ receptor prevents overgeneralization of fear in adult mice. Learn. Mem 24, 381–384. 28716958 10.1101/lm.045856.117 PMC5516689

[R172] Zhu J-N, Li H-Z, Ding Y, and Wang J-J (2006). Cerebellar modulation of feeding-related neurons in rat dorsomedial hypothalamic nucleus. J. Neurosci. Res 84, 1597–1609. 16998921 10.1002/jnr.21059

