# Supplementary material for: Cerebellum-Specific Deletion of the GABAA Receptor δ Subunit Leads to Sex-Specific Disruption of Behavior
Source: Cell Rep. Author manuscript; Available in PMC 2020 Nov 29. (PMC7700496; doi:10.1016/j.celrep.2020.108338)
Supplement: 1 [file NIHMS1643874-supplement-1.pdf]

Cell Reports, Volume *33*

Supplemental Information

Cerebellum-Specific Deletion of the $GABA_A$ Receptor δ Subunit Leads to Sex-Specific Disruption of Behavior

Stephanie Rudolph, Chong Guo, Stan L. Pashkovski, Tomas Osorno, Winthrop F. Gillis, Jeremy M. Krauss, Hajnalka Nyitrai, Isabella Flaquer, Mahmoud El-Rifai, Sandeep Robert Datta, and Wade G. Regehr

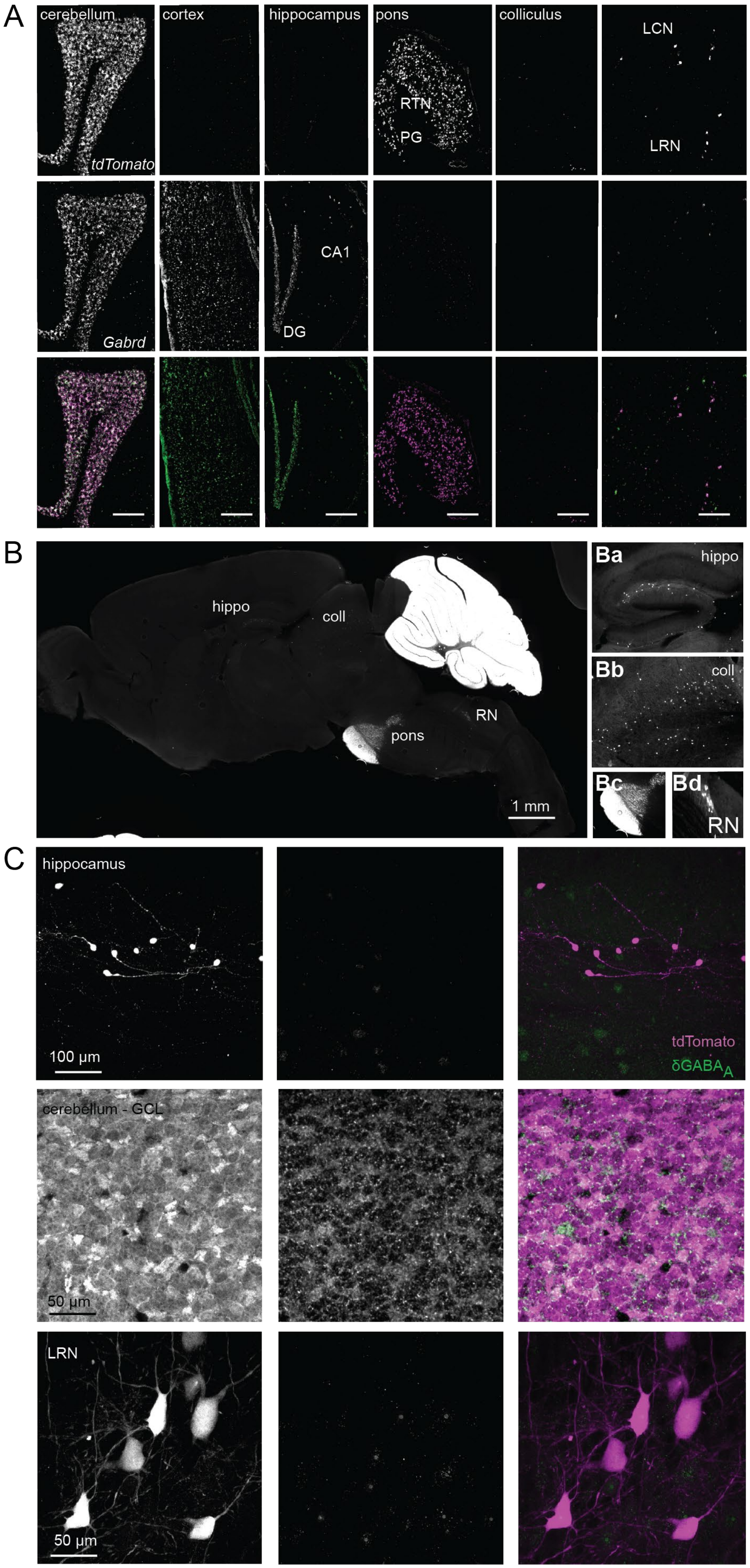

Figure S1: Fluorescence *in situ* hybridization (FISH) characterization of *tdTomato* and *Gabrd* co-expression in the Gabra6-Cre x Ai14 mouse line and immunohistochemical characterization of tdTomato and δGABA$_A$ co-expression in the Gabra6-Cre x Ai14 mouse line (related to Figure 1)

A) We observed strong co-expression of *tdTomato* (top row) and *Gabrd* transcripts (center row) in the GC layer of the cerebellum, absence of *tdTomato* expression in cortex and hippocampus (DG, dentate gyrus; CA1), strong expression of *tdTomato* in the pons (PG, pontine grey; RTN, reticulotegmental nucleus), but absence of *Gabrd* transcripts, absence of significant expression of either transcript in the colliculus, sparse *tdTomato* and *Gabrd* expression in brain stem nuclei (LRN, lateral reticular nucleus, LCN, lateral cervical nucleus), but no apparent overlap of the two markers. Scale bar denotes 200 µm.

B) Left, sagittal whole-brain section of Gabra6-Cre x Ai14 mouse reveals strong reporter expression in GCs of the cerebellum (note that both cell bodies in the GC layer and parallel fiber axons in the molecular layer are labelled), as well as sparse labeling in the hippocampus, colliculus, reticular nucleus, and strong expression in the pons. Insets (right) denote enlarged images of the respective brain regions.

C) Confocal images of tdTomato-positive putative interneurons in the stratum lacunosum and molecular layer of the hippocampus (top row), GC layer of the cerebellum (middle row), and lateral RN (bottom row). Immunolabeling of δGABA$_A$ is evident in membranes of GCs.

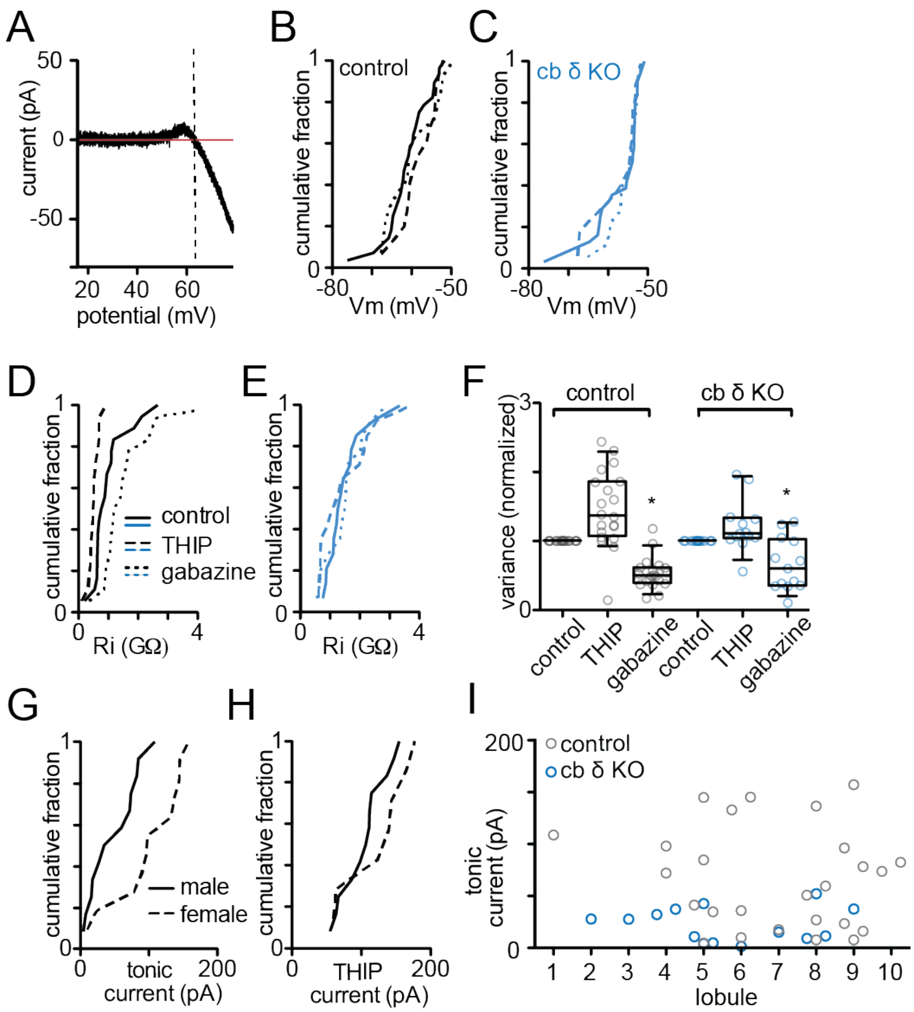

Figure S2: Electrical properties of control and cb δ KO granule cells (related to Figure 2)

A) Example trace of cell-attached recording used to determine membrane potential (Vm). Graph shows current trace with the linear fit of the leak current (horizontal red line) subtracted. The vertical dashed line indicates the reversal potential of the current, corresponding to Vm. See methods for experimental details.

B) Cumulative histograms of membrane potential (Vm) in control conditions (solid line), and in the presence of THIP (dashed line) or SR9931 (dotted line) for control animals.

C) Same as (B) but for cb δ KO mice. THIP and SR95531 did not affect Vm in control or cb δ KO GCs (control: n=28 control solution, n=15 THIP, n=14 gabazine; cb δ KO: n=31 control solution, n=17 THIP, n=18 gabazine, (one-way ANOVA with Dunnett's multiple comparison post-test).

D) Cumulative histogram of input resistance (R_i) in control conditions (solid line), or in the presence of THIP (dashed line) or SR995531 (dotted line). THIP decreased and SR95531 increased input resistance (R_i) in control GCs (n=18 for all conditions, $p<0.0001$).

E) In cb δ KO GCs THIP and SR95531 do not affect Ri ($p>0.05$). All data (D-E) describe matched observations, one-way ANOVA and Dunnett's multiple comparison post-test.

F) Box plot of normalized current variance in the presence of THIP or SR95531. THIP increased and SR95531 decreased current variance (measured as SD from mean current and normalized to control solution) in control granule cells (n=18 for all conditions, $p<0.0001$). In cb δ KO granule cells only SR95531 decreased current variance ($p<0.05$), while THIP has no significant effect ($p>0.05$; n=14 cells for all conditions). All data describes matched observations, one-way ANOVA and Dunnett's multiple comparison post-test)

G) Cumulative histograms of tonic current measured in males (solid line) and females (dashed line). Tonic current in females was higher than in males (males: n=12; females: n=11, $p<0.01$, KS test)

H) Cumulative histogram of the THIP evoked current measured in males (solid line) and females (dashed line). The THIP evoked current is similar in males and females (males: n=12; females: n=11, $p>0.1$, KS.

I) Tonic current amplitude recorded across lobules of the cerebellum shows variability but no lobule-specific differences.

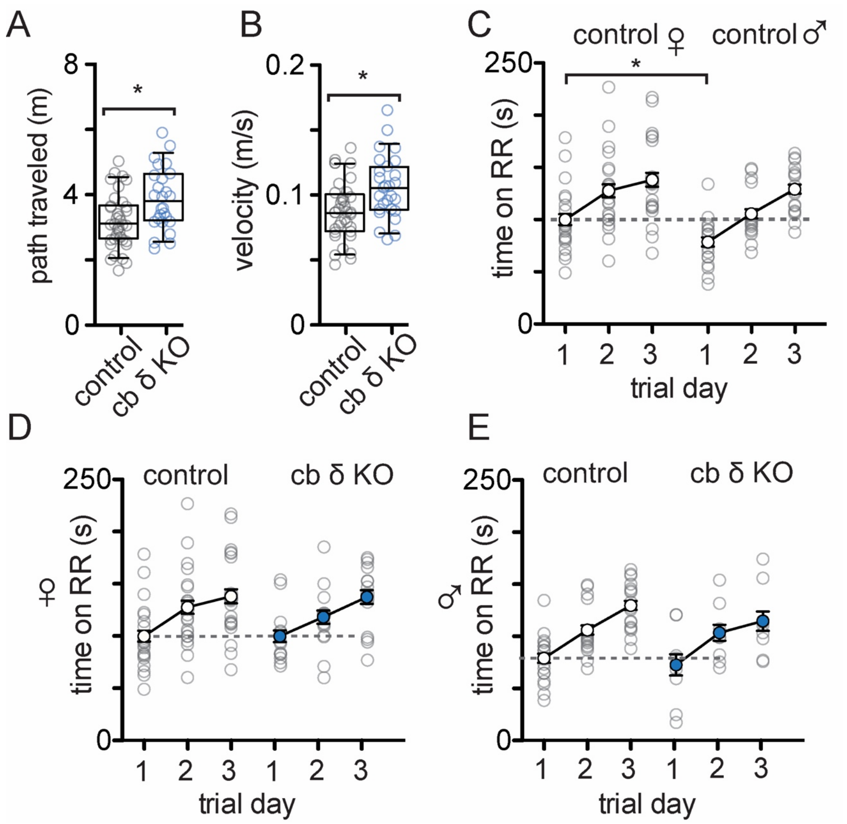

Figure S3: baseline locomotion and motor learning (related to Figure 3)

A) The average path travelled during an open field behavioral task (control: n=37 animals, animals, cb δ KO: n=27 animals, $p<0.007$, Mann-Whitney test). Box represents interquartile range and median, whiskers are shown as 10-90 percentile.

B) The average velocity during an open field behavioral task in control and cb δ KO animals (control: n=37 animals, cb δ KO: n=27 animals, $p>0.005$, Mann-Whitney test). Box represents interquartile range and median, whiskers are shown as 10-90 percentile.

C) Rotarod performance in male and female control animals. Females perform slightly better on training day 1 (females n=21, males n=17, $p<0.05$, Mann-Whitney test) but not on consecutive training days.

D) Rotarod performance of male control and cb δ KO animals (control n=17, cb δ KO n=8).

E) Rotarod performance of female control and cb δ KO animals (control n=21, cb δ KO n=14).

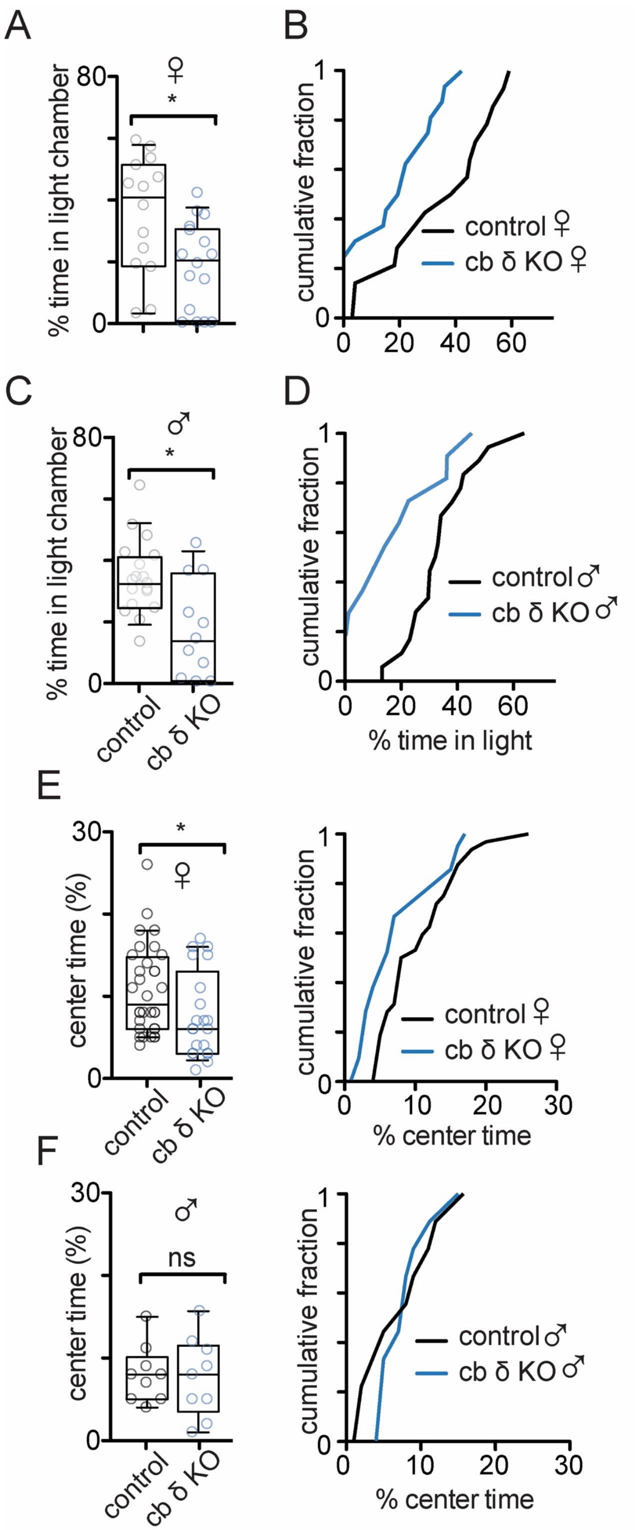

Figure S4 Anxiety-like behavior in cb δ KO males and females (related to figure 4)

A) Summary data of % time that females spent in the light compartment. Boxes denote interquartile range and median, whiskers represent 10-90 percentile. Circles show individual control (n=14, grey circles) and cb δ KO (n=16, blue circles) animals ($p<0.03$, Mann-Whitney test),

B) Cumulative fraction of time spent in the light compartment (control: black line, cb δ KO: blue line)

C) Summary data of % time that males spent in the light compartment. Circles show individual control (n=18, grey circles) and cb δ KO (n=11, blue circles) animals ($p<0.05$, Mann-Whitney test),

D) Cumulative fraction of time spent in the light compartment (control: black line, cb δ KO: blue line)

E) Summary data (left) and cumulative fraction (right) of % time female control (n=32) and cb δ KO animals (n=21) spent in the center ($p<0.04$, Mann-Whitney test)

F) Summary data (left) and cumulative fraction (right) of % time male control (n=9) and cb δ KO animals (n=9) spent in the center ($p>0.9$, Mann-Whitney test)

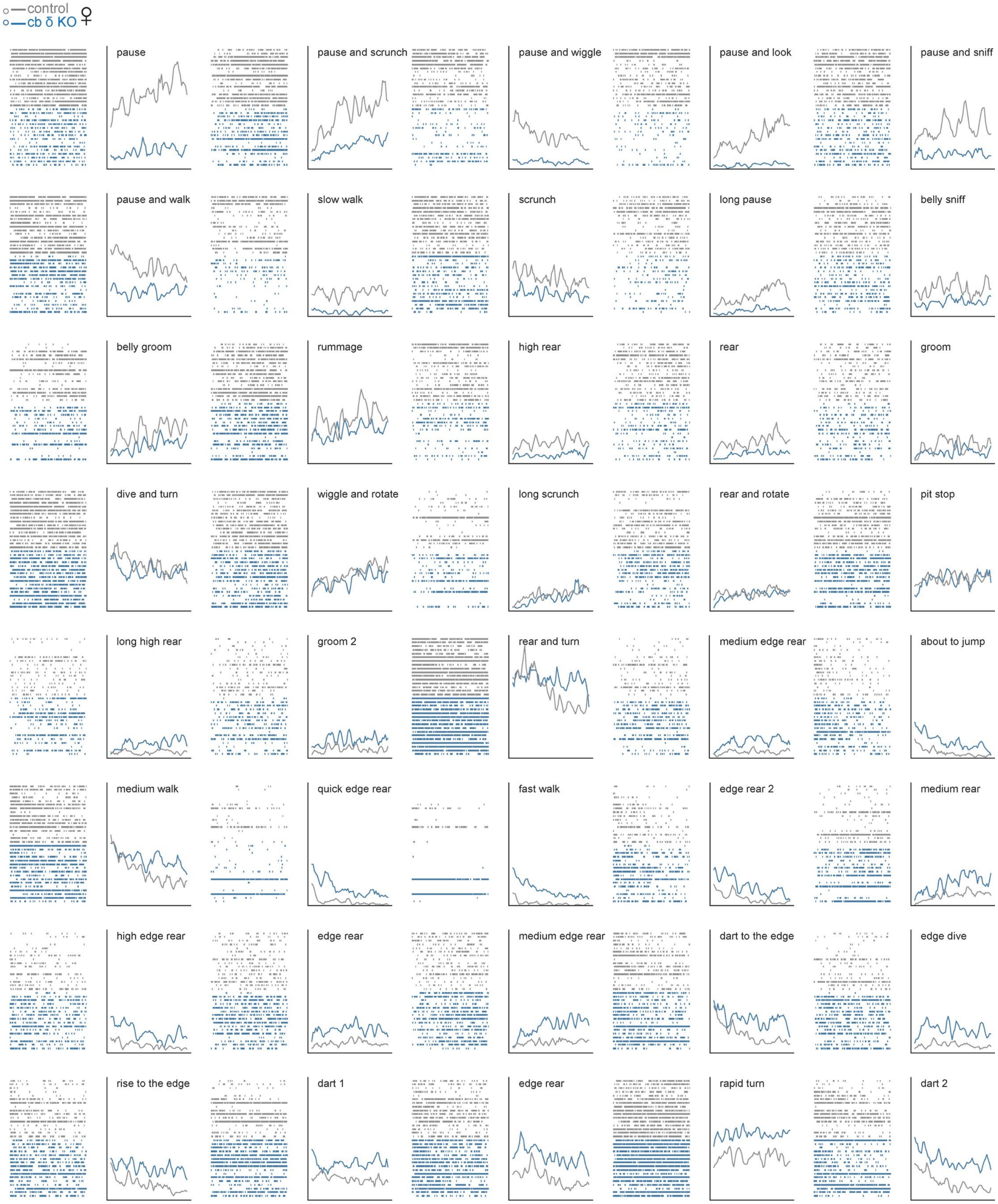

Figure S5: Behavioral syllables in the order of their frequency of occurrence (related to Figure 4)
The most frequent syllables in females are shown. Each panel shows syllable occurrence over the duration of the observation period in individual trials (left, tick plots) and median occurrence (right) in control (grey ticks and line) and cb δ KO mice (blue ticks and line).

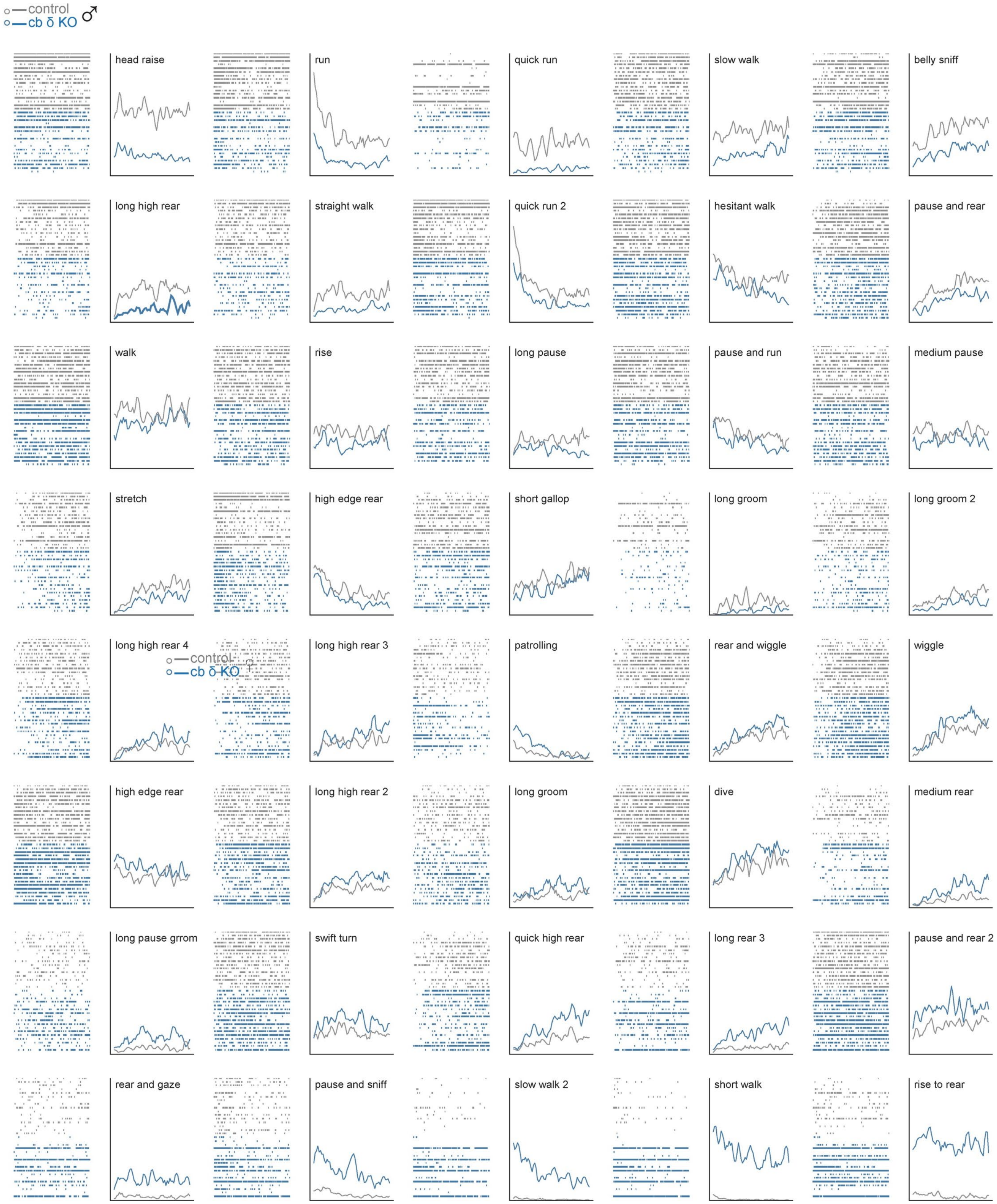

Figure S6: Behavioral syllables in the order of their frequency of occurrence (related to Figure 4)
The most frequent syllables in males are shown. Each panel shows syllable occurrence over the duration of the observation period in individual trials (left, tick plots) and median occurrence (right) in control (grey ticks and line) and cb δ KO mice (blue ticks and line).

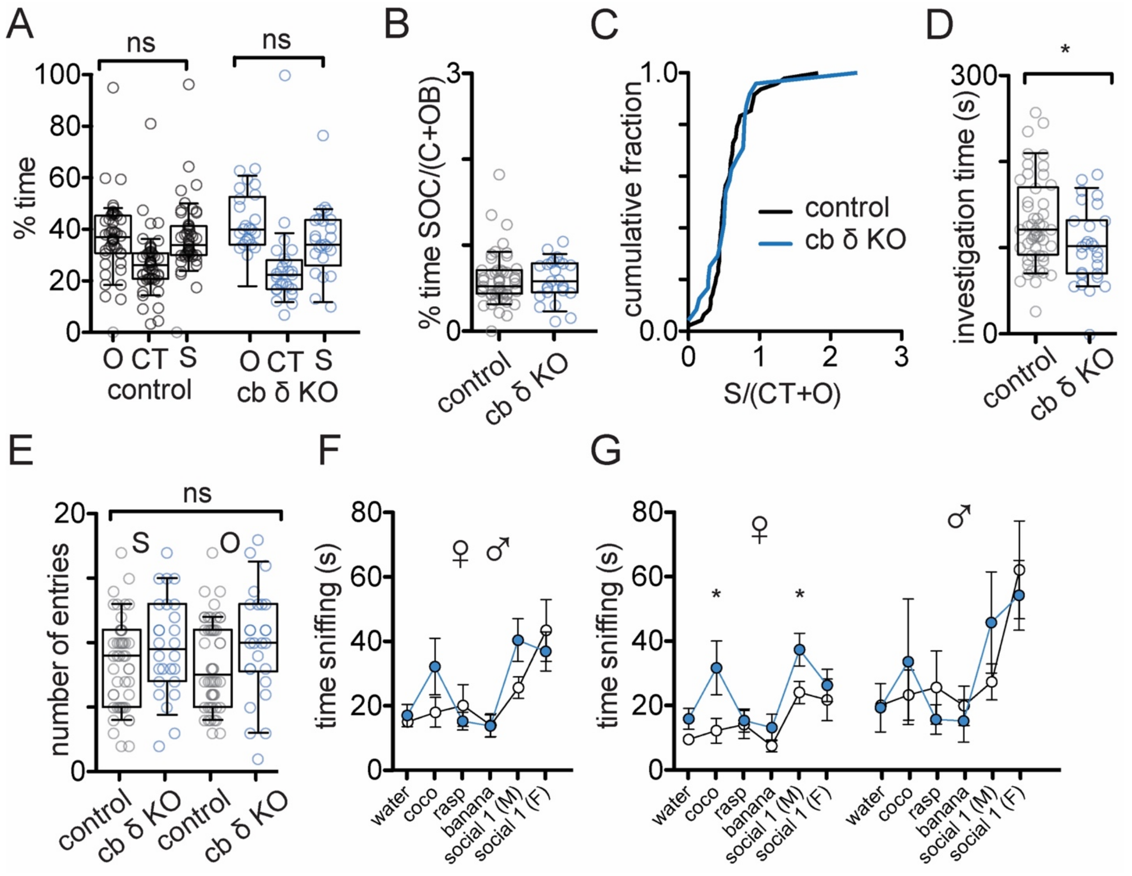

Figure S7: Baseline parameters for 3-chamber testing and olfaction (related to Figure 5)

A) Summary data of 3-chamber assay under baseline conditions. In the absence of a social stimulus and an object stimulus neither control nor cb δ KO mice show a preference for either compartment of the 3-chamber arena. (control, n=55, $p>0.2$; cb δ KO, n=30, $p>0.1$, Wilcoxon signed rank test)

B) In the absence of stimuli there is no difference in the S/(CTR+O) ratio between control and cb δ KO animals (control, n=55, cb δ KO, n=30, $p>0.5$, Mann-Whitney test)

C) Cumulative probability of S/(CTR+O) ratios ($p>0.2$, KS test).

D) Investigation time of social stimulus in control and cb δ KO animals (n=55, Mann-Whitney test, $p<0.04$)

E) Control and cb δ KO animals enter the object and social compartments with similar frequency (control, n=50, cb δ KO, n=30, entries social chamber, $p>0.2$, entries object chamber, $p>0.1$, Mann-Whitney test**)**

F) Average time spent sniffing four non-social (water, coconut, raspberry, banana) and two social cues (male and female urine) Control, n=24, cb δ KO, n=18, all odors, $p>0.05$, Mann-Whitney test.

G) Increased time spent sniffing was noted in cb δ KO females for coconut and male odor (coconut, $p<0.04$; male urine, $p<0.03$, all other odors in females and all odors in males $p>0.05$, Mann-Whitney test)

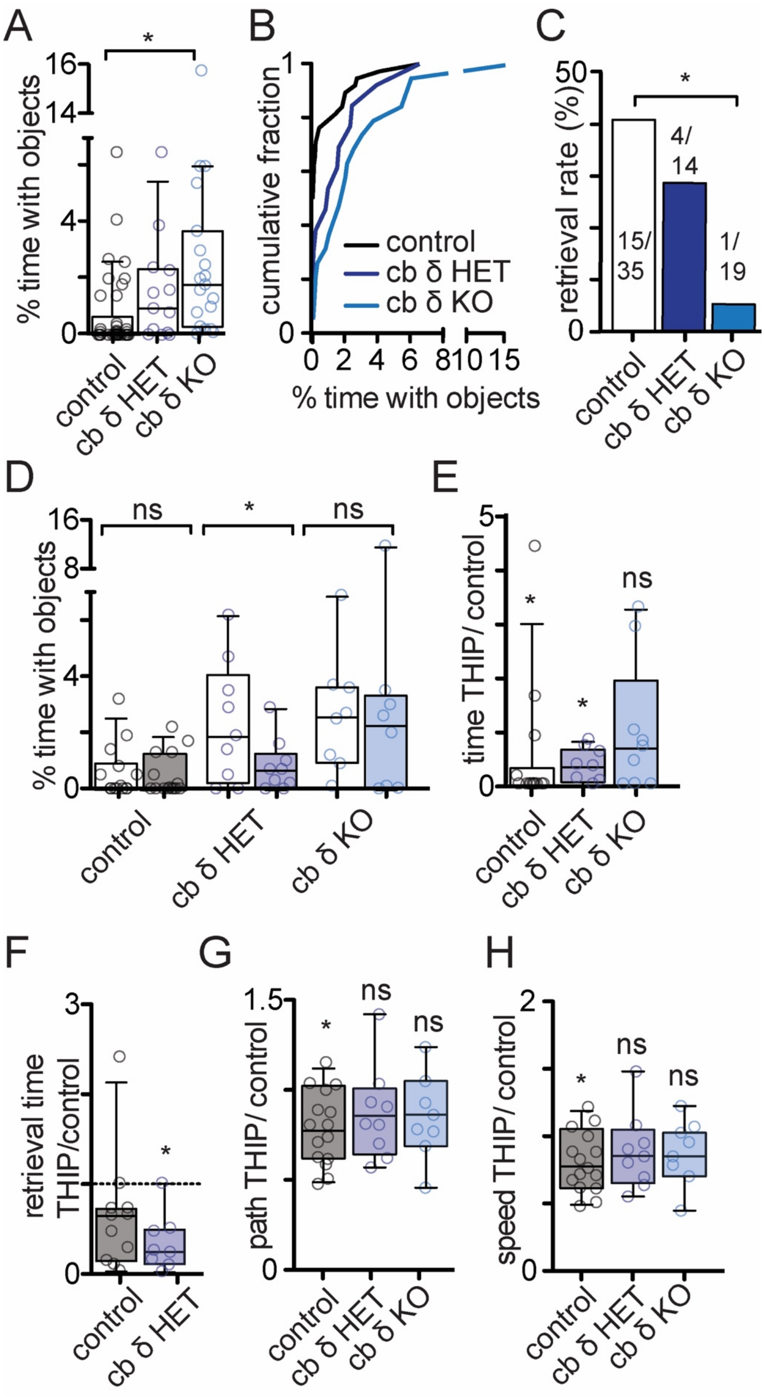

Figure S8: Additional analysis and rescue of parental behavior in virgin females (related to Figure 6)

A) Summary plot of time spent with pup-sized objects. cb δ KO mice show a greater interest in objects than controls. (control: grey, 0.7 ± 0.2 % time, n=37; cb δ HET: dark blue, 1.6 ± 0.5 % time, n=12, cb δ KO: blue, 2.8 ± 0.8 % time, n=19, Kruskal-Wallis test with Dunn's post-test, p<0.006)

B) Cumulative probability plot of time spent with objects.

C) Retrieval rate in control, cb δ HET and cb δ KO virgin females (control: n=35, cb δ HET: n=14, cb δ KO: n=19, p<0.009, Chi-Square test)

D) Time spent with objects under control conditions and after administration of THIP. THIP decreased the time cb δ HET females (2.7 ± 0.7 % time, THIP: 0.8 ± 0.3 p<0.02, Wilcoxon matched pairs signed rank test), but not control and cb δ KO females, spent with objects (grey circles, shaded grey, n=15: p>0.8; cb δ HET and cb δ HET THIP (dark blue circles, shaded dark blue, n=9): p<0.004; cb δ KO and cb δ KO THIP (light blue circles, shaded light blue, n=8): p>0.6; Wilcoxon matched-pairs signed rank test)

E) Ratio of time spent with objects in the presence and absence of THIP (control: grey circles/ shaded grey, n=15: p>0.7; cb δ HET: dark blue circles/ shaded dark blue, n=9, p<0.02; cb δ KO: light blue circles/ shaded light blue, n=8, p>0.6; Wilcoxon signed rank test)

F) Ratio of retrieval time under control conditions and after administration of THIP. THIP decreased the latency of retrieval in cb δ HET females but not control females (control: grey circles/ shaded grey, n=11: p>0.05; cb δ HET: dark blue circles/ shaded dark blue, n=8, p<0.05, Wilcoxon signed rank test)

G) Ratio of path travelled and H) average speed in the presence of THIP and under control conditions. THIP decreased distance travelled and speed in control but not cb δ HET and cb δ KO virgin females (p<0.05, Wilcoxon signed rank test)

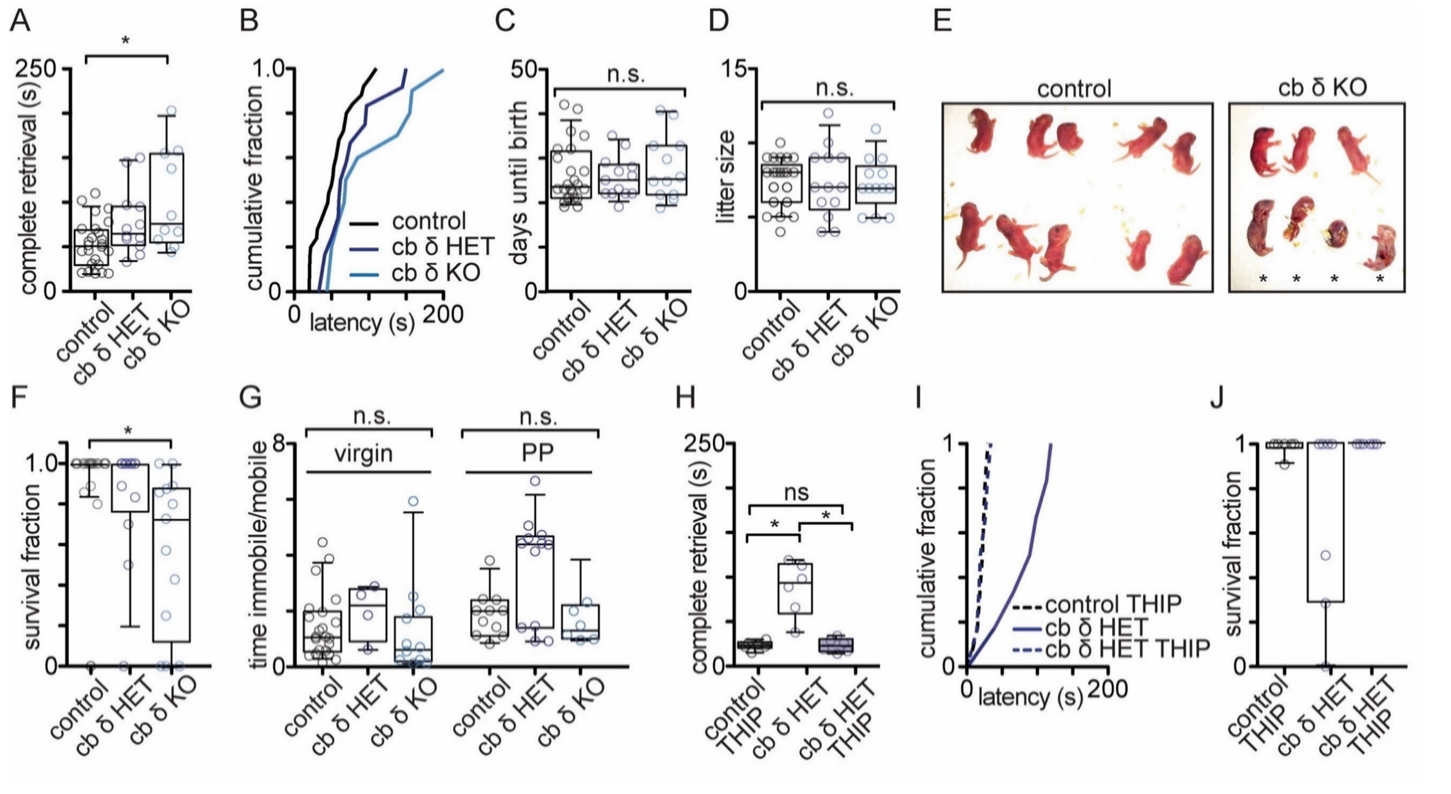

Figure S9: Retrieval, breeding, and depression-like behavior in postpartum females (related to Figure 6)

A) Time to complete retrieval of three pups to the nest for control, cb δ HET and cb δ KO mothers (51 ± 9 s, n=25; 78 ± 11 s, n=12; 103 ± 18 s; n=10, p>0.02, Kruskal-Wallis test with Dunn's post-test).

B) Cumulative plot of time to complete retrieval of three pups (p>0.05 control compared to cb δ HET, p<0.05 control compared to cb δ KO, KS test.

C) The number of days from the first day of mating until birth are similar in control (grey circles, n=24), cb δ HET (dark blue circles, n=13) and cb δ KO (blue circles, n=11, p>0.6, Kruskal-Wallis test).

D) Litter size at P0 is similar in control (grey circles, n=24), cb δ HET (dark blue circles, n=13) and cb δ KO (blue circles, n=11) females (Kruskal-Wallis test, p>0.9).

E) Example litter of a control (left) and cb δ KO female (right). Approximately 12 h after birth, pups of a control dam are viable, cleaned and have nursed. Pups of cb δ KO females are often neglected (not cleaned, amniotic sac not removed) and/or cannibalized. Asterisks denote dead pups.

F) Summary data of control, cb δ HET and cb δ KO litter survival fractions. (control: 0.9 ± 0.04, 26 litters; cb δ HET: 0.8 ± 0.1, 13 litters; cb δ KO: 0.6 ± 0.1, 13 litters, p>0.0002; Kruskal-Wallis test with Dunn's post-test).

G) Ratio of time immobile and time mobile during the Porsolt forced swim test in virgin and postpartum (PP) females. (virgins: p<0.2; PP dams: p<0.07, Kruskal Wallis test with Dunn's-post-test)

H) Time to complete retrieval of three pups to the nest for control dams that received THIP (23 ± 2 s, n=6), cb δ HET (87 ± 12 s, n=6) and cb δ HET dams that received THIP (23 ± 3 s, n=6; p>0.004, Kruskal-Wallis test with Dunn's post-test for cb δ HET compared to cb δ HET THIP, p<0.05 for control THIP compared to cb δ HET THIP).

I) Cumulative plot of time to complete retrieval of three pups (p<0.03 control THIP compared to cb δ HET, p<0.02 cb δ HET compared to cb δ HET THIP, p>0.08 control THIP compared to cb δ HET THIP)

J) Summary plot of pup survival fraction of litters born to control THIP (1.0 ± 0.01), cb δ HET (0.7 ± 0.2) and cb δ HET THIP dams (1 ± 0).

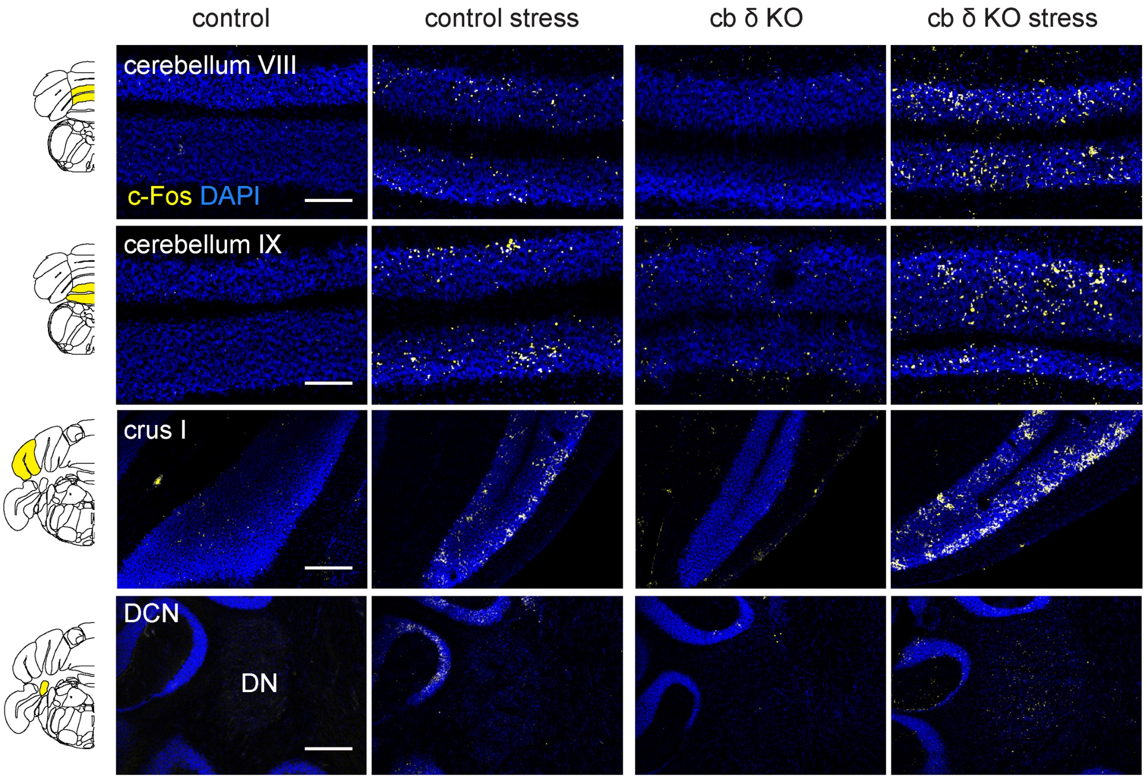

Figure S10: c-Fos labeling in different regions of the cerebellar cortex and the DCN (related to Figure 7)
Left, reference atlas images of coronal cerebellar sections, and the regions of the corresponding confocal images (right) are highlighted in yellow. Right, representative confocal images of unstressed and stressed control and cb δ KO animals (blue: DAPI, yellow: c-Fos). Example regions include lobules VIII, IX of the vermis, crus I of the hemispheres and the dentate nucleus of the DCN. Scale bar denotes 100 μm.

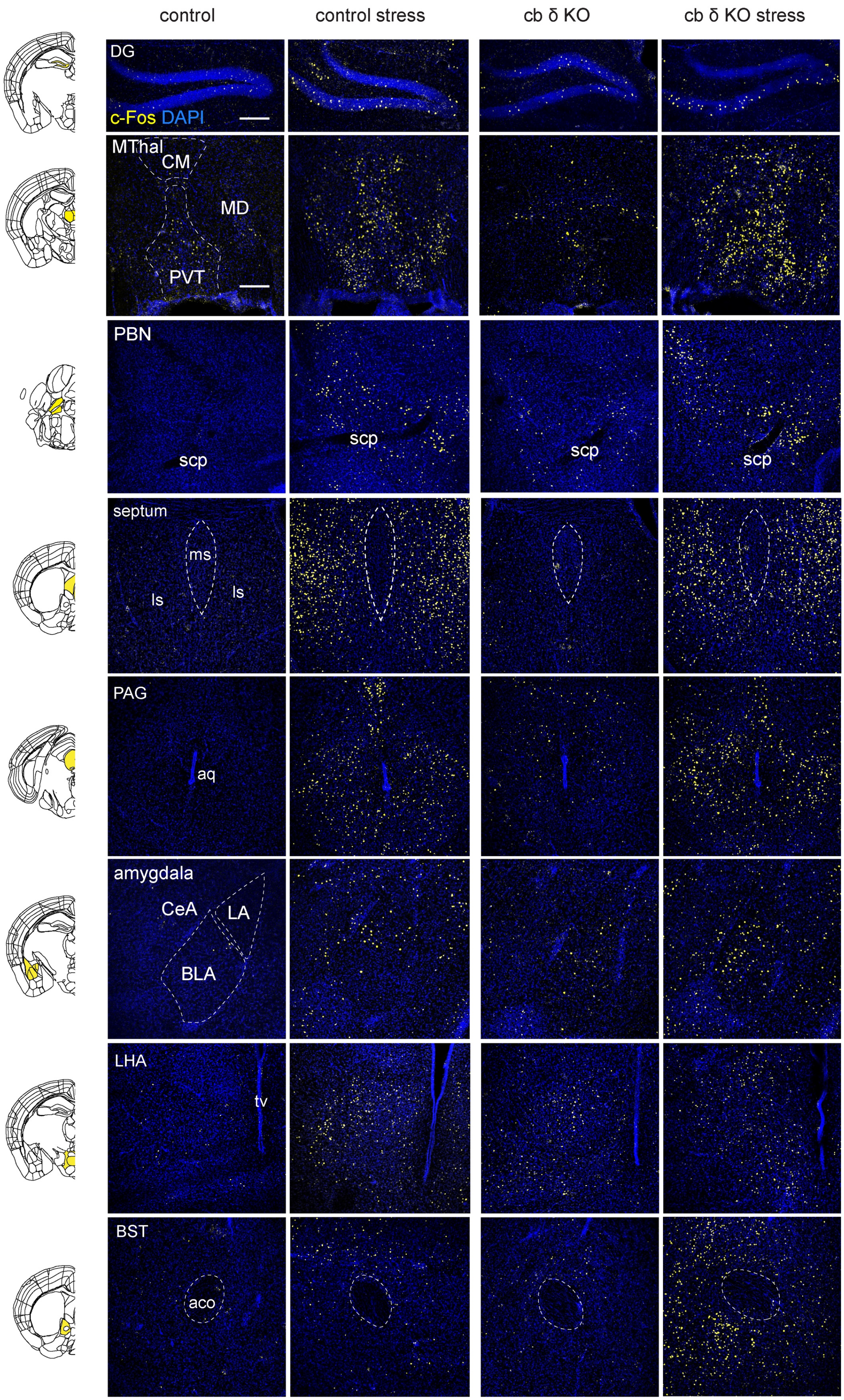

Figure S11: c-Fos labeling in additional cortical and subcortical brain regions (related to Figure 7)
Left, reference atlas images of coronal brain sections, and the regions of the corresponding confocal images (right) are highlighted in yellow. Right, representative confocal images of unstressed and stressed control and cb δ KO animals (blue: DAPI, yellow: c-Fos). Example regions include dentate gyrus (DG), medial thalamus (MDThal; PVT, paraventricular thalamus; MD, mediodorsal thalamus; CM, centromendial thalamus), parabrachial nucleus (PBN; scp superior cerebellar peducle), septum (ls, lateral septum; ms, medial septum); periaqueductal grey (PAG), amygdala (CeA, central amygdala; BLA, basolateral amygdala; LA, lateral amygdala), lateral hypothalamic region (LHA; tv, third ventricle), bed nucleus of the stria terminalis (BST; aco, anterior commissure). Scale bar denotes 200 µm (top row), and 100 µm (second row, applicable to all following panels).
